# Need for personalized monitoring of Parkinson’s disease: the perspectives of patients and specialized healthcare providers

**DOI:** 10.3389/fneur.2023.1150634

**Published:** 2023-05-04

**Authors:** Luc J. W. Evers, José M. Peeters, Bastiaan R. Bloem, Marjan J. Meinders

**Affiliations:** ^1^Center of Expertise for Parkinson & Movement Disorders, Department of Neurology, Donders Institute for Brain, Cognition and Behaviour, Radboud University Medical Center, Nijmegen, Netherlands; ^2^Institute for Computing and Information Sciences, Radboud University, Nijmegen, Netherlands; ^3^Scientific Center for Quality of Healthcare (IQ Healthcare), Radboud Institute for Health Sciences, Radboud University Medical Center, Nijmegen, Netherlands

**Keywords:** Parkinson‘s disease, remote monitoring, self-monitoring, wearable sensors, personalized care, disease monitoring

## Abstract

**Background:**

Digital tools such as wearable sensors may help to monitor Parkinson’s disease (PD) in daily life. To optimally achieve the expected benefits, such as personized care and improved self-management, it is essential to understand the perspective of both patients and the healthcare providers.

**Objectives:**

We identified the motivations for and barriers against monitoring PD symptoms among PD patients and healthcare providers. We also investigated which aspects of PD were considered most important to monitor in daily life, and which benefits and limitations of wearable sensors were expected.

**Methods:**

Online questionnaires were completed by 434 PD patients and 166 healthcare providers who were specialized in PD care (86 physiotherapists, 55 nurses, and 25 neurologists). To gain further understanding in the main findings, we subsequently conducted homogeneous focus groups with patients (*n* = 14), physiotherapists (*n* = 5), and nurses (*n* = 6), as well as individual interviews with neurologists (*n* = 5).

**Results:**

One third of the patients had monitored their PD symptoms in the past year, most commonly using a paper diary. Key motivations were: (1) discuss findings with healthcare providers, (2) obtain insight in the effect of medication and other treatments, and (3) follow the progression of the disease. Key barriers were: (1) not wanting to focus too much on having PD, (2) symptoms being relatively stable, and (3) lacking an easy-to-use tool. Prioritized symptoms of interest differed between patients and healthcare providers; patients gave a higher priority to fatigue, problems with fine motor movements and tremor, whereas professionals more frequently prioritized balance, freezing and hallucinations. Although both patients and healthcare providers were generally positive about the potential of wearable sensors for monitoring PD symptoms, the expected benefits and limitations varied considerably between groups and within the patient group.

**Conclusion:**

This study provides detailed information about the perspectives of patients, physiotherapists, nurses and neurologists on the merits of monitoring PD in daily life. The identified priorities differed considerably between patients and professionals, and this information is critical when defining the development and research agenda for the coming years. We also noted considerable differences in priorities between individual patients, highlighting the need for personalized disease monitoring.

## 1. Introduction

Parkinson’s disease (PD) is a chronic, progressive neurodegenerative disease with complex clinical presentation (1, 2). Patients may experience motor symptoms such as bradykinesia, rigidity, tremor and balance impairments, but also a wide range of non-motor symptoms, such as mood changes, cognitive decline, pain and sleep disturbance, and side effects of medication such as dyskinesia. Symptoms can differ considerably between patients, and both the nature and the impact of symptoms can vary markedly throughout the course of the disease (3). In current clinical practice, we mainly use self-reports (history taking, sometimes supplemented by diaries) and in-clinic observations to monitor the presence and severity of symptoms, as well as the response to treatment. These episodic assessments do not always provide a representative and complete picture of the patient’s actual functioning in daily life, for example due to recall bias (4) and observer effects (5). Remote monitoring tools such as wearable sensors may partly fill this gap and provide opportunities for personalized care, telemedicine and improved self-management (6, 7).

To deliver on these promises, it is essential that such tools address specific needs experienced by PD patients and their healthcare providers. This requires a thorough understanding of the diverse perspectives on symptom monitoring and wearable sensors of all stakeholders involved (8). PD patients vary in terms of experienced symptoms, but also with regard to coping strategies and personal treatment goals (9). Moreover, professionals from multiple disciplines can be involved in PD care, including neurologists, physiotherapists, nurses, speech therapists, general practitioners and many others, with each discipline focusing on different aspects (10). Our current understanding of the motivations and barriers for symptom monitoring of PD and for using wearable sensors is fragmented, and the focus has thus far mainly been on the perspective of patients (11, 12).

Because health monitoring behavior is not limited to patients, some useful insights can be obtained from studies in the general population. Using a survey among 150 self-trackers, five different motivations for self-tracking were identified, consisting of self-design (possibilities of self-optimization), self-discipline (self-gratification possibilities), and self-healing (independence of traditional medical treatment), self-entertainment (pleasure-bringing aspects), and self-association (sharing results with others and being part of a community) (13). This framework has not yet been evaluated in PD patients, but other studies have shown that specific motivations of PD patients with experience in self-tracking include the desire to better understand their disease, understand the effects of medication intake, and share information with healthcare providers (11, 12). The symptoms of interest among patients varied between studies, often including slowness of movements, tremor, stiffness, lack of energy, and sleep (11, 12, 14). In these studies, the barriers among patients who did not engage in self-tracking activities (36 to 51%) were not investigated. A better understanding of experienced barriers could provide useful strategies to engage and support these patients as well, and offer useful insights in the potential (and limitations) of monitoring tools such as wearable sensors.

The perspective of different PD healthcare providers on symptom monitoring, and how this relates to the perspective of patients has received little attention so far. Studies on symptom monitoring in PD that included healthcare providers did not differentiate between different disciplines (i.e., neurologists, physiotherapists, etc.) (14, 15), or aimed to reach consensus between healthcare providers and patients (14). We approach the problem from a different angle, and hypothesize that the different groups may represent unique needs, potentially requiring different solutions.

The aim of this study is to provide insights that can fuel the development of remote monitoring tools that address specific needs experienced by patients and/or healthcare providers. Specifically, our objectives were to identify the motivations for and barriers to monitoring PD symptoms, and to better understand the expected benefits and limitations of wearable sensors. In addition, we aimed to assess which aspects of PD are considered most important to be monitored in daily life. Finally, we aimed to compare the perspectives of PD patients and healthcare providers specialized in PD (physiotherapists, nurses, and neurologists).

## 2. Methods

### 2.1. Study design

We used a two-phase, explanatory mixed method design, consisting of online surveys, and subsequent homogeneous focus groups and interviews among the different stakeholders to gain further understanding in the domains of interest that were identified in the preceding surveys (16, 17). We focus on the perspective of PD patients, as well as that of healthcare providers that are most frequently involved in PD care in the Netherlands, i.e., neurologists, physiotherapists and Parkinson nurses. The study was approved by the local medical ethics committee (Commissie Mensgebonden Onderzoek, regio Arnhem-Nijmegen; file number 2015–1776). All participants provided informed consent prior to participation.

### 2.2. Participants

Seven hundred and eleven persons with PD were invited by email to participate in the online survey. All invitees were on the waiting list to be included in the Parkinson@Home study (18). Various recruitment strategies were used, including advertisements in the Dutch Parkinson Patient Association magazine and on social media, visits to support groups, and through physiotherapists specialized in PD care. Inclusion criteria were broad; participants were only asked to confirm that they were diagnosed with PD by a neurologist at the start of the survey. At the end of the survey, participants were invited to participate in subsequent focus groups.

We also included healthcare providers who were specialized in PD care. We chose to focus on the perspectives of neurologists, physiotherapists and Parkinson nurses, because the survey among patients showed that these healthcare providers are most frequently involved in PD care in the Netherlands (Table 1). To ensure that all included healthcare providers had sufficient experience in PD care, we only included members of the Dutch ParkinsonNet, a nationwide network of healthcare professionals who have received dedicated training in managing persons with PD (19). The invitations for the online survey were sent by email to 85 neurologists, 156 physiotherapists and 163 nurses. Participants for the focus groups and interviews with healthcare providers were recruited from the responses to the survey and *via* ParkinsonNet.

**Table 1:** Characteristics of the early PD (<6 years since diagnosis) and late PD (≥6 years since diagnosis) groups.

	Early PD patients (*n* = 207)	Late PD patients (*n* = 222)
Age (years), mean (SD)	67.3 (8.6)	69.1 (8.1)
Gender (men), *n* (%)	146 (71%)	136 (61%)
Use of PD medication (% yes)	201 (97%)	221 (99%)
Time since diagnosis of PD (years), mean (SD)	3.8 (1.5)	12.6 (7.3)
Healthcare providers seen in past year for PD (% yes)		
Neurologist	204 (99%)	215 (97%)
Physiotherapist	161 (78%)	189 (85%)
Parkinson nurse	143 (69%)	164 (74%)
General practitioner	80 (39%)	95 (43%)
Occupational therapist	45 (22%)	56 (25%)
Speech therapist	51 (25%)	44 (20%)
Dietitian	20 (10%)	32 (14%)
Other (including psychologist, revalidation specialist, neurosurgeon)	32 (16%)	40 (18%)

PD, Parkinson’s disease; SD, standard deviation.

### 2.3. Survey development

We developed two surveys: one for patients and one for healthcare providers. The surveys consisted of a combination of validated questionnaires and custom-developed questions, on the following domains: current use of monitoring tools, motivations for and barriers to monitoring PD, relevant aspects to monitor, and expected benefits and limitations of wearable sensors for monitoring PD.

First, the surveys addressed the participants’ experience with symptom monitoring, including the use of PD monitoring tools. Among patients, we assessed motivations for and barriers to self-monitoring PD symptoms using open-ended questions, and using the validated 19-item motivations for self-tracking scale (13). This scale consists of 19 items answered on a Likert scale ranging from 0 (“disagree strongly”) to 4 (“agree strongly”). A five-factor structure was identified by the developers, consisting of self-entertainment (five items, e.g., “I enjoy getting lost in totally in self-tracking activities”), self-association (four items, e.g., “I want to help/inspire others”), self-design (five items, e.g., “I want to control what I am doing with my life,”) self-discipline (three items, e.g., “It motivates me to keep on working for a goal”), and self-healing (two items, e.g., “I do not trust the healthcare system/classic therapies”). Next, we asked both patients and healthcare providers to indicate which symptoms, and which factors that influence symptoms, they found most useful to monitor in daily life. Participants were instructed to select a top 3 from a predefined list. The symptom list was based on the Non-Motor Symptom Questionnaire (NMS-Quest) (20) and the MDS-UPDRS part II, with some additions from the patient survey used by Mathur et al. (11). All items were phrased in understandable language, and medical terms for symptoms were avoided as much as possible. To facilitate the comparison, the items were identical between the patient and healthcare provider surveys. For patients, the items were personalized according to which symptoms they had ever experienced. To assess which factors explained the selection of specific symptoms, patients were also asked to make a top 3 of the most troublesome and a top 3 of the most strongly fluctuating symptoms. Finally, we explored the interest of healthcare providers in wearable sensors, including the expected benefits and limitations of this technology, using a combination of open- and closed-ended questions.

To assess whether participants could understand the questions and formulate appropriate answers, we performed cognitive interviews prior to deployment (21). These interviews were conducted face-to-face or by telephone, with five PD patients and four healthcare providers (two physiotherapists, one nurse, and one neurologist). During each session the assessor asked the participant to complete the draft survey, and to think out loud while doing so. Based on the assessor’s observations and the feedback from participants, we updated the survey after each session, until all questions were correctly understood.

The surveys were implemented using SurveyGizmo^1^, which allowed for the inclusion of advanced functionalities such as personalized drag-and-drop lists. The required completion time was approximately 30 min. The full surveys can be found in Appendix A.

### 2.4. Survey analysis

All data were analyzed separately for each stakeholder group. Persons with PD were divided into early (≤5 years since diagnosis) and late PD groups (>5 years since diagnosis) (3). All answers to open-ended questions were analyzed using thematic analysis with inductive coding (17). Quantitative outcomes were analyzed using descriptive statistics. Specifically, from the responses to the motivations for self-tracking scale, we calculated subtotals according to the identified five-factor structure (13). For each symptom, and for each factor that influences symptoms, we determined the percentage of participants who selected the item for their top 3. To examine differences between patients and healthcare providers, the average percentages of patients (early and late PD) were compared with the average percentages of healthcare providers (neurologists, physiotherapists and nurses). To assess which factors explained the patients’ selected three most important symptoms to monitor, we examined the correlation with the selected three most troublesome and three most strongly fluctuating symptoms (using Spearman’s ρ, applied to the percentages). We performed the quantitative analyses using SPSS (version 22.0), and we used Atlas.Ti (version 8.2.29) to support the qualitative analyses.

### 2.5. Design of focus groups and individual interviews

To gain a deeper understanding of the results of the survey and collect illustrative examples, we conducted homogeneous, semi-structured focus group discussions; two groups were organized with persons with PD, one group with physiotherapists specialized in PD, and one group with Parkinson nurses. We opted for focus groups because we expected that a group setting would stimulate further discussion about items that were considered relevant by more than one group member (22). The choice for *homogeneous* focus groups matches our hypothesis that the different stakeholders represent unique needs, which may require different solutions (i.e., the goal was not to reach consensus between the different groups). For logistical reasons, we conducted individual, semi-structured interviews with five neurologists. Because the goal of the focus groups and interviews was to further explore the findings of the surveys, we did not aim for data saturation.

The Value Proposition Canvas, a framework for matching proposed solutions to experienced needs (23), was used to develop the topic guide. Participants were invited to share their views regarding the following general themes: (1) goals participants wanted to achieve by monitoring symptoms, (2) experienced challenges (“pains”) and benefits (“gains”) of currently used monitoring tools, (3) potential advantages and limitations of wearable sensors, and (4) what the ideal tool to monitor PD in daily life would look like. These themes were discussed within a specific domain of interest, which varied per stakeholder group, and was based on the most important symptoms and motivations identified by the surveys. The full interview guides can be found in Appendix B.

### 2.6. Analysis of focus groups and interviews

All focus groups and interviews were audio recorded and transcribed verbatim. One researcher coded the transcripts using the four themes of the topic guide as pre-defined framework. Within these general themes, thematic analysis based on inductive coding was used. A second, independent researcher commented on the codes to improve their validity. In case of disagreement, the researchers discussed their interpretation of the codes until consensus was reached. Atlas.Ti (version 8.2.29) was used to facilitate the qualitative analysis.

## 3. Results

We will first discuss the results of the online surveys, including the current use of monitoring tools, motivations and barriers for monitoring PD, the most important PD aspects to monitor, and the expected benefits of wearable sensors for monitoring PD. Then we will zoom into different promising contexts for using wearables sensors for each group, discussing the theme’s emerging from the analysis of the focus groups and interviews. Finally, we present expected barriers of wearable sensors, identified in the surveys, focus groups and interviews combined.

The online surveys were completed by 429 PD patients (response rate 60%), 86 physiotherapists (response rate 55%), 55 nurses (response rate 34%) and 25 neurologists (response rate 29%). The background characteristics of the included PD patients are shown in Table 1. From the participating healthcare providers, 96% of the neurologists, 94% of the nurses and 78% of the physiotherapists treated at least 10 individual PD patients per year, most often more than 15 PD patients. The remaining healthcare providers, except for one nurse, treated at least five individual PD patients annually.

### 3.1. Use of monitoring tools (survey)

Approximately one third of the patients had tracked their PD symptoms during the previous year, with no differences between early PD (33, 95% CI: 27–40%) and late PD (34, 95% CI: 28–41%). Most healthcare providers used self-collected information from patients; almost all specialized nurses (94%) recommended at least some of their patients to record the course of symptoms, versus 80% of physiotherapists, and 68% of neurologists. Various modalities of paper diaries were the most frequently used tools among all patient and healthcare provider groups (range: 62–96%). Common examples included free notes, on/off state diaries and falls diaries. The use of digital tools was less prevalent; 14% of patients who monitored their PD used a website [most often the “Parkinson’s Well-Being Map” (24)], 12% used a smartphone or tablet (e.g., digital notes or apps for tracking physical activity), and only 4% of all patients had used a monitoring device or sensor [e.g., Parkinson KinetiGraph (25) or activity tracker] to monitor their PD during the previous year. Differences in tracking tools between early and late PD were negligible. The use of digital tools among healthcare providers was more prevalent: 24% of the neurologists, 23% of the nurses, and 10% of the physiotherapists who recommended their patients to keep track of their symptoms, had already used a wearable sensor in their clinical practice (e.g., Parkinson KinetiGraph or activity tracker). Moreover, 42% of nurses, 24% of neurologists, and 16% of physiotherapists recommended a symptom tracking website such as the “Parkinson’s Well-Being Map.”

### 3.2. Motivations and barriers for self-monitoring (survey)

**Figure 1:**
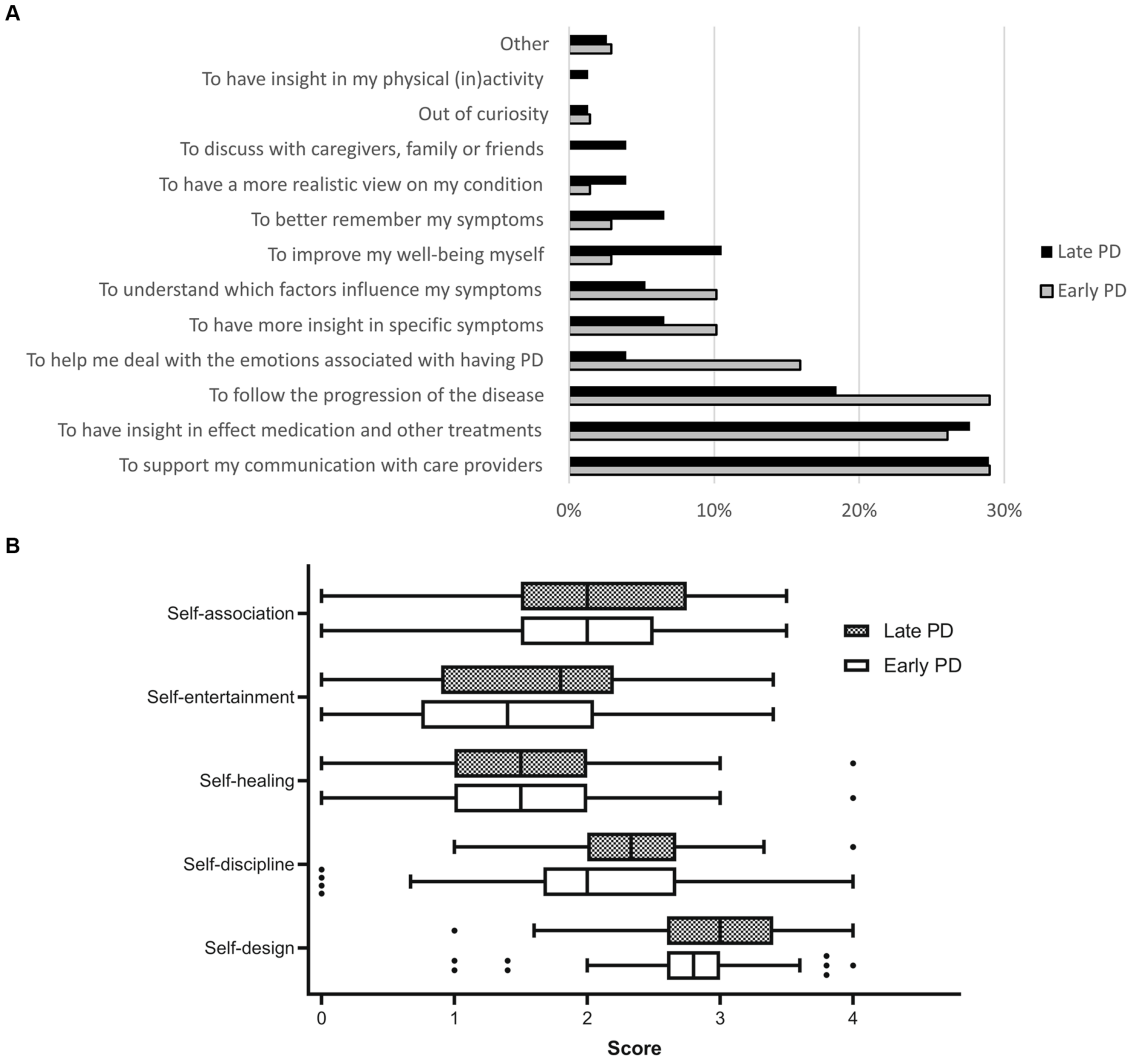
**(A)** Motivations for self-monitoring PD among early PD (*n* = 69) and late PD patients (*n* = 76) who had tracked their PD symptoms during the previous year. Presented categories are based on thematic analysis of open-ended responses to the online patient survey. **(B)** Motivations for self-monitoring among early PD (*n* = 67, 2 missing values) and late PD patients (*n* = 69, 7 missing values) who kept track of their PD symptoms during the previous year, based on five factors of the motivations for self-tracking scale. We show the distribution (median, 25th percentile, 75th percentile and range) of each patient’s average score of all relevant items (0: “disagree strongly,” 4: “agree strongly”; all items were phrased positively).

Among patients who tracked their PD symptoms during the previous year (*n* = 145), we identified various themes describing their motivations to do so (Figure 1). To support the communication with healthcare providers was frequently mentioned by both early and late PD patients. One patient wrote: *“I do it to have an overview of the increase/decrease of complaints for the neurologist and Parkinson nurse.”* Obtaining insights in the effect of medication and other treatments was another important motivation for many patients: *“To gain insight into the efficacy of medications! Especially the variation between on and off moments was difficult to measure!,”* and *“I have kept track of relevant items since the start, now 13 years. Therefore I can see the influence of actions taken.”* More prevalent motivations in the early PD group were (1) following the disease progression over longer time periods (*“To better interpret any decline over a longer time period, and use this to have a potential prognosis, to be able to anticipate on supportive measures”*), and (2) dealing with the emotions associated with having PD (*“To keep having control on the disease, and to deal with it as well as possible”*). Motivations mentioned more often by the late PD group were (1) to better remember symptoms (*“because you cannot remember the many complaints that you come across during the day”*), and (2) to be able to undertake actions yourself to improve your well-being (*“The goal was to split my day into energy blocks, so I can do the most difficult activities during the hours with the most energy,”* and *“to limit the use of medications as much as possible”*).

**Figure 2:**
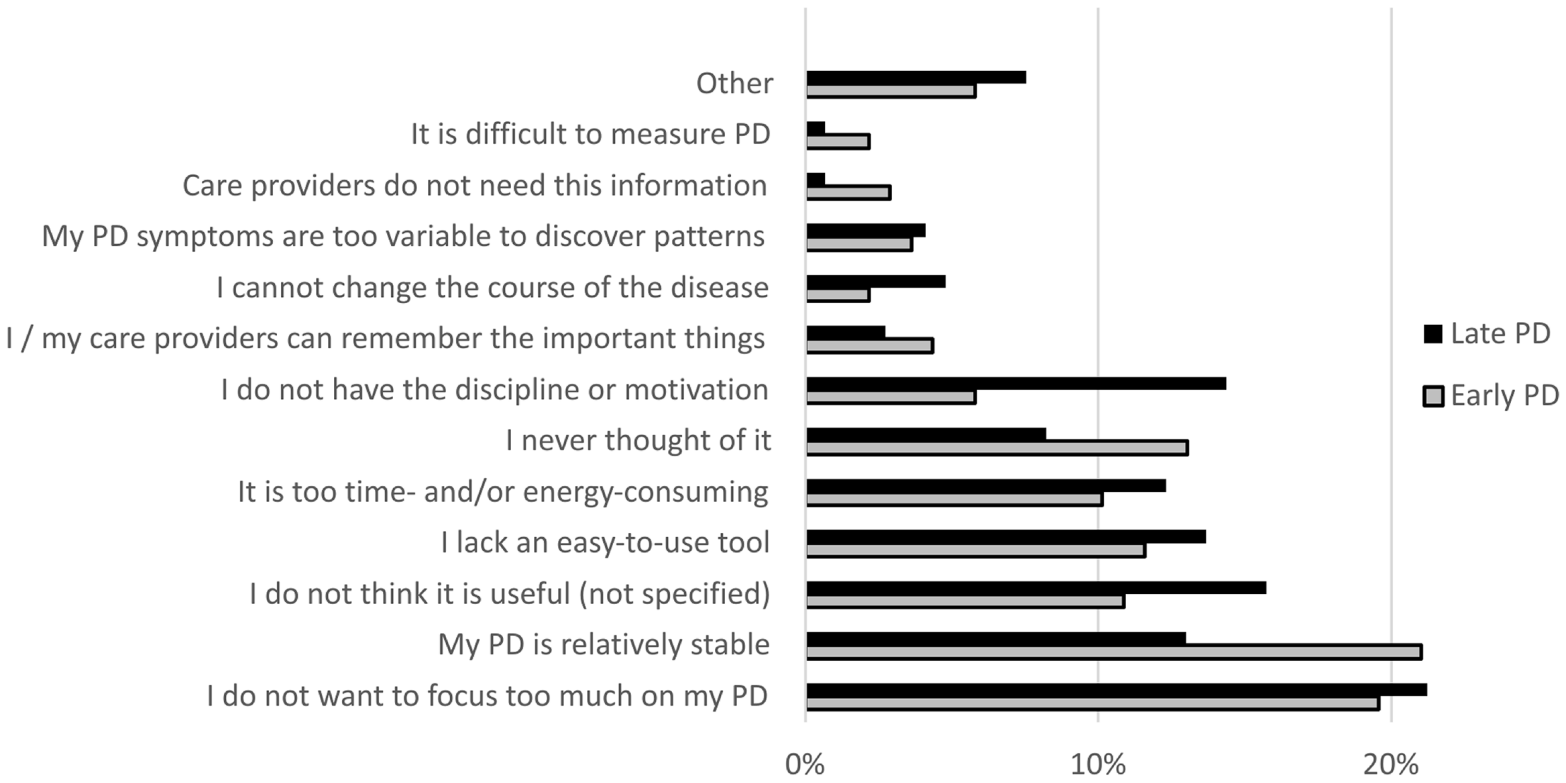
Barriers to self-monitoring PD among early PD (*n* = 138) and late PD patients (*n* = 146) who have not tracked the course of their disease in the last year. Presented categories are based on thematic analysis of responses to open-ended questions in the online patient survey.

On the “motivations for self-tracking” scale, both early and late PD patients scored highest on the self-design dimension (the possibilities of self-optimization), whereas self-healing (independence of traditional medical treatment) and self-entertainment (the pleasure-bringing aspects) were the least important motivations at the group level. On most dimensions, considerable variation was observed between patients (Figure 2).

**Figure 3:**
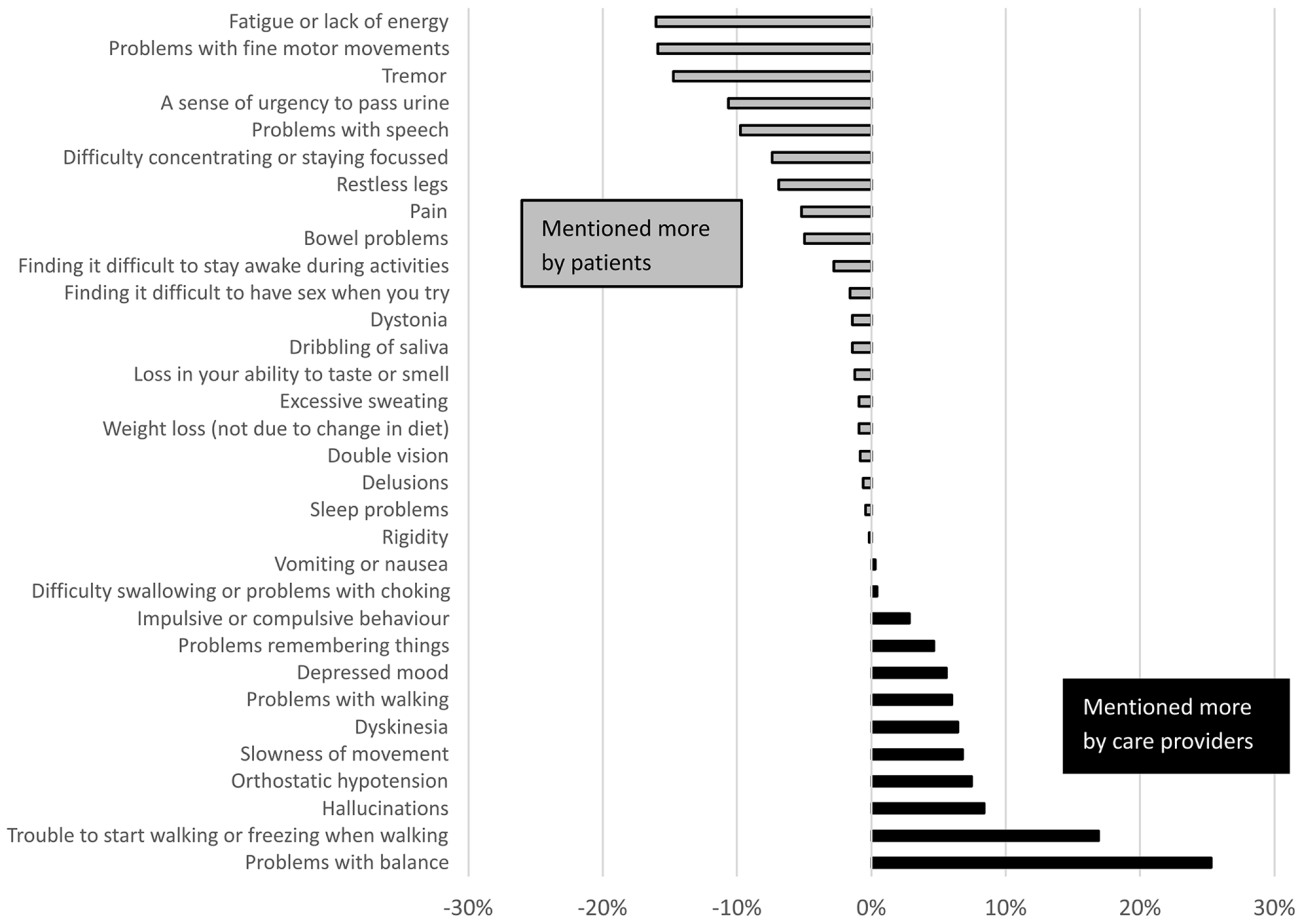
Differences between PD patients and healthcare providers in how frequently symptoms were selected for the three most important symptoms to monitor. Difference is expressed in percent point difference between the average percentage of the patient groups, and the average percentage of the healthcare provider groups.

Among patients who had not tracked their PD symptoms during the previous year (early PD: *n* = 138, late PD: *n* = 146), we identified various themes describing reasons for this (Figure 3). The desire not to focus too much on having PD was an important barrier for many early and late PD patients. Different patients wrote: *“I do not want to become mister Parkinson,” “The tide cannot be turned. I rather look at the positive experiences that I would not have had without Parkinson. Such as new social contacts and friendships through volunteer work, and contact with children and grandchildren as babysit,”* and *“Confrontation with the disease and receiving more info makes me depressed. I put my head in the sand, according to my neurologist much better for me!.”* The most common barrier among early PD patients was the fact that their PD was relatively stable: *“The picture of each day is almost identical. Differences are barely noticeable, also not between medication intakes. I do not notice that the medication wears off, or that I need to take the next dose.”* A common barrier among late PD patients was a lack of discipline or motivation: *“I have tried it once or twice, but I’m not a go-getter, sometimes too tired.”* Some patients thought that keeping track of their PD was too energy-consuming: *“I’ve had Parkinson for almost 14 years now, and my husband died 6 years ago so I’m on my own. I need all my time and energy.”* Other patients missed an easy-to-use self-monitoring tool: *“I do not know a smartphone app,”* and *“Making notes is difficult for me: my hand-writing is very small, typing takes too much time because of repeating keys.”* Last, some patients had never thought about tracking their PD: *“The question only now gives me the idea.”* The fact that some patients mainly experienced “practical” barriers or never thought of tracking their PD, aligns with the fact that two-third (68%) of patients who had not tracked their PD during the previous year indicated to be interested in self-monitoring.

### 3.3. Most important aspects of PD to monitor (survey)

**Figure 4:**
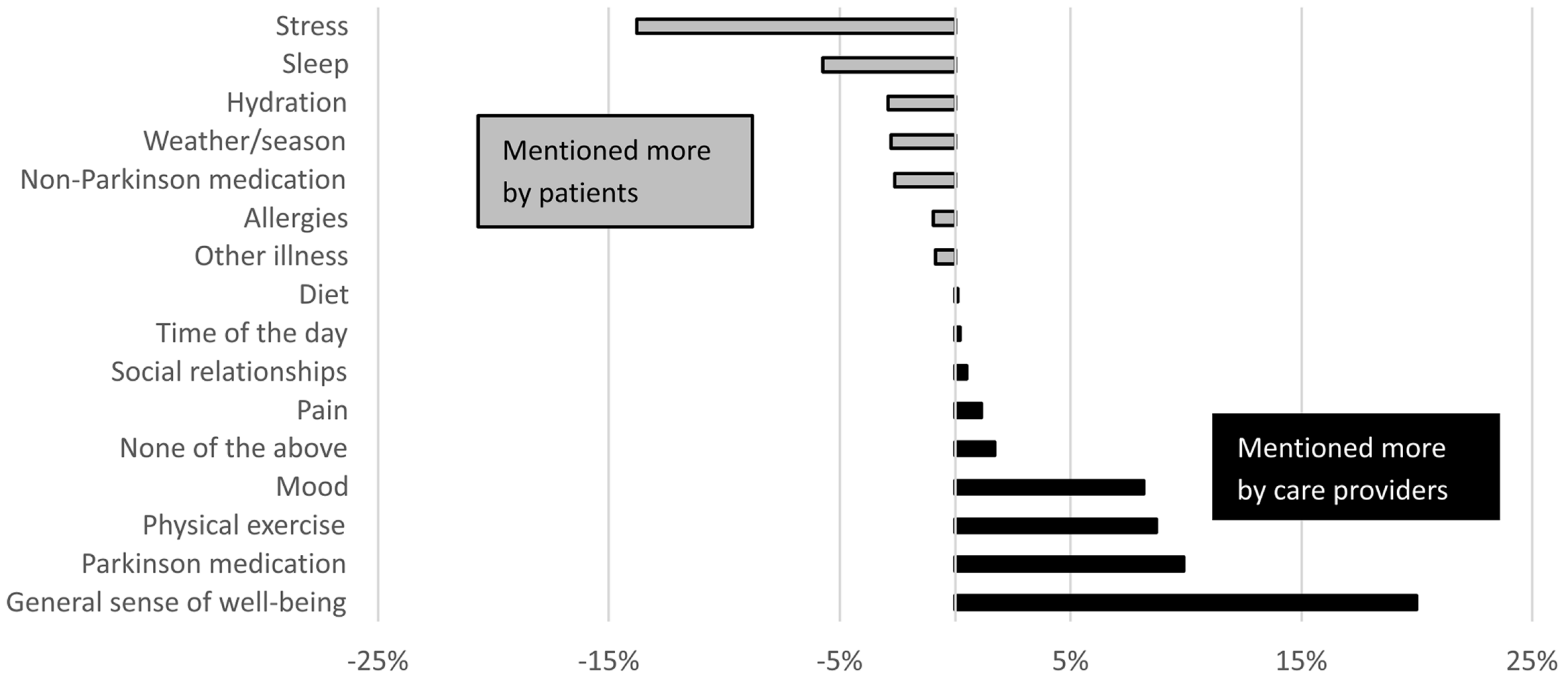
Differences between PD patients and healthcare providers in how frequently factors that influence PD were selected for the three most important aspects to monitor. Differences are expressed as percent point difference between the average percentage of the patient groups, and the average percentage of the care provider groups.

**Figure 5:**
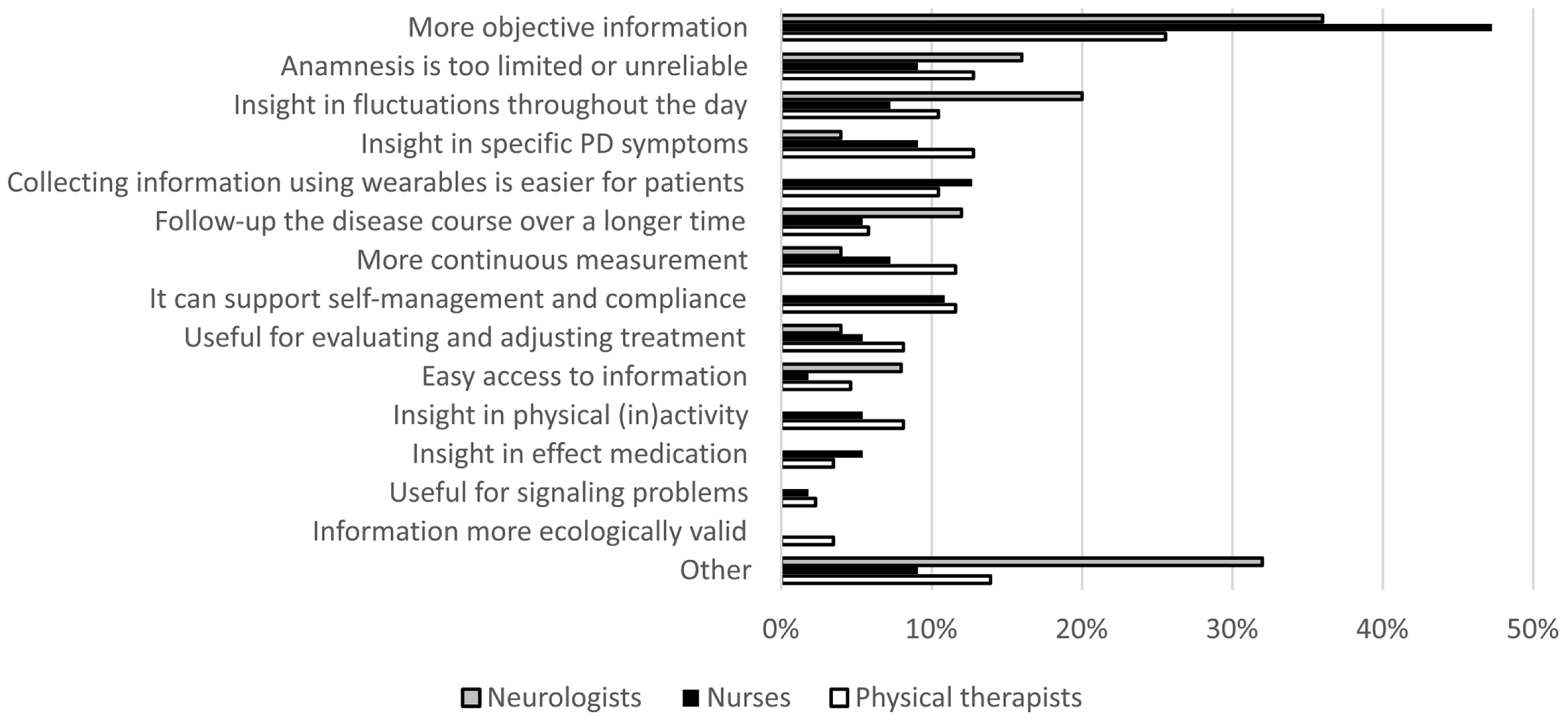
Expected benefits of wearable sensors among neurologists (*n* = 25), nurses (*n* = 55), and physiotherapists (*n* = 86). Presented categories are based on thematic analysis of open-ended responses to the online care provider survey. The prevalence of the “other” category is high in the neurologists group; this is mainly because some neurologists mentioned “better monitoring” as a benefit, but did not specify this further.

The patients who indicated to be interested in monitoring their PD (*n* = 326, 76%), and all healthcare providers were asked for their three most important symptoms and other factors that would merit monitoring in daily life. The five most frequently selected items per group are shown in Table 2. The complete item lists, including all percentages, can be found in Appendix C. The selection made by patients differed from the selection of healthcare providers, which is highlighted in Figures 4, 5. On average, healthcare providers valued information about balance problems and freezing of gait more, whereas patients showed a larger interest in monitoring fatigue, problems with fine motor movements and tremor. Regarding factors that influence symptoms, patients showed more interest in the effects of stress, whereas healthcare providers were relatively interested in monitoring the general well-being of patients. The selection of symptoms by patients was largely explained by how burdensome (early PD: *ρ* = 0.95, late PD: *ρ* = 0.96) and how strongly fluctuating symptoms were (early PD: *ρ* = 0.96, late PD: *ρ* = 0.95). Healthcare providers mentioned several considerations for their selection, including (1) whether they expected that the symptom had a high impact on the patient’s quality of life and/or daily life functioning, (2) whether they could effectively treat the symptom, and (3) whether there is a “knowledge gap,” for example because there is a need for frequent information (e.g., for managing response fluctuations), or because the reliability of in-clinic anamnesis is limited (e.g., for managing falls). Some healthcare providers also mentioned increasing the patients’ self-awareness of symptoms as a motivation for their selection.

**Table 2:** Most frequently mentioned symptoms and factors that influence PD by the different stakeholder groups.

	Early PD^**^ (*n* = 165)	Late PD^**^ (*n* = 161)	Physiotherapists (*n* = 86)	PD nurses (*n* = 55)	Neurologist (*n* = 25)
Symptoms					
1st	Tremor	Rigidity	Balance and falls	Balance and falls	Slowness of movement
2nd	Slowness of movement*	Problems with walking	Problems with walking	Slowness of movement	Dyskinesia
3rd	Fatigue*	Tremor*	Freezing of gait	Freezing of gait	Freezing of gait
4th	Rigidity	Balance and falls*	Rigidity	Rigidity	Balance and falls
5th	Problems with fine motor movements	Fatigue	Slowness of movement	Sleep problems	Rigidity
Other factors					
1st	PD medication	PD medication	Physical exercise	PD medication	PD medication
2nd	Physical exercise	Physical exercise	PD medication	Physical exercise	General sense of well-being
3rd	Sleep	Stress	General sense of well-being	General sense of well-being*	Physical exercise
4th	Stress	Sleep	Stress	Mood*	Sleep
5th	Time of the day	Time of the day	Pain	Sleep	Time of the day

*Equal percentages. **Only completed by patients who indicated to be interested in monitoring their PD.

### 3.4. Expected benefits of wearable sensors (surveys)

**Figure 6:**
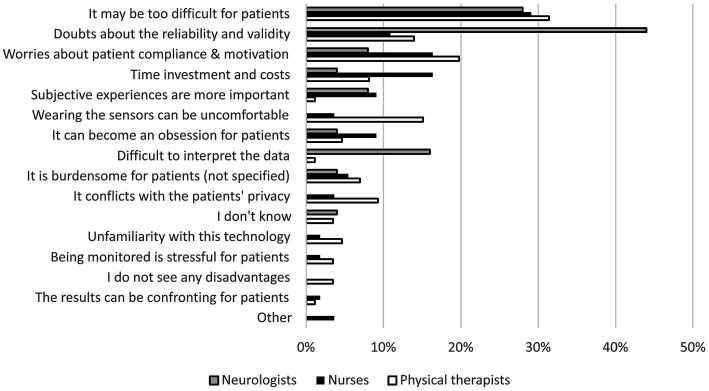
Expected limitations of wearable sensors among neurologists (*n* = 25), nurses (*n* = 55), and physiotherapists (*n* = 86). Presented categories are based on thematic analysis of open-ended responses to the online care provider survey.

Respondents in all healthcare provider groups generally had a positive attitude toward using wearable sensors in PD care; on a seven point Likert scale ranging from 1 (“strongly disagree”) to 7 (“strongly agree”), they responded to the statement “I believe that wearable sensors have the potential to help me monitor my Parkinson patients” with a mean score of 5.5 (neurologists), 5.5 (physiotherapists), and 5.4 (nurses). Figure 6 summarizes the most frequently identified themes of expected benefits. Obtaining more objective measurements was the most frequently mentioned theme across all groups. *“It helps to translate complaints into symptoms,”* according to a neurologist. A nurse wrote: *“Often my patient category cannot clearly put into words what I would like to know. If I could see it myself, that would at least tell me something about what actually happened to someone.”* Different physiotherapists mentioned: *“Patients are inclined to downplay problems, with measurements you obtain a better picture,”* and *“It adds objectivity to my own observations and the responses of the patient.”*

In addition, different nurses and physiotherapists emphasized that wearable sensors could make it easier for patients to track their disease: *“If they are unobtrusive for the patient, it hardly affects their activities and thinking,”* and *“Then the patients do not need to actively make notes.”* Nurses and physiotherapists also saw opportunities for wearable sensors to support self-management and treatment compliance. Different nurses wrote: *“People obtain more insight into the course of their disease, and receive guidance for self-management,”* and *“It can motivate patients, increase their involvement, and make things more insightful for patients themselves.”* Physiotherapists mentioned: *“It can help patients to see for themselves what they can and cannot do,”* and *“It provides people with feedback. And that could well be very different from how people currently see things.”*

For neurologists, one of the main expected benefits of wearable sensors was obtaining more detailed measurements of symptom fluctuations throughout the day: *“It can help to obtain better insights into the level of functioning, and fluctuations in time.”*

### 3.5. Further exploration of each group’s main interest (focus groups and interviews)

Based on the most important symptoms and motivations identified by the surveys, the domains of interest for the focus groups and interviews were chosen: the physiotherapists (*n* = 5) elaborated on the management of balance problems and falls, the neurologists (*n* = 5) on the management of response fluctuations, the nurses (*n* = 6) on supporting self-management, and the patients on communication with their healthcare providers (*n* = 14, divided into two groups). Below we describe the main findings related to the experienced challenges (“pains”) of currently used monitoring tools and the potential advantages of wearable sensors (all identified themes within the Value Proposition Canvas can be found in Appendix D).

#### 3.5.1. Physiotherapists’ view: improved fall risk monitoring

Three main “pains” were identified in the current treatment of falls and balance problems: (1) it is difficult to find out what precedes fall incidents in the patient’s daily life, especially in patients with cognitive impairments or without a partner: “*Often patients say: all of a sudden I was lying on the floor,”* (2) the in-clinic performance measured with standardized assessments (such as the mini-BEST) does not fully explain why some patients fall frequently and others do not*: “On the balance board they perform quite well, their strength is rather good, their catch response is good, and still they fall 3 times per week. That is quite frustrating,”* and (3) patients tend to forget applying strategies to prevent falling that the therapists teach them in-clinic: *“When I am standing next to them, they perform things very differently. Last time a patient stepped over everything, but when I walked next to him he carefully walks around obstacles and takes the right path.”*

According to the physiotherapists, wearable sensors worn in daily life could help by increasing the self-awareness of the fall frequency and situations with a high fall-risk, as a starting point for therapy: *“If I as a therapist see someone who has reached his limit and I can talk with the patient about that: on these moments in your daily life you take risks.”* Objective data about what precedes a fall incident could help to identify different “fall profiles” that would enable more targeted therapy. One physiotherapist said: *“Together with the tests with patients we already perform, I would like to make a sort of risk analysis, of which factors are causing the fall. Then, based on the patient profiles, I can give my patients tailored verbal instructions. That would be a good example of using wearable sensors and connecting data.”* Sensor devices could also be used to coach patients, for example by detecting situations with high fall risk and providing warnings or reminders to apply the right movement strategy: *“If it has to do with selective attention and the sensor can recognize the movements before a fall occurs, then a warning signal may make someone alert so that he makes the right decision,”* or coach patients to maintain a healthy gait pattern: *“I can imagine that you have a sensor that in some situations sends a verbal message: pay attention, big steps, keep on stepping.”* In addition, the therapists saw benefits for stimulating patients to do balance exercises: *“It think it can be motivating for balance exercises which they need to perform at home, and they receive a signal when it goes well.”*

#### 3.5.2. Neurologists’ view: better management of response fluctuations

Three main “pains” regarding managing response fluctuations were identified. First, neurologists often find it difficult to understand the daily patterns of response fluctuations based on the patient’s story. One neurologist said: *“Often patients say I am not doing well doctor, and then you need to figure out why: is it because of motor problems? And if this is the case, is it rigidity, dyskinesia or tremor?.”* Self-reported on–off diaries are often not very helpful*: “The patients who can accurately describe it are also capable of filling out such a dairy, it’s mostly the patients who find it hard to explain it, they also have problems completing the on–off diaries.”* A complicating factor is that some patients find it difficult to distinguish between tremor and dyskinesias. Second, it is difficult to rely on in-clinic observations: *“The situation here in the consultation room is always different than at home, so you rely on what patients experience at home.”* Third, it can be challenging to determine who is eligible for advanced therapies: *“I often refer them to Nijmegen for that, and then they also find it difficult. It is very difficult to get an accurate picture. Now patients are often admitted to the hospital for that.”*

Objectively quantifying response fluctuations in real-life could help neurologists to find the right medication dosage more efficiently: *“That you can give the right medication dosage more quickly, that you can go through the process of adjusting the medication schedule faster,”* and *“Then you can see at a glance whether the patient responds to the treatment or not.”* Specifically, it could be helpful to find out whether motor symptoms are the main problem: *“It would give a nice impression of how patients are doing in terms of motor symptoms, and if you see that patients are doing well motorically, then you know something else is going on. I think that that is a huge benefit.”* In addition, it might help to identify patients who would benefit from advanced therapy: *“That you can identify the phase when the medication really does not work anymore earlier. And that you can use this to refer patients for advanced therapies in an earlier stage.”* The ability to provide care proactively was also seen as an important benefit: *“I think that you can also use it to signal problems in an early stage, …, that you receive an early signal when a patient falls outside a certain range, when we should schedule an earlier check-up, or when the GP or local Parkinson nurse should have a look, to prevent certain problems, for example falls, confusion, or delirium.”* In addition to these forms of decision support, neurologists also mentioned benefits for their communication with patients. Wearable sensors may help to increase the self-awareness of patients: *“You may give a patient more insights into his own functioning if you can monitor him for a longer time than when you briefly discuss things in the consultation room.”* It may also help to focus the conversation: *“It makes the conversation much more concrete, because you can focus very timely on the current problems of a patient.”*

#### 3.5.3. Parkinson nurses’ view: educate patients and stimulate self-management

Three main “pains” were identified among Parkinson nurses. First, some patients find it difficult to reflect on and understand their own symptoms. Different nurses mentioned: *“If we ask very specifically, what do you experience and how does it present itself, patients often find it difficult to pinpoint,”* and *“I saw a patient and when she goes into an off state, she really panics. She does not recognize the off phenomenon yet, which makes her hyperventilate.”* In addition, for some patients understanding the difference between tremor and dyskinesias is difficult: *“If you are dyskinetic, and you take extra dopamine, it only becomes worse.”* Some patients also have the tendency to underestimate their sleep duration: *“Sometimes it is the experience of a patient that he sleeps for only 3 h, while it appears to be different. I always find this a difficult point to discuss.”* Second, the nurses emphasized that completing diaries is often burdensome for patients: *“The partner or someone else constantly looks over your shoulder and says: “you still need to fill it in,” and that drives some people crazy.”* For some PD patients, this may even become an obsession: “*The people who become very rigid in their behavior because of their PD and who want to rationalize everything in numbers, they sometimes show up with whole packages of information and then the partners tell us: “we cannot leave the house without taking pen and paper with us,” or they bring extensive tables and graphs. That is real obsessive behavior.”* This makes some nurses also question how representative the diaries are: *“I wonder, how realistic is it, because the stress that comes with filling in the diaries also makes symptoms different than they normally are.”* Third, some patients in the early stages struggle with accepting the diagnosis: *“Patients visit the neurologist and he says: “you have Parkinson’s.” Then people think, that cannot be true, nothing has been done. That’s why many patients keep on wondering: is the diagnosis true, because we cannot do imaging.”*

The nurses thought that wearable sensors could help to increase the patients’ self-awareness. One nurse mentioned: *“I could mention tens of patients of whom I think: yes, that would actually be nice to make it insightful: what really happened and discuss that together.”* About the patient who panicked during off phases, the nurse said: *“It could help her if she could say: last week I had such an attack, and that we can then discuss: it really looks like an off phase, which is confirmed in a graph.”* Self-awareness about the cause of falls may help the patient to help himself: *“Then you could say: you should have stood up less quickly. If someone has a gap in his memory and does not know it anymore, they also cannot help themselves.”* Nurses also emphasized that wearable sensors could make it easier for patients to tell their story: *“They already have less dopamine, so a conversation costs a lot of energy. I can imagine that it helps if you already have some numbers and the patient does not have to tell the whole story.”* In addition, they saw a role for wearable sensor to activate patients: *“If a sensor gives certain stimuli for loss of initiative, that could unburden caregivers a little because he does not continuously have to stimulate the partner and be in the caregiver role, and can be more of a partner.”* Last, having objective measurements could help with accepting the diagnosis: *“Often patients feel like: is the diagnosis true, because I cannot confirm it with imaging. This* (i.e.*, feedback from wearable sensors*) *is something that patients can really see, something that is being measured.”*

#### 3.5.4. Patients’ view: communication with healthcare providers

How patients communicated about their symptoms with healthcare providers varied per individual. Some patients already made notes about the most important changes or questions before meeting with their healthcare providers: *“Before I visit my neurologist, I always make one sheet with what I want to say, so I do not forget anything.”* Some patients regularly used an online questionnaire (“Parkinson Monitor,” developed by the Dutch Parkinson Association) to identify the biggest changes in their symptoms compared to the last appointment. For some patients, the partner’s support during consultations was very important: *“I have a very good partner who joins me with a memory like an elephant.”* Others did not feel the need to track their Parkinson symptoms, either because their situation was relatively stable or because they felt like their disease course was too unpredictable to identify useful patterns: *“I started with it, only for me every day is different. There is no logic to it, so at a certain point I felt like: what’s the use of keeping track of it.”* Identified facilitators (“gains”) for communicating with healthcare providers about symptoms included (1) an open attitude to using self-collected information: *“My neurologist says: “I am happy you brought a form, because I am depending on you.” She can only help if I say something,”* and (2) whether their healthcare providers were easy to approach: *“We have the best feeling with the Parkinson nurse. She maybe does not know 100% about my patient record, but she does have eye for the social aspects and thinks with you if you say: “I went on a holiday and it was so nice.” It feels closer.”*

Three main “pains” were identified with respect to the communication about symptoms with care providers. First, some patients thought it was difficult to collect reliable information to share. The Parkinson Monitor was considered as too subjective: *“I tried it and I thought it was much too subjective. You need to give a number, and if I selected a 6 last time, I do not remember why I choose a 6 then.”* In addition, patients who wanted to try a smartphone applications, found it difficult to know which one was reliable: *“If I look for Parkinson’s in the app store, there are so many applications. I do not know which ones are any good.”* Second, some patients thought it was burdensome to self-track their PD, either because it required a lot of time or because they did not want to focus too much on their PD: *“I am very eager to learn, so I thought I want to know everything about the disease. It made me very sad day by day, I did not sleep anymore, and I became depressed.”* Third, some patients had the impression that their care providers are not open for self-collected information: *“The neurologists inspects how I walk when I come in and looks at my facial expression and says: you are doing well. That is what he relies on.”* Another patient mentioned: *“I know the Parkinson Monitor, but the neurologist thinks it’s nonsense and he does not cooperate, so then there is no point.”* Patients thought that the limited time for consultations was an important factor in this.

Patients who were interested in symptom monitoring, agreed that wearable sensors could provide more objective information, which would be useful to share and discuss with healthcare providers. First, patient thought that it could help to find the right treatment, for example by adjusting the medication schedule more quickly: *“Imagine that you agree during a consultation that the medication needs to be increased because you are too passive. Then you can see during the next 2 weeks: has it changed or not. Now you wait for 3 months until you go back.”* Similarly, one patient thought that it could help with adjusting the intraduodenal levodopa infusion: *“If you get it, you need to go for a week to the hospital to see: how are the settings. That should work much better with such a device.”* Patients also saw benefits for non-pharmacological interventions: *“With walking, sometimes it goes smoothly and sometimes I think: “flap, flap, flap.” So then I think: we should analyze the movements with sensors, and then get an advice which exercises you should do.”* Second, some patients thought that it could help healthcare providers to proactively signal changes that need attention: *“Then they might be able to see in the data in an earlier stage: something is not going well, maybe we should schedule a check-up earlier.”* Third, sensors could facilitate the communication during consultations: *“I think that they are even more prepared for what happened, that they can read in before the appointment. Then you do not need to mention everything, because everyone is up-to-date.”*

### 3.6. Expected barriers and contextual considerations for use of wearable sensors (mixed methods)

**Figure 7:**
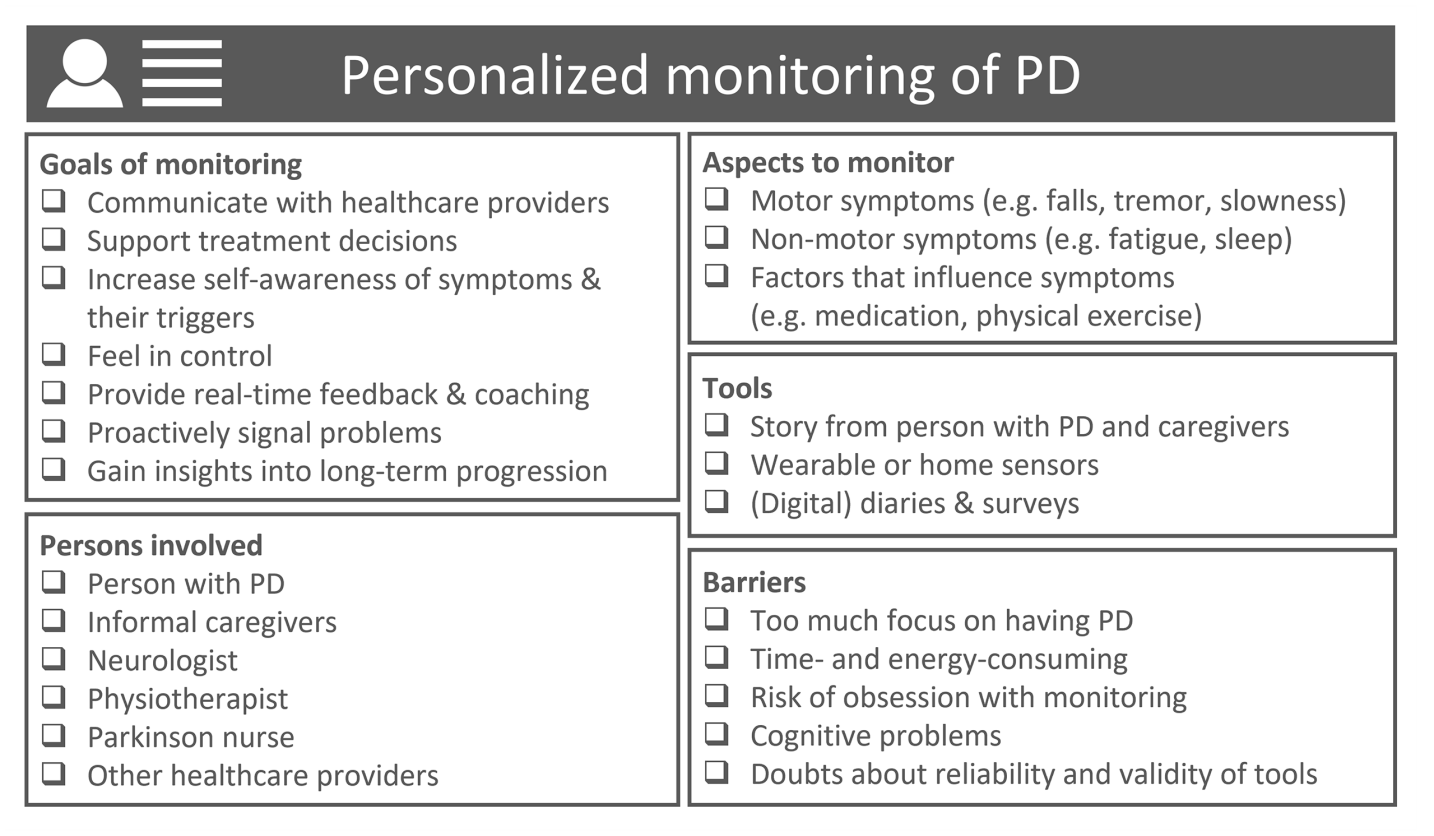
Visualization of personalized monitoring of PD, providing a non-exhaustive overview of aspects that should be considered when developing PD monitoring solutions to address specific needs of specific target groups of patients and healthcare providers. Categories are inspired by the results from the surveys, focus groups and interviews.

The most frequently identified barriers to using wearable sensors among healthcare providers are shown in Figure 7 (the percentages were based on the survey, whereas the illustrative quotes below were based on the survey, focus groups and interviews combined). Concerns about the usability of wearable sensors, in particular for the PD population, was a common theme. Some healthcare providers commented on the motor skills required for using the devices: *“It may be difficult for patients to put the sensors on and take them off,”* one physiotherapist wrote, and one nurse mentioned: *“Patients often have problems with fine motor skills.”* Cognitive problems were a common concern as well: *“Patients may forget that we agreed to wear the device, or how to use the device,”* according to one of the nurses. A physiotherapist mentioned: *“Some patients with Parkinson’s disease are not teachable anymore.”*

Both patients and healthcare providers, neurologists in particular, emphasized the importance of reliable and well-validated sensor-based outcomes: *“It is important whether it actually reflects the condition of the patient”* (neurologist), and *“A sensor only gives objective data if it is really good. A system of a few years ago could not detect biking, then it does not work, then it is not a complete, objective picture”* (patient). Some healthcare providers would only trust the measurements if recognized by the patient: *“I would trust it if you look at it together with the patient and he says multiple times: yes that is true, I also experience it that way”* (nurse). A neurologist noted that he needs to able to rely on measurements, also when findings are unexpected: “*On the other hand, if it completely matches one-on-one with what I already thought myself, then the added value is of course zero”* (neurologist). Some healthcare providers emphasized the importance of transparency: *“I think it’s difficult if you cannot look under the hood. If it does not seem to match with what you think about this patient, you cannot really see why it does not match.”* The scope of what could be measured with wearables was also a concern: *“Only limited measurements are possible: for example one arm or one symptom”* (neurologist), and *“We talk a lot about cognitive problems. The sensors measure movements, so there is already some friction”* (physiotherapist).

Another common theme was the compliance with using wearable sensors: “*The devices will not always be used by the patient, so information will not be complete, which can make patients very nervous”* (nurse), and *“Patients may take them off and forget to put them on again”* (physiotherapist). Some healthcare providers thought that the patients’ motivation to wear the devices is an important hurdle: *“It asks a lot of discipline from patients”* (physiotherapist), and *“Patients must benefit from it themselves”* (neurologist).

Healthcare providers were also concerned about the time investment and costs. A physiotherapist wrote: *“Reading out the sensors requires extra time which is not there, or it comes at the expense of treatment time,”* and a neurologist expressed the concern that it may raise more questions: *“Then they tell me in the app I see this and that. Cannot you increase the medication even more?”* (neurologist). In addition, healthcare providers stressed the importance of the subjective experiences of patients: *“You do not treat graphs, but patients. So how the patient eventually experiences it remains most important”* (nurse), and *“It is very important that patients can indicate via a simple button how they feel from time to time, for example whether they are feeling comfortable, miserable, stressed,* etc. *That is important for the interpretation of the data. The combination of subjective and objective data is important”* (neurologist). Last, some participants mentioned the risk that self-monitoring can become an obsession, in particular for patients with PD: *“A disadvantage could be that you let your life be ruled by the sensors. I would not be happy with that”* (patient).

In the focus groups, additional themes regarding the design and implementation of wearable sensors were identified (“the ideal tool” in the Value Proposition Canvas). Below we highlight highly prevalent themes; for all identified themes and illustrative quotations we refer to Appendix D.

#### 3.6.1. Use for specific indications

Healthcare providers mainly saw benefits for specific target groups: *“On the long term, I think we will mainly use it for the vulnerable patient with little informal care, or with cognitive problems when you think, I cannot get a good impression of how the patient functions at home, and that you have doubts about the medication or activities of such a patient”* (neurologist), and *“It is particularly important for patients living by themselves and in nursing homes: here we often do not have a clear picture of the patients’ functioning”* (nurse). Both patients and healthcare providers also mentioned that there need to be specific goals and the use of wearable sensors should not be standard: *“I find everything that’s standard a bit tricky. In contrast, we aim for personalized care, and if you say: we will do certain things as standard when they have Parkinson’s for 5 years, I would hate that.”* A patient mentioned: *“Yes, the data are very nice, but you can quickly drown in all the information, so you need to have a thread or a goal. I have a goal: stay active.”*

#### 3.6.2. Active versus passive monitoring

Because of the need to obtain (continuous) insights into how the patients move in real-life, healthcare providers generally preferred passive registrations in the background over performing active tasks: *“Otherwise patients focus on the exercise and not on the environment and why he stands up. He stands up for a reason, not to do that test, but he needs to go to the toilet. You can measure that in a very natural way”* (physiotherapist), and *“At the end of the day you would still do artificial measurements then. That’s not what it’s really about. They are still snapshots”* (neurologist). Some patients were also more enthusiastic about passive monitoring: *“I think you should not do extra movements for it. It needs to measure automatically, I should not have to say: now you measure me and now you do not.”* Physiotherapists were interested in active tasks if these also served as an exercise: *“It can be valuable if you say: I think this is an important exercise for this patient to repeat often.”* Patient expressed the desire that the schedule of tasks should be personalized and that sensors should sense when the patient is not available: *“A smart sensor knows from your movements that you are in a car, so it would be smart if it does not send you an alert then.”* Nevertheless, some patients doubted whether they had the discipline to do repetitive tasks: *“In every test that I participated in I always needed to do the same thing. Counting back from 100 with steps of 7, always the same test. Once in a while you should think about something else.”* Some healthcare providers thought it would be valuable to combine the sensor data with subjective self-reports: *“It is very important that patients can use a simple button from time to time to indicate how they feel: for example if they feel good, miserable, stressed,* etc. *That would really help interpreting the data. The combination of subjective and objective data is important”* (neurologist).

#### 3.6.3. Privacy

Healthcare providers were generally more concerned about privacy than the patients themselves: *“Well, it feels a bit like big brother is watching you, I think a patient may experience that as unpleasant. It depends a little bit on how you measure it. You are already a patient and if you are also being monitored continuously, I can imagine if a patient would not like that”* (neurologist), and *“It should not work like: let us have a look at how mister X is doing tonight, whether he is sitting on the couch or he is doing his exercises. That is a bridge too far for me”* (physiotherapist). Patients were generally very open to share information with their healthcare providers, and mainly emphasized the positive aspects of data being available: *“Imagine that something happens and I have a question, then he can have a look at how I am doing. It is available. It is not a bad thing if it is available for people you trust.”* Patients did emphasize they would like to have control on who has access to the data: *“If I can say who and when, then I think it’s fine. My physiotherapist can see it for sure, because I see him every 14 days, maybe someone else not.”* Some patients mentioned that data should not be shared with insurance companies. According to healthcare providers, it is important to give detailed information to PD patients and their caregivers about monitoring tools, by whom and how they are used, and to obtain informed consent. The nurses emphasized that respect for the patient’s autonomy is essential, and it needs to be evaluated in each individual case if and what kind of home monitoring is useful and desirable: in some cases, directly transferring the data to healthcare providers could help signaling problems and be experienced as supportive, whereas in other cases, this might be experienced as not respecting the patient’s privacy.

## 4. Discussion

### 4.1. Main findings

This mixed methods study provides detailed information about the perspectives of patients, physiotherapists, Parkinson nurses and neurologists on monitoring PD in daily life. One third of the patients had self-monitored their PD symptoms in the past year, most commonly using a paper diary. Key motivations for monitoring among patients are sharing information with healthcare providers, obtaining insight into the effect of medication and other treatments, and following the long-term disease progression. Key barriers are not wanting to focus too much on having PD, symptoms being relatively stable, and lacking an easy-to-use tool. Symptoms of interest differed between patients and healthcare providers; patients gave a higher priority to fatigue, problems with fine motor movements and tremor, whereas healthcare providers more frequently prioritized balance, freezing and hallucinations. PD patients as well as healthcare providers were in general positive about using wearable sensors to improve PD care and self-management, although the specific context and expected benefits varied considerably between the different stakeholders. For each group we provide further ideas about one promising context where wearable sensors could add value: treatment of balance and falls (physiotherapists), self-management and patient education (Parkinson nurses), treatment of response fluctuations (neurologists), and communication with healthcare providers (patients). Last, we discuss barriers for the use of wearable sensors as identified by the different groups (e.g., questions about usability, reliability, and compliance), as well as suggestions for the design and implementation of wearable sensors.

### 4.2. Toward personalized monitoring

We observed a large heterogeneity among PD patients and healthcare providers regarding their views on monitoring PD, which underlines the need for solutions tailored to specific contexts. The observed heterogeneity is reflected in multiple ways. First, although patients and healthcare providers share interest in the classical motor symptoms of PD, which is in line with earlier studies (11, 14), interesting differences also appeared. Fatigue, problems with fine motor movements, tremor, and stress were mentioned more commonly by patients, whereas healthcare providers gave a higher priority to monitoring balance, freezing of gait, and general sense of well-being. On the one hand, these differences could encourage professionals to pay more attention to symptoms frequently mentioned by patients, especially since interventions to treat fatigue (26) and stress (27, 28) in PD patients are increasingly available. On the other hand, we need to acknowledge that the perspectives of patients and healthcare providers may be inherently different: patients mainly tend to focus on aspects that are most burdensome for them, whereas the different healthcare providers mainly focus on areas where they can have an impact by providing tailored treatments, and where accurate information to support such treatment decisions is presently missing.

Second, healthcare providers expressed different ideas about how monitoring PD using wearable sensors could contribute to improving PD care. Wearable sensors were not only seen as tools to support treatment decisions and proactively signal problems, but also as tools to educate patients, increase their self-awareness of symptoms and triggers, increase their participation, and support treatment compliance. The latter could be particularly relevant for treatments that require a substantial active contribution from patients such as physiotherapy exercises, where wearable sensors could help by visualizing the achieved progress, or even by providing real-time feedback about the execution of exercises (29). In addition, wearable sensors could assist in the challenging transition from practicing movement strategies in a supervised setting to correctly applying them in daily life, by providing real-time feedback in daily life (30, 31).

Third, patients expressed different motivations for self-monitoring their PD. In addition to sharing information with healthcare providers, patients also saw added value of self-monitoring independent of their relationship with healthcare providers. Many patients expressed an interest in gaining more insight themselves into the course of their symptoms and into the effect of medication or other interventions. An important perk of this was the opportunity to feel more in control, and being able to optimize aspects of their lives themselves (self-design), which is in line with findings of Riggare et al. (12). Also, some patients found it useful to self-monitor symptoms to communicate about their PD with family and friends (self-association). Some patients expressed the hope that wearable sensors could be used to coach them, for example to maintain a healthy gait pattern (self-discipline). As such, self-monitoring using wearable sensors offers various opportunities to support self-management when properly integrated into treatment programs (32). However, the added value of self-monitoring will likely depend on whether it fits with the patient’s personal coping strategies, and it is important to find a balance between the benefits and burdens (e.g., the required time and energy, and the fact that self-monitoring can be confrontational) (12, 33).

Taken together, we conclude that it is unrealistic that a one-size-fits all monitoring solution will be able to address the different needs of PD patients and healthcare providers involved in PD care. Instead, we believe that different PD monitoring solutions should be designed to address specific needs experienced by specific target groups of patients and healthcare providers (6), with close involvement of the users in all phases of the product’s design (15, 34).

### 4.3. Impact on interaction between patients and healthcare providers

Patients and healthcare providers expected that the use of sensor-based monitoring tools will impact their interaction. On the positive side, being able to discuss measurements together could serve as a memory aid, trigger patients to share their experiences, and help to focus the conversation. Jointly discussing measurements was also seen as a way to increase the patients’ self-awareness. In addition, by reducing the dependence on in-clinic observations to evaluate the severity of symptoms, wearable sensors provide opportunities for telemedicine (35), in which the COVID-19 crisis triggered a revived interest (36).

However, the use of sensor-based monitoring tools also comes with challenges for the communication between patients and healthcare providers. Focusing too much on numbers was identified as a potential risk. Healthcare providers agreed that the subjective experiences of the patient remain vital to guide treatment decisions, as wearable sensors cannot measure the limitations experienced by the patient. This stresses the importance of providing patients ample opportunity to comment on the measurements, and only act upon them if jointly agreeing on the conclusions, which is in line with findings of the focus group study of Ozanne et al. (37). Future clinical trials on the effectiveness of specific remote monitoring tools should include more elaborate evaluations of their impact on the relationship and communication between patients and healthcare providers (38).

### 4.4. Uptake of wearable sensors

Despite an increasing availability of sensor-based monitoring tools, both our survey and the survey of Mathur et al. (11) showed that paper diaries are currently the most commonly used tool among patients and healthcare providers. This may be partly explained by the lack of convincing evidence for the benefits of wearable sensors. A few pilot trials using sensor-based remote monitoring systems have demonstrated positive effects on clinical decision-making and motor symptoms of PD patients (34, 39, 40). However, these studies had different methodological shortcomings (including lack of randomization, small sample size, and no assessment of user experiences), and were conducted by the groups who also developed and commercialized the systems. The field would benefit from independent randomized controlled trials and qualitative process evaluations of mature versions of remote monitoring systems. In addition, systems are often evaluated in broad PD populations. Based on the observed heterogeneity in needs that we identified among patients and healthcare providers, it is unlikely that all PD patients will benefit from such solution. Instead, it would be more appropriate to conduct evaluations in well-defined, specific use cases. Randomized controlled trials such as the ongoing MoMoPa-EC study are an important step in this direction (41). In addition, the focus has been on supporting clinical decisions around the prescription of medication, whereas opportunities also exist for supporting non-pharmacological interventions (e.g., by physiotherapists) and self-management. Finally, given that concerns about the reliability and validity were commonly mentioned as barriers for using sensor-based monitoring tools, building trust in newly developed sensor-based outcomes is essential (42).

### 4.5. Strengths and limitations

This study has a few limitations. First, a relatively small number of patients and healthcare providers participated in the focus groups and interviews. Because we did not aim for data saturation, the identified themes cannot be assumed to be exhaustive, also given the observed large variation in individual perspectives. Instead, our aim was to enrich the findings of the online surveys by further exploring promising contexts where wearable sensors could be of added value. Second, the organization of PD care in some other countries differs from the Netherlands, where, for example, PD patients are often seen by physiotherapists and Parkinson nurses, in addition to neurologists. Therefore, the roles of different healthcare providers should be considered when translating the findings of this study to other countries. At the same time, the Netherlands lends itself well for studying innovations in PD care, because of the nation-wide network of healthcare providers specialized in PD (19). Third, it should be noted that, because patients signed up to participate in a wearable sensor study, our study population may be relatively interested in this topic. We believe this did not affect the generalizability of our findings, as approximately two-third of the participants did not perform self-tracking activities. Finally, although we involved the most frequently involved healthcare providers in PD care, future research may explore the perspectives of other relevant disciplines, such as speech and language therapists, occupational therapists and dietitians (10).

Strengths of this study include the combination of surveys with subsequent focus groups, and the involvement of both patients and different healthcare providers. The alignment of the survey questions allowed for a comparison of perspectives, highlighting interesting differences between the different stakeholder groups. In addition, by not limiting the input of participants to the development of a specific solution, we aimed to identify universal needs, not limited to what is currently technically possible. Finally, we aimed to provide a nuanced view on the potential of new monitoring solutions, by not only focusing on motivations for self-monitoring and expected benefits of wearable sensors, but also on barriers and expected limitations. More insights into the different perspectives on symptom monitoring and wearable sensors of all stakeholders involved will hopefully contribute to the successful design and implementation of PD monitoring solutions.

## Data availability statement

The anonymized raw data supporting the conclusions of this article can be shared by the authors upon request, without undue reservation.

## Ethics statement

The studies involving human participants were reviewed and approved by Commissie Mensgebonden Onderzoek, regio Arnhem-Nijmegen; file number 2015-1776. Participants in the online survey study provided informed e-consent; participants in the focus groups and interviews provided written informed consent.

## Author contributions

LE, BB, and MM were responsible for the conception of the research idea and design of the study. BB and MM obtained the funding for the study. LE and MM wrote the research protocol and obtained approval from the ethical committee. LE developed the surveys and the topic guides for the focus groups and interviews. LE and JP collected the data, performed the analyses, and drafted the initial manuscript. MM and BB helped with interpreting the results, and thoroughly reviewed the manuscript. All authors read and approved the final version of the manuscript.

## Funding

This work was financially supported by the Michael J Fox Foundation (grants #10231 and #020425), UCB Biopharma, Stichting ParkinsonFonds, Netherlands Organization for Scientific Research (grant #91215076), and by the Dutch Ministry of Economic Affairs by means of the PPP Allowance made available by the Top Sector Life Sciences & Health to stimulate public-private partnerships (grant #TKI-LSH-T2016-LSHM15022).

## Conflict of interest

LE, JP, and MM received funding for this work from the Michael J Fox Foundation, UCB Biopharma, Stichting ParkinsonFonds, the Netherlands Organization for Scientific Research, and by the Dutch Ministry of Economic Affairs by means of the PPP Allowance made available by the Top Sector Life Sciences & Health to stimulate public-private partnerships. BB serves as the co-Editor in Chief for the Journal of Parkinson’s disease, serves on the editorial board of Practical Neurology and Digital Biomarkers, has received fees from serving on the scientific advisory board for Biogen and UCB (paid to the Institute, not to BB), has received fees for speaking at conferences from AbbVie, Biogen, UCB, Zambon, Roche, GE Healthcare, and Bial (paid to the Institute, not to BB), and has received research support from the Netherlands Organization for Health Research and Development, the Michael J Fox Foundation, UCB, AbbVie, the Stichting Parkinson Fonds, Hersenstichting Nederland, the Parkinson’s Foundation, Verily Life Sciences, Horizon 2020, the Topsector Life Sciences and Health and the Parkinson Vereniging, outside the submitted work. BB does not hold any stocks or stock options with any companies that are connected to Parkinson’s disease or to any of the topics in this manuscript.

## Publisher’s note

All claims expressed in this article are solely those of the authors and do not necessarily represent those of their affiliated organizations, or those of the publisher, the editors and the reviewers. Any product that may be evaluated in this article, or claim that may be made by its manufacturer, is not guaranteed or endorsed by the publisher.

## Footnotes

^1^www.surveygizmo.com
