# Supplementary material for: Need for personalized monitoring of Parkinson’s disease: the perspectives of patients and specialized healthcare providers
Source: Front Neurol. 2023 May 4;14:1150634. doi: 10.3389/fneur.2023.1150634 (PMC10192863; doi:10.3389/fneur.2023.1150634)
Supplement: Supplementary file 1 [file data_sheet_1.zip › Data Sheet 1 - updated/Appendix A1.pdf]

Appendix A1 - survey patients Dutch

Welkom bij deze online vragenlijst van de ParkinsonThuis studie!

Wat is het doel van deze vragenlijst?
Ons onderzoek richt zich op het ontwikkelen van handige manieren om Parkinson-klachten bij te houden in het dagelijks leven. Uw mening als ervaringsdeskundige is hierbij onmisbaar! Met deze vragenlijst willen we beter begrijpen wat belangrijk is voor mensen met de ziekte van Parkinson. Vragen die aan de orde komen zijn bijvoorbeeld "Bent u geïnteresseerd om informatie over uw Parkinson bij te houden?" en "Welke klachten zijn belangrijk voor u om bij te houden?" De resultaten zullen worden gebruikt om de prioriteiten in ons onderzoek beter af te stemmen op uw behoeftes.

Hoe werkt het?
Als u akkoord gaat met deelname aan deze vragenlijst, zal u worden gevraagd een aantal vragen te beantwoorden via deze website. U bent ongeveer 30 tot 40 minuten bezig met het invullen van de hele vragenlijst. Als u wilt, kunt u de vragenlijst ook invullen met uw partner of iemand anders die u goed kent. Uw deelname is vrijwillig en u kunt op ieder moment stoppen met het invullen van de vragenlijst.

Hoe gaan wij om met uw gegevens?
We zullen uw gegevens anoniem verwerken. Dit betekent dat we de informatie die we verzamelen nooit samen opslaan met uw persoonlijke informatie (zoals naam en adres). De anonieme data zal gebruikt worden voor presentaties en (wetenschappelijke) publicaties.

Heeft u nog vragen?
Als u vragen heeft over het onderzoek, kunt u altijd contact met ons opnemen via email (info@parkinsonthuis.nl) of telefoon (085-8888 660).

Alvast hartelijk dank voor uw bijdrage!

Met vriendelijke groet,
Het ParkinsonThuis team

Uw toestemming

Voordat we van start gaan met de vragenlijst, hebben we uw toestemming nodig voor uw deelname.
Daarnaast vragen we u om te bevestigen dat u bent gediagnosticeerd met de ziekte van Parkinson door een neuroloog.

Bent u niet gediagnosticeerd met de ziekte van Parkinson, of heeft u besloten liever niet deel te nemen? In dat geval danken wij u voor uw interesse, en kunt u dit formulier sluiten.
*****

[ ] Ik heb bovenstaande informatie gelezen en ga akkoord met deelname aan dit onderzoek.

*****

[ ] Ik bevestig dat ik ben gediagnosticeerd met de ziekte van Parkinson door een neuroloog.

We beginnen met een paar algemene vragen over u. Dit is bruikbare informatie voor ons onderzoek.

1) In welk jaar bent u geboren?*

____

2) Wat is uw geslacht?*

( ) Man

( ) Vrouw

3) In welk jaar bent u door de neuroloog gediagnosticeerd met de ziekte van Parkinson?*

____

4) Gebruikt u op dit moment orale (via de mond ingenomen), voorgeschreven medicatie voor uw ziekte van Parkinson?*

( ) Ja

( ) Nee

5) Welke zorgverleners heeft u IN HET AFGELOPEN JAAR bezocht voor uw ziekte van Parkinson?*

Vink alstublieft alles aan wat van toepassing is.

[ ] Een neuroloog

[ ] Een huisarts

[ ] Een Parkinson-verpleegkundige

[ ] Een fysio- of oefentherapeut

[ ] Een diëtist

[ ] Een logopedist

[ ] Een ergotherapeut

[ ] Anders, graag omschrijven: ____

6) Ruimte voor eventuele opmerkingen: (niet verplicht)

____

____

____

____

We weten dat de ziekte van Parkinson zich niet hetzelfde uit bij iedere persoon. Daarom vragen wij u om uit een lijst te kiezen welke klachten u in de afgelopen maand heeft ervaren.

Het is belangrijk voor u om te weten dat niet iedereen met de ziekte van Parkinson deze klachten zal krijgen.

7) Welke van de volgende klachten heeft u ervaren IN DE AFGELOPEN MAAND?*

Selecteer alstublieft alle antwoorden die voor u van toepassing zijn.

[ ] Traagheid van de bewegingen

[ ] Tremor (trillen)

[ ] Stijfheid in spieren

[ ] Problemen met lopen

[ ] Moeite om te beginnen met lopen of “bevriezen” tijdens het lopen

[ ] Problemen met uw balans en/of vallen

[ ] Problemen met de fijne motoriek (zoals uw handschrift)

[ ] Problemen met uw spraak

[ ] Dyskinesieën (onvrijwillige en overmatige bewegingen, anders dan tremoren, soms beschreven als "onregelmatig schokken", "wiebelen" of "trekken")

[ ] Dystonie (oncontroleerbare en soms pijnlijke krampen en spierspasmen)

[ ] Pijn

[ ] Druppelen van speeksel

[ ] Moeite met slikken of problemen met verslikken

[ ] Verlies van uw vermogen om te proeven en/of te ruiken

[ ] Gewichtsverlies

[ ] Braken of misselijkheid

[ ] Problemen met de ontlasting

[ ] Hevige aandrang bij het plassen, zodat u zich moet haasten naar het toilet

[ ] Moeilijkheden bij de seksuele activiteit, als u het probeert

[ ] Een licht gevoel in uw hoofd, duizeligheid of slapheid wanneer u gaat staan

[ ] Overmatig zweten

[ ] Problemen met slapen

[ ] Onaangenaam gevoel in uw benen ’s avonds of tijdens rust, gepaard met de behoefte om uw benen te bewegen

[ ] Moeite met wakker blijven tijdens activiteiten, zoals werken, autorijden of eten

[ ] Vermoeidheid of een gebrek aan energie

[ ] Moeite om uw concentratie en aandacht erbij te houden

[ ] Moeilijkheden om zich recente gebeurtenissen te herinneren, of vergeten om geplande dingen te doen

[ ] Sombere stemming

[ ] Het zien of horen van dingen, waarvan u weet dat ze niet echt zijn of waarvan door anderen wordt gezegd dat ze er niet zijn

[ ] Overtuigd zijn dat er bepaalde dingen met u gebeuren, terwijl andere personen dat ontkennen

[ ] Veranderingen in gedrag, zoals overmatig gokken, eten, winkelen of meer interesse in seks

[ ] Dubbelzien

[ ] Anders (graag omschrijven): ____

[ ] Geen van bovenstaande

Sommige factoren kunnen klachten van de ziekte van Parkinson beïnvloeden (bijvoorbeeld stress, slaap, medicijnen, dieet, etc.). We weten dat dit ook verschillend kan zijn voor elk individu. Hierover gaat de volgende vraag.

8) Welke factoren hebben naar uw ervaring invloed op uw Parkinson-klachten? (negatief, positief of allebei)*

Vink graag alle vakjes aan die van toepassing zijn.

[ ] Dieet

[ ] Parkinson-medicijnen

[ ] Niet-Parkinson medicijnen, bijvoorbeeld pijnstillers

[ ] Stress

[ ] Tijd van de dag

[ ] Lichaamsbeweging

[ ] Vochtinname

[ ] Pijn

[ ] Allergieën (zoals hooikoorts, voedselallergie)

[ ] Algemeen gevoel van welbevinden

[ ] Slaap

[ ] Weer/seizoen

[ ] Stemming

[ ] Andere ziekten

[ ] Sociale relaties

[ ] Anders (graag omschrijven): ____

[ ] Geen van bovenstaande

9) Ruimte voor eventuele opmerkingen: (niet verplicht)

____

____

____

____

Sommige mensen vinden het nuttig om het verloop van hun Parkinson-klachten bij te houden, bijvoorbeeld met een dagboek of een smartphone app. Wij zijn geïnteresseerd naar uw persoonlijke mening hierover.

10) Lijkt het U weleens nuttig om het verloop van uw Parkinson-klachten bij te houden?*

( ) Ja

( ) Nee

Logic: Show/hide trigger exists.

11) Heeft u HET AFGELOPEN JAAR het verloop van uw Parkinson-klachten weleens bijgehouden?*

( ) Ja

( ) Nee

Logic: Show/hide trigger exists. Hidden unless: #11 Question "Heeft u HET AFGELOPEN JAAR het verloop van uw Parkinson-klachten weleens bijgehouden?" is one of the following answers ("Ja")

12) Op welke manier heeft u dit gedaan?*

Graag alles aanvinken wat van toepassing is.

[ ] Een papieren dagboek (bijvoorbeeld een on-off dagboek)

[ ] Een app op uw telefoon of tablet

[ ] Een meetapparaat/sensor (bijvoorbeeld een valdetector, FitBit, stappenteller, Wii-fit bord)

[ ] Een website (zoals de Parkinson-monitor van de Parkinson Vereniging)

[ ] Anders (graag omschrijven): ____

Logic: Hidden unless: #12 Question "Op welke manier heeft u dit gedaan?" is one of the following answers ("Een papieren dagboek (bijvoorbeeld een on-off dagboek)")

13) Kunt u alstublieft omschrijven wat voor soort dagboek dit was? (niet verplicht)

____

____

____

____

Logic: Hidden unless: #12 Question "Op welke manier heeft u dit gedaan?" is one of the following answers ("Een app op uw telefoon of tablet")

14) Kunt u alstublieft omschrijven wat voor soort app dit was? (niet verplicht) Als u de naam weet, kunt u deze noteren.

____

____

____

____

Logic: Hidden unless: #12 Question "Op welke manier heeft u dit gedaan?" is one of the following answers ("Een meetapparaat/sensor (bijvoorbeeld een valdetector, FitBit, stappenteller, Wii-fit bord)")

15) Kunt u alstublieft omschrijven wat voor soort meetapparaat/sensor dit was? (niet verplicht)
Als u de naam weet, kunt u deze noteren.

____

____

____

____

Logic: Hidden unless: #12 Question "Op welke manier heeft u dit gedaan?" is one of the following answers ("Een website (zoals de Parkinson-monitor van de Parkinson Vereniging)")

16) Kunt u alstublieft omschrijven wat voor soort website dit was? (niet verplicht)
Als u de naam weet, kunt u deze noteren.

____

____

____

____

Logic: Hidden unless: #11 Question "Heeft u HET AFGELOPEN JAAR het verloop van uw Parkinson-klachten weleens bijgehouden?" is one of the following answers ("Ja")

17) Wat was uw belangrijkste motivatie om het verloop van uw Parkinson-klachten bij te houden?*

____

____

____

____

Logic: Hidden unless: #11 Question "Heeft u HET AFGELOPEN JAAR het verloop van uw Parkinson-klachten weleens bijgehouden?" is one of the following answers ("Nee")

18) Wat is de belangrijkste reden dat u het verloop van uw Parkinson-klachten NIET heeft bijgehouden?*

____

____

____

____

19) Ruimte voor eventuele opmerkingen: (niet verplicht)

____

____

____

____

Page entry logic: This page will show when: (#10 Question "Lijkt het **U** weleens nuttig om het verloop van uw Parkinson-klachten bij te houden?" is one of the following answers ("Ja") AND (answer count determinants is greater than or equal to "3" OR answer count symptoms is greater than or equal to "3"))

Logic: Hidden unless: (#10 Question "Lijkt het U weleens nuttig om het verloop van uw Parkinson-klachten bij te houden?" is one of the following answers ("Ja") AND answer count symptoms is greater than or equal to "3")

Piping: Piped Values From Question 7. (Welke van de volgende klachten heeft u ervaren **IN DE AFGELOPEN MAAND**?)

20) Wij willen u vragen om een TOP 3 te maken van de Parkinson-klachten die voor u het nuttigst zouden zijn om bij te houden (bijvoorbeeld met een dagboek, app of meetapparaatje).

Belangrijk: U hoeft niet na te denken over of het op dit moment mogelijk is om het te meten, wij willen enkel van u weten wat waardevol voor u zou zijn.

Instructies: kies uit de lijst links de klacht die u het nuttigst vindt om bij te houden. Sleep deze naar de rechterbalk met uw muis.
Doe daarna hetzelfde met uw tweede en derde keus. De volgorde in de rechterbalk is voor ons WEL van belang; deze kunt u eventueel aanpassen door te slepen. *

Example:

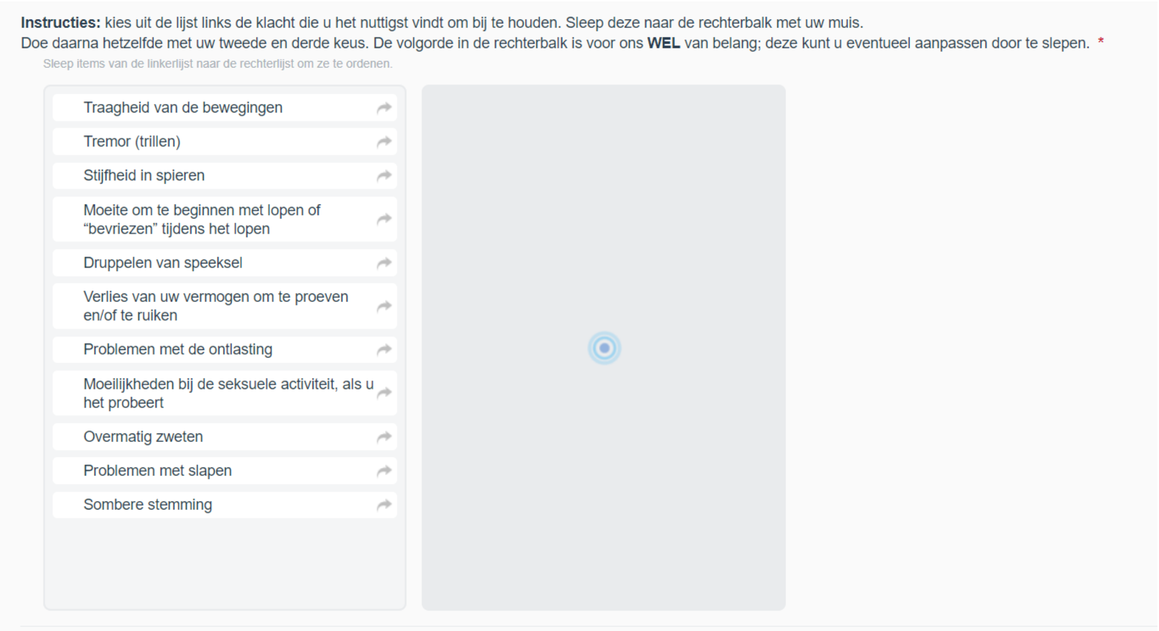

Logic: Hidden unless: (#10 Question "Lijkt het U weleens nuttig om het verloop van uw Parkinson-klachten bij te houden?" is one of the following answers ("Ja") AND answer count determinants is greater than or equal to "3")

Piping: Piped Values From Question 8. (Welke factoren hebben **naar uw ervaring** invloed op uw Parkinson-klachten? (negatief, positief of allebei))

21) Naast Parkinson-klachten, kunnen ook andere dingen belangrijk zijn om bij te houden.
Maak alstublieft een TOP 3 van andere dingen die u het nuttigst zou vinden om bij te houden in relatie tot uw Parkinson-klachten (bijvoorbeeld met een app, dagboek of meetapparaatje).

Belangrijk: U hoeft niet na te denken over of het op dit moment mogelijk is om het te meten, wij willen enkel van u weten wat waardevol voor u zou zijn.

Instructies: sleep uw eerste keuze naar de rechterbalk met uw muis. Doe daarna hetzelfde met uw tweede en derde keus.
De volgorde in de rechterbalk is voor ons WEL van belang; deze kunt u eventueel aanpassen door te slepen. *

Example:

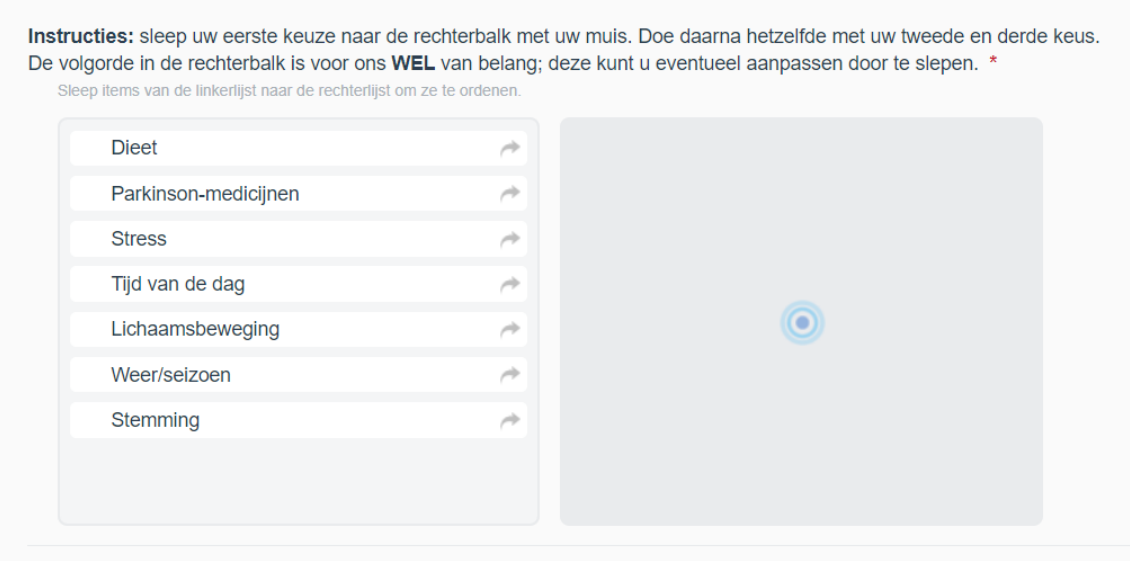

22) Ruimte voor eventuele opmerkingen: (niet verplicht)

____

____

____

____

Page entry logic: This page will show when: (#10 Question "Lijkt het **U** weleens nuttig om het verloop van uw Parkinson-klachten bij te houden?" is one of the following answers ("Ja") AND answer count symptoms is greater than or equal to "3")

Logic: Hidden unless: (#10 Question "Lijkt het U weleens nuttig om het verloop van uw Parkinson-klachten bij te houden?" is one of the following answers ("Ja") AND answer count symptoms is greater than or equal to "3")

Piping: Piped Values From Question 7. (Welke van de volgende klachten heeft u ervaren **IN DE AFGELOPEN MAAND**?)

23) Wij willen u vragen om een TOP 3 te maken van de Parkinson-klachten waar u het meeste last van heeft.

Instructies: kies uit de lijst links de klacht waarvan u het meeste last heeft. Sleep deze naar de rechterbalk met uw muis. Doe daarna hetzelfde met uw tweede en derde keus. De volgorde in de rechterbalk is voor ons WEL van belang; deze kunt u eventueel aanpassen door te slepen. *

Logic: Hidden unless: (#10 Question "Lijkt het U weleens nuttig om het verloop van uw Parkinson-klachten bij te houden?" is one of the following answers ("Ja") AND answer count symptoms is greater than or equal to "3")

Piping: Piped Values From Question 7. (Welke van de volgende klachten heeft u ervaren **IN DE AFGELOPEN MAAND**?)

24) Maak alstublieft een TOP 3 van de Parkinson-klachten die het sterkst schommelen in ernst (bijvoorbeeld door medicijninname of tijd van de dag).

Instructies: kies uit de lijst links de klacht die sterkst schommelt. Sleep deze naar de rechterbalk met uw muis. Doe daarna hetzelfde met uw tweede en derde keus. De volgorde in de rechterbalk is voor ons WEL van belang; deze kunt u eventueel aanpassen door te slepen. *

25) Ruimte voor eventuele opmerkingen: (niet verplicht)

____

____

____

____

Logic: Hidden unless: #11 Question "Heeft u HET AFGELOPEN JAAR het verloop van uw Parkinson-klachten weleens bijgehouden?" is one of the following answers ("Ja")

**Aan het begin van deze vragenlijst heeft u aangegeven dat u uw Parkinson-klachten weleens heeft bijhouden in het afgelopen jaar.
Wij willen graag nog wat meer weten over de motivaties die hierbij een rol speelden.**

Logic: Hidden unless: #11 Question "Heeft u HET AFGELOPEN JAAR het verloop van uw Parkinson-klachten weleens bijgehouden?" is one of the following answers ("Ja")

26) Ik heb het verloop van mijn Parkinson-klachten weleens bijgehouden, omdat...*

	**Sterk mee oneens**	**Mee oneens**	**Neutraal**	**Mee eens**	**Sterk mee eens**
... ik zelf in de hand wil hebben wat ik doe met mijn leven.	( )	( )	( )	( )	( )
... ik sommige aspecten van mijn leven probeer te veranderen.	( )	( )	( )	( )	( )
... ik graag baas over mijn eigen lichaam ben.	( )	( )	( )	( )	( )
... ik het interessant vind hoe bepaalde dingen in mijn leven op elkaar reageren.	( )	( )	( )	( )	( )
... het mij helpt mijn manier van leven te optimaliseren.	( )	( )	( )	( )	( )
... het mij helpt gemotiveerd te blijven om mijn doelen na te jagen.	( )	( )	( )	( )	( )

... het mij de mogelijkheid geeft om mijzelf te belonen.	( )	( )	( )	( )	( )
... het helpt voor mijn zelfdiscipline.	( )	( )	( )	( )	( )
... ik geen vertrouwen heb in het zorgsysteem/klassieke behandelingen.	( )	( )	( )	( )	( )
... ik onafhankelijk wil zijn van traditionele medische behandelingen.	( )	( )	( )	( )	( )
... ik ervan geniet om mezelf helemaal te verliezen in bezig zijn met het bijhouden van informatie.	( )	( )	( )	( )	( )
... ik het leuk vind om bezig te zijn met cijfers en statistieken.	( )	( )	( )	( )	( )
... ik het leuk vind om bezig te zijn met mijn telefoon en andere elektronische apparaten.	( )	( )	( )	( )	( )
... ik ervan geniet om de tijd te vergeten als ik ermee bezig ben.	( )	( )	( )	( )	( )
... het leuk en vermakelijk is.	( )	( )	( )	( )	( )
... ik anderen wil helpen/inspireren.	( )	( )	( )	( )	( )
... de manier waarop ik het doe interessant is voor	( )	( )	( )	( )	( )

anderen/anderen kan helpen.					
... ik mijn resultaten wil vergelijken met die van anderen.	( )	( )	( )	( )	( )
... ik mijzelf aan anderen wil laten zien.	( )	( )	( )	( )	( )

27) Tenslotte zoeken wij een paar mensen die in een groepsgesprek met andere mensen met Parkinson en een onderzoeker verder willen praten over de onderwerpen in deze vragenlijst. Mogen wij hiervoor contact met u opnemen?*

( ) Ja

( ) Nee

28) Ruimte voor eventuele opmerkingen: (niet verplicht)

____

____

____

____

Hartelijk dank voor het invullen van deze vragenlijst!
Uw bijdrage helpt ons om de prioriteiten van ons onderzoek beter af te stemmen op de behoeften van patiënten en zorgverleners.

De resultaten van deze vragenlijst ziet u rond de zomer terug op de website www.parkinsonthuis.nl en in onze nieuwsbrief.

Vriendelijke groet,
Het ParkinsonThuis team
