# Supplementary material for: Need for personalized monitoring of Parkinson’s disease: the perspectives of patients and specialized healthcare providers
Source: Front Neurol. 2023 May 4;14:1150634. doi: 10.3389/fneur.2023.1150634 (PMC10192863; doi:10.3389/fneur.2023.1150634)
Supplement: Supplementary file 1 [file data_sheet_1.zip › Data Sheet 1 - updated/Appendix A2.pdf]

Appendix A2 - survey patients English

Welcome to this online survey of the Parkinson@home trial!

What is the purpose of this survey?
Our research is focused on developing helpful tools to monitor Parkinson's disease during daily life. To do this, we need your input!
Using this survey, we want to learn what is important for people having Parkinson's disease.
We will address question such as "Would you be interested in using remote monitoring tools?" and "Which symptoms are important for you to keep track of?"
The results will be used to make sure that the priorities in our research are aligned with your needs.

How does it work?
If you give your consent to participate in this survey, you will be asked to complete several questions online.
The whole survey will take approximately 30 to 40 minutes. If this is convenient for you, you can complete the questions together with your partner or a person that knows you well.
Your participation is voluntary and you can stop filling out the survey anytime you want.

How will you handle my data?
We will process your data in an anonymous way. This means that we will never store the obtained information together with your personal information (e.g. name and address).
The anonymous data may be used for presentations and (scientific) publications.

Do you have any questions?
If you have any questions about the questionnaire or other parts of our research, you can always contact us through …

Thank you in advance for your contribution to our research!

Kind regards,
The Parkinson@home team

Your consent

Before we start, we need your consent for participation.
In addition, we need you to confirm that you are diagnosed with Parkinson's disease by a neurologist.

Are you not diagnosed with Parkinson's disease by a neurologist, or do you not want to participate? In this case, you can just close this form.
*****

[ ] I agree with participating in this survey.

*****

[ ] I confirm that I am diagnosed with Parkinson's disease by a neurologist.

We will start this survey by asking some background information about you. This is helpful for our research.

1) What year were you born?*

____

2) What is your gender?*

( ) Male

( ) Female

3) What year where you diagnosed with Parkinson's disease by a neurologist?*

____

4) Do you currently use oral, prescription medication for your Parkinson's disease?*

( ) Yes

( ) No

5) What types of health care professionals have you seen to treat your Parkinson's disease in the past year?*

Please tick all that apply.

[ ] A neurologist

[ ] A general practitioner or primary care provider

[ ] A Parkinson's Nurse Specialist

[ ] A physiotherapist

[ ] A dietitian

[ ] A speech therapist

[ ] An occupational therapist

[ ] Other (please specify): ____

6) Do you have any comments or are there any unclarities on this page of the survey?

____

____

____

____

We know that Parkinson's disease is not the same for every patient, so we start off by asking you to choose from a list which symptoms you have experienced in the last month.

It is important to know that not everyone with Parkinson's disease will experience these symptoms.

7) Have you experienced any of the following symptoms in the last month?*

Please tick all that apply.

[ ] Slowness of movement

[ ] Tremor (shaking)

[ ] Rigidity (muscle stiffness)

[ ] Walking problems

[ ] Trouble to start walking or freezing when walking

[ ] Problems with your balance and/or falling

[ ] Problems with fine motor movements (such as handwriting)

[ ] Problems with your speech

[ ] Dyskinesia (involuntary and excessive movements, other than tremor, sometimes described as "irregular jerking", "wiggling" or "twitching")

[ ] Dystonia (uncontrollable and sometimes painful muscle spasms)

[ ] Pain

[ ] Dribbling of saliva

[ ] Difficulty swallowing or problems with choking

[ ] Loss in your ability to taste or smell

[ ] Weight loss (not due to change in diet)

[ ] Vomiting or feelings of sickness (nausea)

[ ] Bowel problems

[ ] A sense of urgency to pass urine makes you rush to the toilet

[ ] Finding it difficult to have sex when you try

[ ] Feeling light headed, dizzy or weak standing from sitting or lying

[ ] Excessive sweating

[ ] Sleep problems

[ ] Restless legs (unpleasant sensations in your legs at night or while resting, and a feeling that you need to move)

[ ] Finding it difficult to stay awake during activities such as working, driving or eating

[ ] Fatigue or lack of energy

[ ] Difficulty concentrating or staying focussed

[ ] Problems remembering things that have happened recently or forgetting to do things

[ ] Depressed mood

[ ] Seeing or hearing things that you know or are told are not there

[ ] Believing things are happening to you that other people say are not true

[ ] Impulsive or compulsive behaviour (such as excessive gambling, eating, shopping or more interest in sex)

[ ] Double vision

[ ] Anders (graag omschrijven): ____

[ ] None of the above

Several factors may influence the state of your Parkinson's disease (for example stress, sleep, medication use, diet, etc.). We know that this can also be different for every individual. That is what the last 2 questions of this survey are about.

8) From your experience, which factors affect the state of your Parkinson's (negatively, positively or both)?*

Please tick all that apply.

[ ] Diet

[ ] Parkinson-medicatie

[ ] Non-Parkinson's medication e.g. for pain relief

[ ] Stress

[ ] Time of day

[ ] Physical exercise

[ ] Hydration

[ ] Pain

[ ] Allergies (e.g. hay fever, food)

[ ] General sense of well-being

[ ] Sleep

[ ] Weather/season

[ ] Mood

[ ] Other illness

[ ] Relationships

[ ] Anders (graag omschrijven): ____

[ ] None of the above

9) Do you have any comments or are there any unclarities on this page of the survey?

____

____

____

____

Some people find it useful to record information about their Parkinson's disease during their daily life. They for example keep a diary or use a smartphone application to monitor symptoms, problems or other factors related their Parkinson's disease. We are interested in your personal opinion about this.

10) Are you interested in monitoring your Parkinson's disease during your daily life?*

( ) Yes

( ) No

Logic: Show/hide trigger exists.

11) DURING THE PAST YEAR, have your recorded information about your Parkinson's disease during your daily life?*

( ) Yes

( ) No

Logic: Show/hide trigger exists. Hidden unless: #11 Question "During the past year, have your recorded information about your Parkinson's disease during your daily life?" is one of the following answers ("Yes")

12) Which of the following methods or tools did you use to record information about your Parkinson's?*

Please tick all that apply.

[ ] A paper diary (for example the on-off diary)

[ ] A smartphone/tablet application

[ ] A monitoring device/sensors (for example a fall detector, FitBit, step counter, smartwatch, Wii-fit board)

[ ] A website (like the Parkinson-monitor)

[ ] Other (please specify): ____

Logic: Hidden unless: #12 Question "Which of the following methods or tools did you use to record information about your Parkinson's?" is one of the following answers ("A paper diary (for example the on-off diary)")

13) Can you please explain what kind of paper diary you use?

____

____

____

____

Logic: Hidden unless: #12 Question "Which of the following methods or tools did you use to record information about your Parkinson's?" is one of the following answers ("A smartphone/tablet application")

14) Can you please explain what kind of application you use(d)? If you know the name of the app, please note.

____

____

____

____

Logic: Hidden unless: #12 Question "Which of the following methods or tools did you use to record information about your Parkinson's?" is one of the following answers ("A monitoring device/sensors (for example a fall detector, FitBit, step counter, smartwatch, Wii-fit board)")

15) Can you please explain what sensor or wearable you used? If you know the name, please note.

____

____

____

____

Logic: Hidden unless: #12 Question "Which of the following methods or tools did you use to record information about your Parkinson's?" is one of the following answers ("A website (like the Parkinson-monitor)")

16) What kind of website did you use? Can you please elaborate?

____

____

____

____

Logic: Hidden unless: #11 Question "During the past year, have your recorded information about your Parkinson's disease during your daily life?" is one of the following answers ("Yes")

17) What is your main motivation for monitoring your Parkinson’s disease?*

____

____

____

____

Logic: Hidden unless: #11 Question "During the past year, have your recorded information about your Parkinson's disease during your daily life?" is one of the following answers ("No")

18) What is the main reason that you have NOT monitored your Parkinson's disease?*

____

____

____

____

19) Do you have any comments or are there any unclarities on this page of the survey?

____

____

____

____

Page entry logic: This page will show when: (#10 Question "Are you interested in monitoring your Parkinson's disease during your daily life?" is one of the following answers ("Yes") AND (answer count determinants is greater than or equal to "3" OR answer count symptoms is greater than or equal to "3"))

Logic: Hidden unless: (#10 Question "Are you interested in monitoring your Parkinson's disease during your daily life?" is one of the following answers ("Yes") AND answer count symptoms is greater than or equal to "3")

Piping: Piped Values From Question 7. (Have you experienced any of the following symptoms in the last month?)

20) We would like to ask you to create a TOP 3 of Parkinson symptoms that you would find most useful to track (for example using a diary, app or sensor).

Important: we don't want you worry about whether it would currently be possible to measure these symptoms, we only would like to know what would be valuable for you.*

________Slowness of movement

________Tremor (shaking)

________Rigidity (muscle stiffness)

________Trouble to start walking or freezing when walking

________Problems with your balance and/or falling

________Problems with fine motor movements (such as handwriting)

________Problems with your speech

________Dyskinesia (involuntary and excessive movements, other than tremor, sometimes described as "irregular jerking", "wiggling" or "twitching")

________Dystonia (uncontrollable and sometimes painful cramps and spasms)

________Pain

________Dribbling of saliva

________Difficulty swallowing or problems with choking

________Loss in your ability to taste or smell

________Weight loss (not due to change in diet)

________Vomiting or feelings of sickness (nausea)

________Bowel problems

________A sense of urgency to pass urine makes you rush to the toilet

________Finding it difficult to have sex when you try

________Feeling light headed, dizzy or weak standing from sitting or lying

________Excessive sweating

________Sleep problems

_________Restless legs (unpleasant sensations in your legs at night or while resting, and a feeling that you need to move)

_________Finding it difficult to stay awake during activities such as working, driving or eating

_________Fatigue or lack of energy

_________Difficulty concentrating or staying focussed

_________Problems remembering things that have happened recently or forgetting to do things

_________Depressed mood

_________Seeing or hearing things that you know or are told are not there

_________Believing things are happening to you that other people say are not true

_________Impulsive or compulsive behaviour (such as excessive gambling, eating, shopping or more interest in sex)

_________Double vision

**Logic: Hidden unless: (#10 Question "Are you interested in monitoring your Parkinson's disease during your daily life?" is one of the following answers ("Yes") AND answer count determinants is greater than or equal to "3")**
Piping: Piped Values From Question 8. (From your experience, which factors affect the state of your Parkinson's (negatively, positively or both)?)

21) Besides Parkinson symptoms, other aspects can also be important to track. We would like to ask you to create a TOP 3 of other things that you would find useful to track in relation to your Parkinson symptoms(for example using a diary, app or sensor).

Important: we don’t want you worry about whether it would currently be possible to measure these symptoms, we only would like to know what would be valuable for you.*

_________Slowness of movement

_________Tremor (shaking)

_________Rigidity (muscle stiffness)

_________Trouble to start walking or freezing when walking

_________Problems with your balance and/or falling

_________Problems with fine motor movements (such as handwriting)

_________Problems with your speech

_________Dyskinesia (involuntary and excessive movements, other than tremor, sometimes described as “irregular jerking”, “wiggling” or “twitching”)

_________Dystonia (uncontrollable and sometimes painful cramps and spasms)

_________Pain

_________Dribbling of saliva

________Difficulty swallowing or problems with choking

________Loss in your ability to taste or smell

________Weight loss (not due to change in diet)

________Vomiting or feelings of sickness (nausea)

________Bowel problems

________A sense of urgency to pass urine makes you rush to the toilet

________Finding it difficult to have sex when you try

________Feeling light headed, dizzy or weak standing from sitting or lying

________Excessive sweating

________Sleep problems

________Restless legs (unpleasant sensations in your legs at night or while resting, and a feeling that you need to move)

________Finding it difficult to stay awake during activities such as working, driving or eating

________Fatigue or lack of energy

________Difficulty concentrating or staying focussed

________Problems remembering things that have happened recently or forgetting to do things

________Depressed mood

________Seeing or hearing things that you know or are told are not there

________Believing things are happening to you that other people say are not true

________Impulsive or compulsive behaviour (such as excessive gambling, eating, shopping or more interest in sex)

________Double vision

22) Do you have any comments or are there any unclarities on this page of the survey?

____

____

____

____

Page entry logic: This page will show when: (#10 Question "Are you interested in monitoring your Parkinson's disease during your daily life?" is one of the following answers ("Yes") AND answer count symptoms is greater than or equal to "3")

Logic: Hidden unless: (#10 Question "Are you interested in monitoring your Parkinson's disease during your daily life?" is one of the following answers ("Yes") AND answer count symptoms is greater than or equal to "3")

Piping: Piped Values From Question 7. (Have you experienced any of the following symptoms in the last month?)

23) We would like to ask you to create a TOP 3 of Parkinson symptoms that are most burdensome for you.*

________Slowness of movement

________Tremor (shaking)

________Rigidity (muscle stiffness)

________Trouble to start walking or freezing when walking

________Problems with your balance and/or falling

________Problems with fine motor movements (such as handwriting)

________Problems with your speech

________Dyskinesia (involuntary and excessive movements, other than tremor, sometimes described as “irregular jerking”, “wiggling” or “twitching”)

________Dystonia (uncontrollable and sometimes painful cramps and spasms)

________Pain

________Dribbling of saliva

________Difficulty swallowing or problems with choking

________Loss in your ability to taste or smell

________Weight loss (not due to change in diet)

________Vomiting or feelings of sickness (nausea)

________Bowel problems

________A sense of urgency to pass urine makes you rush to the toilet

________Finding it difficult to have sex when you try

________Feeling light headed, dizzy or weak standing from sitting or lying

________Excessive sweating

________Sleep problems

________Restless legs (unpleasant sensations in your legs at night or while resting, and a feeling that you need to move)

________Finding it difficult to stay awake during activities such as working, driving or eating

_________Fatigue or lack of energy

_________Difficulty concentrating or staying focussed

_________Problems remembering things that have happened recently or forgetting to do things

_________Depressed mood

_________Seeing or hearing things that you know or are told are not there

_________Believing things are happening to you that other people say are not true

_________Impulsive or compulsive behaviour (such as excessive gambling, eating, shopping or more interest in sex)

_________Double vision

Logic: Hidden unless: (#10 Question "Are you interested in monitoring your Parkinson's disease during your daily life?" is one of the following answers ("Yes") AND answer count symptoms is greater than or equal to "3")

Piping: Piped Values From Question 7. (Have you experienced any of the following symptoms in the last month?)

24) We would like to ask you to create a TOP 3 of Parkinson symptoms that most strongly fluctuate in severity (for example in relation to of medication intake or time of the day).*

_________Slowness of movement

_________Tremor (shaking)

_________Rigidity (muscle stiffness)

_________Trouble to start walking or freezing when walking

_________Problems with your balance and/or falling

_________Problems with fine motor movements (such as handwriting)

_________Problems with your speech

_________Dyskinesia (involuntary and excessive movements, other than tremor, sometimes described as “irregular jerking”, “wiggling” or “twitching”)

_________Dystonia (uncontrollable and sometimes painful cramps and spasms)

_________Pain

_________Dribbling of saliva

_________Difficulty swallowing or problems with choking

_________Loss in your ability to taste or smell

_________Weight loss (not due to change in diet)

_________Vomiting or feelings of sickness (nausea)

_________Bowel problems

________A sense of urgency to pass urine makes you rush to the toilet

________Finding it difficult to have sex when you try

________Feeling light headed, dizzy or weak standing from sitting or lying

________Excessive sweating

________Sleep problems

________Restless legs (unpleasant sensations in your legs at night or while resting, and a feeling that you need to move)

________Finding it difficult to stay awake during activities such as working, driving or eating

________Fatigue or lack of energy

________Difficulty concentrating or staying focussed

________Problems remembering things that have happened recently or forgetting to do things

________Depressed mood

________Seeing or hearing things that you know or are told are not there

________Believing things are happening to you that other people say are not true

________Impulsive or compulsive behaviour (such as excessive gambling, eating, shopping or more interest in sex)

________Double vision

25) Do you have any comments or are there any unclarities on this page of the survey?

____

____

____

____

Logic: Hidden unless: #11 Question "During the past year, have your recorded information about your Parkinson's disease during your daily life?" is one of the following answers ("Yes")

In the beginning of this questionnaire, you filled in you have tracked your Parkinson in the last year. We now want to ask what your motivation was to do so.

Logic: Hidden unless: #11 Question "During the past year, have your recorded information about your Parkinson's disease during your daily life?" is one of the following answers ("Yes")

26) I have collected information about my Parkinon symptoms, because...*

	**Disagree strongly**	**Disagree a little**	**Neither agree nor disagree**	**Agree a little**	**Agree strongly**
... I want to control what I'm doing with my life.	( )	( )	( )	( )	( )
... I try to manipulate certain aspects in my life.	( )	( )	( )	( )	( )
... I enjoy being my own master.	( )	( )	( )	( )	( )
... I'm interested in how certain things in (my) life interact.	( )	( )	( )	( )	( )
... it helps me to optimize the way I'm living.	( )	( )	( )	( )	( )
... it motivates me to keep on working for a goal.	( )	( )	( )	( )	( )
... it allows me to reward myself.	( )	( )	( )	( )	( )

... it facilitates my self-discipline.	( )	( )	( )	( )	( )
... I don't trust in the healthcare system/classic therapies.	( )	( )	( )	( )	( )
... I want to be independent from traditional medical treatments.	( )	( )	( )	( )	( )
... I enjoy getting lost totally in self-tracking activities.	( )	( )	( )	( )	( )
... I like playing around with numbers/statistics etc.	( )	( )	( )	( )	( )
... I like playing around with my smartphone/technical device etc.	( )	( )	( )	( )	( )
... I enjoy forgetting about time while I do so.	( )	( )	( )	( )	( )
... it is fun and entertaining.	( )	( )	( )	( )	( )
... I want to help/inspire others.	( )	( )	( )	( )	( )
... the way I'm doing it is interesting for others/might help others.	( )	( )	( )	( )	( )
... I want to compare my results to others.	( )	( )	( )	( )	( )
... I want to present myself to others.	( )	( )	( )	( )	( )

27) Would you be willing to participate in a focus group to further discuss the topics of this survey?*

( ) Yes

( ) No

28) Do you have any comments or are there any unclarities on this page of the survey?

____

____

____

____

Thank you very much for completing this survey!
Your response will help us to prioritize our research in line with needs from patients and health care professionals.

Are you interested in our research? Visit … for more information!

Best regards,
The Parkinson@Home team
