# Supplementary material for: Need for personalized monitoring of Parkinson’s disease: the perspectives of patients and specialized healthcare providers
Source: Front Neurol. 2023 May 4;14:1150634. doi: 10.3389/fneur.2023.1150634 (PMC10192863; doi:10.3389/fneur.2023.1150634)
Supplement: Supplementary file 1 [file data_sheet_1.zip › Data Sheet 1 - updated/Appendix A3.pdf]

# Appendix A3 - survey healthcare providers (Dutch)

---

**Welkom bij deze online vragenlijst van de ParkinsonThuis studie!**

**Wat is het doel van deze vragenlijst?**
**Ons onderzoek richt zich op het ontwikkelen van nieuwe manieren om de ziekte van Parkinson te monitoren in het dagelijks leven. Met deze vragenlijst brengen wij in beeld aan welke informatie u, als fysio- of oefentherapeut, behoefte heeft om mensen met de ziekte van Parkinson optimaal te kunnen behandelen.**

**Hoeveel tijd kost deelname?**
**U bent ongeveer 10 minuten bezig om de hele vragenlijst in te vullen.**

**Hoe gaan wij om met uw gegevens?**
**Alle gegevens zullen anoniem worden verwerkt en zullen nooit bewaard worden samen met uw persoonlijke informatie, zoals uw naam en adres. De gegevens zullen worden gebruikt voor wetenschappelijke publicaties.**

**Heeft u nog vragen?**
**Als u vragen heeft over dit onderzoek, kunt u altijd contact met ons opnemen per e-mail (info@parkinsonthuis.nl) of telefoon (085 8888 660).**

**Bij voorbaat dank voor uw waardevolle bijdrage!**

**Hartelijke groet,**
**Het ParkinsonThuis team**

**Uw toestemming**
**Voordat u begint met de vragenlijst, hebben we uw toestemming nodig voor deelname. Daarnaast vragen we u om te bevestigen dat u momenteel werkzaam bent als fysio- of oefentherapeut.**

**Heeft u besloten liever niet deel te nemen? In dat geval danken wij u voor uw interesse, en kunt u dit formulier sluiten.***

[ ] Ik heb bovenstaande informatie gelezen en ga akkoord met deelname aan dit onderzoek.

**En:**

[ ] Ik bevestig dat ik momenteel werkzaam ben als fysio- of oefentherapeut.

---

**1) Hoeveel unieke patiënten met de ziekte van Parkinson behandelt u gemiddeld per jaar?**

( ) 0-5

( ) 5-10

( ) 10-15

( ) 15+

## Zorgverleners vragen hun Parkinson-patiënten weleens om in het dagelijks leven informatie te verzamelen over het beloop van hun klachten, bijvoorbeeld met een "dagboek" of smartphone app. We zijn benieuwd naar uw ervaring hiermee.

**Logic: Show/hide trigger exists.**

**2) Raadt u uw Parkinson-patiënten weleens aan om het beloop van hun klachten bij te houden in het dagelijks leven?***

( ) Ja

( ) Nee

**Logic: Hidden unless: #2 Question "Raadt u uw Parkinson-patiënten weleens aan om het beloop van hun klachten bij te houden in het dagelijks leven?" is one of the following answers ("Ja")**

**3) Welke van de volgende middelen raadt u uw patiënten weleens aan?***

Selecteer alstublieft alles dat van toepassing is, en omschrijf de gekozen antwoorden.

[ ] Een papieren dagboek: ____________________________________________

[ ] Een smartphone/tablet applicatie: ____________________________________________

[ ] Draagbare sensor(en): ____________________________________________

[ ] Een website (zoals de Parkinson-monitor): ____________________________________________

[ ] Anders: ____________________________________________

## 4) Ruimte voor eventuele opmerkingen: (niet verplicht)

________________________________________

________________________________________

________________________________________

________________________________________

---

**Wij willen graag weten waaraan u, als therapeut, behoefte heeft wat betreft het monitoren van de ziekte van Parkinson. In de volgende vraag gaan we in op Parkinson-klachten, in de vraag erna komen factoren aan bod die klachten kunnen beïnvloeden (zoals dieet en lichaamsbeweging).**

**5) Om optimale zorg aan Parkinson-patiënten te bieden, wat zouden VOOR U de 3 MEEST WAARDEVOLLE Parkinson-klachten zijn om te monitoren in het dagelijks leven van patiënten? (bijvoorbeeld met een dagboek, app of sensor)**

**Belangrijk: U hoeft niet na te denken of het mogelijk is om het te meten, wij willen enkel weten wat waardevol voor u zou zijn.**

**Instructies: sleep met uw muis de drie meest waardevolle klachten van de balk links naar de rechterbalk. De volgorde in de rechterbalk is voor ons wel van belang; deze kunt u eventueel nog aanpassen door te slepen.***

________Bradykinesie / bewegingstraagheid

________Tremor

________Rigiditeit / spierstijfheid

________Freezing

________Problemen met lopen

________Problemen met balans en/of vallen

________Problemen met fijne motoriek

________Problemen met spraak

________Dyskinesie

________Dystonie

________Pijn

________Kwijlen van speeksel

________Slikproblemen

________Verlies van vermogen om te proeven en/of ruiken

________Gewichtsverlies

________Overgeven of misselijkheid

________Problemen met de ontlasting

________Plasproblemen (zoals urgency klachten)

________Seksuele problemen

________Orthostatische duizeligheid

________Overmatig zweten

________Slaapproblemen

________Restless legs

________Moeite met wakker blijven tijdens activiteiten

________Vermoeidheid

________Problemen met concentratie

________Problemen met geheugen

________Depressieve stemming

________Hallucinaties

________Wanen

________Impulsief of compulsief gedrag (zoals overmatig gokken)

________Dubbelzien

________Anders (graag omschrijven in de volgende vraag)

________Geen van bovenstaande

**6) Kunt u alstublieft een korte motivatie geven voor uw eerste keuze? (Niet verplicht)**

________________________________________________

________________________________________________

________________________________________________

________________________________________________

**7) Kunt u alstublieft een korte motivatie geven voor uw tweede keuze? (Niet verplicht)**

________________________________________________

________________________________________________

________________________________________________

________________________________________________

**8) Kunt u alstublieft een korte motivatie geven voor uw derde keuze? (Niet verplicht)**

________________________________________________

________________________________________________

________________________________________________

________________________________________________

**9) Ruimte voor eventuele opmerkingen: (niet verplicht)**

________________________________________________

________________________________________________

________________________________________________

________________________________________________

---

## Ook factoren die het beloop van Parkinson-klachten beïnvloeden, kunnen nuttig zijn om bij te houden.

**10) Om optimale zorg aan Parkinson-patiënten te bieden, wat zijn VOOR U de 3 MEEST WAARDEVOLLE factoren om te monitoren in het dagelijks leven van patiënten? (bijvoorbeeld met een dagboek, app of sensor)**

**Belangrijk: U hoeft niet na te denken of het mogelijk is om het te meten, wij willen enkel weten wat waardevol voor u zou zijn.**

**Instructies: sleep met uw muis de drie meest waardevolle factoren van de balk links naar de rechterbalk. De volgorde in de rechterbalk is voor ons wel van belang; deze kunt u eventueel nog aanpassen door te slepen.***

________Dieet

________Parkinson-medicatie

________Verandering van medicatie

________Missen van een dosis medicatie

________Niet-Parkinson medicatie

________Stress

________Tijd van de dag

________Lichaamsbeweging

________Vochtinname

________Pijn

________Allergieën (zoals hooikoorts, eten)

________Algemeen gevoel van welbevinden

________Slaap

________Weer/seizoen

________Stemming

________Andere ziekten

________Sociale relaties

________Anders (graag omschrijven in volgende vraag)

________Geen van bovenstaande

**11) Kunt u alstublieft een korte motivatie geven voor uw eerste keuze? (Niet verplicht)**

___________________________________________

___________________________________________

___________________________________________

___________________________________________

**12) Kunt u alstublieft een korte motivatie geven voor uw tweede keuze? (Niet verplicht)**

___________________________________________

___________________________________________

___________________________________________

___________________________________________

**13) Kunt u alstublieft een korte motivatie geven voor uw derde keuze? (Niet verplicht)**

___________________________________________

___________________________________________

___________________________________________

___________________________________________

**14) Ruimte voor eventuele opmerkingen: (niet verplicht)**

___________________________________________

___________________________________________

___________________________________________

___________________________________________

**Steeds meer onderzoek richt zich op de inzet van draagbare sensoren om de ziekte van Parkinson te monitoren in het dagelijks leven. U kunt hierbij denken aan horloges die tremoren meten, een ketting die vallen meet, etc. Wij horen graag hoe u als zorgverlener hierover denkt.**

**15) Ik ben van mening dat draagbare sensoren de potentie hebben om mij te helpen bij het monitoren van mijn Parkinson-patiënten.***

( ) 1: Sterk mee oneens ( ) 2 ( ) 3 ( ) 4 ( ) 5 ( ) 6 ( ) 7: Sterk mee eens

**16) Wat is naar uw mening het belangrijkste voordeel van het gebruik van draagbare sensoren om Parkinson-patiënten te monitoren? ***

_____________________________________________

_____________________________________________

_____________________________________________

_____________________________________________

**17) Wat is naar uw mening het belangrijkste obstakel bij het gebruik van draagbare sensoren om Parkinson-patiënten te monitoren? ***

_____________________________________________

_____________________________________________

_____________________________________________

_____________________________________________

**18) Tenslotte zoeken wij een paar mensen die in een groepsgesprek met andere therapeuten en een onderzoeker verder willen praten over de onderwerpen in deze vragenlijst. Mogen wij u hiervoor uitnodigen? ***

( ) Ja

( ) Nee

**19) Ruimte voor eventuele opmerkingen: (niet verplicht)**

________________________________________

________________________________________

________________________________________

________________________________________

---

**Hartelijk dank voor het invullen van deze vragenlijst!**
**Uw input helpt ons om de prioriteiten van ons onderzoek beter af te stemmen op de behoeften van patiënten en zorgverleners.**

**Wilt u meer weten? Bezoek dan onze website www.parkinsonthuis.nl!**

**Vriendelijke groet,**
**Het ParkinsonThuis team**

---
