# Supplementary material for: Need for personalized monitoring of Parkinson’s disease: the perspectives of patients and specialized healthcare providers
Source: Front Neurol. 2023 May 4;14:1150634. doi: 10.3389/fneur.2023.1150634 (PMC10192863; doi:10.3389/fneur.2023.1150634)
Supplement: Supplementary file 1 [file data_sheet_1.zip › Data Sheet 1 - updated/Appendix A4.pdf]

Appendix A4 - survey healthcare providers (English)

Welcome to this online survey of the Parkinson@home trial!

What is the purpose of this survey?
Our research is focused on developing helpful tools to monitor Parkinson's disease during daily life. To do this, we need your input!
We are interested to hear from you which aspects of PD should be monitored to assist you as a physiotherapist in providing optimal care to patients.

How long does it take?
The whole survey will take approximately 10 minutes.

How will the data be used?
All data will be processed anonymously; we will never store any information collected through this survey together with your personal information (e.g. name and address). The anonymous data will be used for publications.

Do you have any questions?
If you have any questions about the survey or other parts of our research, you can always contact us through …

Thank you in advance for your contribution to our research!

Best regards,
The Parkinson@home team

Your consent
Before we start, we need your consent for taking part in this survey.
In addition, we need you to confirm that you are currently working as a physiotherapist.

Do you not want to participate? In this case, we thank you for your interest, and you can just close this form.*

[ ] I have read the information stated above and agree with participating in this survey.

And:

[ ] I confirm that I am currently working as a physiotherapist.

---

1) How many Parkinson's patients do you treat annually?

( ) 0-5

( ) 5-10

( ) 10-15

( ) 15+

Healthcare providers sometimes ask their Parkinson patients to collect information on the course of their symptoms in daily life, for example using a diary or smartphone application. We are interested in your experience with this.

Logic: Show/hide trigger exists.

2) Do you sometimes recommend your patients to record information about their Parkinson during their daily lives? *

( ) Yes

( ) No

Logic: Hidden unless: #2 Question "Do you currently recommend your patients to record information about their Parkinson during their daily lives? " is one of the following answers ("Yes")

3) Which of the following tools do you recommend to patients? *

Please select all that apply and specify the items you selected.

[ ] A paper diary: ____

[ ] A smartphone/tablet application:
____

[ ] A wearable sensor device: ____

[ ] A website (like the Parkinson-monitor):
____

[ ] Other: ____

4) Do you have any comments?

____

____

____

____

We would like to learn about your needs as a therapist regarding monitoring Parkinson's disease. We will first ask about Parkinson symptoms, and in the subsequent question we will address aspects that can influence symptoms (such as diet and physical activity).

5) In order to provide optimal care to Parkinson patients, what are IN YOUR OPINION the 3 MOST IMPORTANT Parkinson symptoms to monitor in the patients' daily lives? (for example using a diary, app or sensor)

Important: we don't want you worry about whether it would currently be possible to measure these symptoms, we only would like to know what would be valuable for you.*

________Slowness of movement

________Tremor

________Rigidity (muscle stiffness)

________Trouble to start walking or freezing when walking

________Problems with walking

________Problems with balance and/or falling

________Problems with fine motor movements

________Problems with speech

________Dyskinesia

________Dystonia

________Pain

________Dribbling of saliva

________Difficulty swallowing or problems with choking

________Loss in your ability to taste or smell

________Weight loss (not due to change in diet)

________Vomiting or nausea

________Bowel problems

________A sense of urgency to pass urine makes you rush to the toilet

________Finding it difficult to have sex when you try

________Feeling light headed, dizzy or weak standing from sitting or lying

________Excessive sweating

________Sleep problems

________Restless legs

________Finding it difficult to stay awake during activities such as working, driving or eating

________Fatigue or lack of energy

________Difficulty concentrating or staying focussed

________Problems remembering things

________Depressed mood

________Hallucinations

________Delusions

________Impulsive or compulsive behaviour

________Double vision

________Other (please specify)

________None of the above

6) Could you please give a brief motivation for your first choice?

____

____

____

____

7) Could you please give a brief motivation for your second choice?

____

____

____

____

8) Could you please give a brief motivation for your third choice?

____

____

____

____

9) Do you have any comments?

____

____

____

____

In addition, aspects that influence the course of Parkinson symptoms can also be useful to monitor.

10) In order to provide optimal care to Parkinson patients, what are IN YOUR OPINION the 3 MOST IMPORTANT aspects to monitor in the patients' daily lives? (for example using a diary, app or sensor)*

Important: we don't want you worry about whether it would currently be possible to measure these symptoms, we only would like to know what would be valuable for you.*

________Diet

________Parkinson medication

________Change of medication

________Missing doses of medication

________Non-Parkinson's medication

________Stress

________Time of the day

________Physical exercise

________Hydration

________Pain

________Allergies (e.g. hay fever, food)

________General sense of well-being

________Sleep

________Weather/season

________Mood

________Other illness

________Social relationships

________Other (please specify)

________None of the above

11) Could you please give a brief motivation for your first choice?

____

____

____

____

12) Could you please give a brief motivation for your second choice?

____

____

____

____

13) Could you please give a brief motivation for your third choice?

____

____

____

____

14) Do you have any comments or are there any unclarities on this page of the survey?

____

____

____

____

More and more research is focused on wearable sensors to monitor Parkinson's disease during daily life. You can think of, for example, a watch that measures tremor, a necklace that measures fall incidents, etc. We would like to know your opinion as healthcare provider.

15) I believe that wearable sensors have the potential to help me monitor my Parkinson patients.*

( ) 1: Strongly disagree ( ) 2 ( ) 3 ( ) 4 ( ) 5 ( ) 6 ( ) 7: Strongly agree

16) What is in your opinion the main benefit of using wearable sensors to monitor Parkinson patients? *

____

____

____

____

17) What is in your opinion the main barrier of using wearable sensors to monitor Parkinson patients? *

____

____

____

____

18) Would you be willing to participate in a focus group to further discuss the topics of this survey? *

( ) Yes

( ) No

19) Do you have any comments?

____

____

____

____

Thank you very much for completing this survey!
Your response will help us to prioritize our research in line with needs from patients and health care professionals.

Are you interested in our research? Visit … for more information!

Best regards,
The Parkinson@home team
