# Supplementary material for: Need for personalized monitoring of Parkinson’s disease: the perspectives of patients and specialized healthcare providers
Source: Front Neurol. 2023 May 4;14:1150634. doi: 10.3389/fneur.2023.1150634 (PMC10192863; doi:10.3389/fneur.2023.1150634)
Supplement: Supplementary file 1 [file data_sheet_1.zip › Data Sheet 1 - updated/Appendix B1.pdf]

Appendix B1 - Focus group guide – patients (English)

Introduction (15 min)

Welcome and practical information

1. Welcome and introduction own background.
2. Short explanation about goal meeting and planning (room for break).
3. Explain that the whole meeting will be video-recorded → let everybody sign informed consent.
4. Room for questions.

Introduction participants

1. Start video recording
2. Ask everyone to introduce him-/herself: name, residence, earlier experience with research?

Introduction of focus group topic

1. As wearable sensors become smaller and cheaper, more and more people are starting to use them. A well-known example is FitBit. Because wearable sensors can measure all movements, they could also be valuable for people with Parkinson's disease.
2. In the survey, we found that a lot of people are already tracking their PD, and an important motivation was to discuss the information with their neurologist, nurse or physical therapist. The "good old" paper diary is still the most frequently used tool.
3. In this focus group, we are going to address the question: "How could wearable sensors support you in your communication with care providers? (e.g. neurologist, nurse, physical therapist)"
4. To do this, we will complete the diagram together step-by-step: first we will explore which information you find important to share with your care providers (GOALS), and which tools you have already used for this. Then, we will talk about things that are difficult for you (CHALLENGES), and things that already go well in communicating about your condition with care providers (THINGS THAT GO WELL). Next, we will talk about the advantages that wearable sensors could offer (ADVANTAGES OF WS), and which disadvantages you see (PROBLEMS WITH WS). We will finalize the discussion by discussing what the ideal tool should look like.

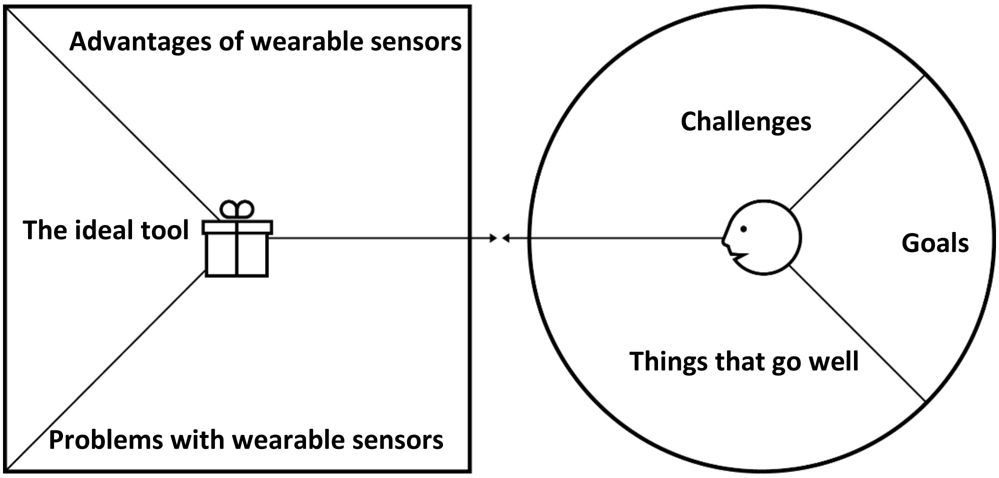

Discussion (40 min)

Instructions

1. Make participants feel comfortable: invite everyone to share their opinions, and emphasize that there are no right or wrong answers, but that we are interested to hear everyone's thoughts and experiences. Feel free to share both positive and negative feedback. We don't have to come to an agreement about everything, but show respect for each other's opinions.
2. Role caregivers: explain that, in this focus group, we are mainly interested in the perspective of patients.
3. Explain own roles: chairman (because of limited time, I might sometimes steer the conversation a little bit to make sure all topics are covered) and note taker (for later analysis).

Part 1: explore current situation (15 min)

1. Further explore goal: Has your neurologist/physical therapist/nurse ever asked you to keep track of your PD in daily life? What do you think is important to communicate with your care providers about your condition? Why is this important? (place on GOALS)
2. How do you usually do this? Have you used any tools to track your PD in daily life? (e.g. paper diary, smartphone application, website)
3. Explore challenges related to this: Did you experience any difficulties with using these tools/communicating about your condition with care providers? (examples include because it takes too much time/energy, it is not clear what care providers want to know, it is difficult to write) (place on CHALLENGES)
4. Explore facilitators: do you have an example of when the information you collected was helpful for your treatment? How did it help? (place on THINGS THAT GO WELL)
5. Ask if the group thinks we covered the most important points of this part of the diagram.

Part 2: how can wearable sensors help communicating with care providers? (25 min)

1. Give a short explanation about wearable sensors: wearable sensors are small devices that can be worn on different body locations, for example as a watch, around the waist or as a strap around the ankle. They can continuously measure your movements, and send the information via internet. This could be used to share information with your care providers. For this discussion, it is important that we want to know what you are INTERESTED in; you don't have to think about whether it is POSSIBLE with wearable sensors.
2. Does anyone have any experience with using wearable sensors? (other than already mentioned in part 1, does not have to be Parkinson-related)
3. Which advantages do you think that wearable sensors can offer in your communication with care providers? (this can be pain relievers (related to mentioned challenges) or gain creators (benefits unrelated to mentioned problems/new opportunities)). (place on ADVANTAGES)
4. Which problems/challenges do you see related to the use of wearable sensors? (place on PROBLEMS)

BREAK

5. Ask if the group thinks we covered the most important ADVANTAGES and PROBLEMS.
6. Rank ADVANTAGES and PROBLEMS: which advantages and problems do you consider most important?
7. For 2 most important ADVANTAGES → what should the tool look like to have these advantages? (place on THE IDEAL TOOL) Minimal topics to cover:
    a. Active versus passive monitoring?
    b. How should information be shared with care providers?
    c. When would you trust measurements?
8. For 2 most important BARRIERS → what should the system look like to overcome these barriers? (place on THE IDEAL TOOL)

Part 3: other questions

1. How do you think that this tool would influence your relationship with your care providers? Do you think it would have an effect on how often you see your care providers?
2. What would be the most important requirement before you would start to use such a tool?

Closure (5 min)

1. Give a brief summary of important results as is shown by the diagram. Ask if this is a good reflection of the discussion, and give brief room for participants to comment.
2. Thank everyone for contribution. Hand out presents. Let participants complete form for reimbursement travel expenses.
3. For those who are interested: short talk about research on wearable sensors.
