# Supplementary material for: Need for personalized monitoring of Parkinson’s disease: the perspectives of patients and specialized healthcare providers
Source: Front Neurol. 2023 May 4;14:1150634. doi: 10.3389/fneur.2023.1150634 (PMC10192863; doi:10.3389/fneur.2023.1150634)
Supplement: Supplementary file 1 [file data_sheet_1.zip › Data Sheet 1 - updated/Appendix B2.pdf]

Appendix B2 - Focus group guide - health care providers (English)

Introduction (15 min)

Welcome and practical information

1. Welcome and introduction own background
2. Short explanation about goal meeting and planning.
3. Explain that the whole meeting will be video-recorded → let everybody sign informed consent.
4. Room for questions

Introduction participants

1. Start video recording
2. Ask everyone to introduce him-/herself: name, residence, experience with PD/research.

Introduction of focus group topic

1. Wearable sensors that can capture movements in daily life have been suggested to be helpful in PD care, because they could provide a more objective and continuous picture of a patients performance in real-life.
2. However, we're not there yet: in the survey we found that the paper diary is still the most commonly used tool by both patients and care providers.
3. It is important that new solutions match clinical needs. In this focus group, we are going to address the question: "From your perspective as physical therapist, how can PD care be improved using sensor-based daily life monitoring?"
4. This is a very broad question. Therefore, we will try to answer this question together WITHIN A SPECIFIC AREA OF TREATMENT (for example balance/falls for physical therapists).
5. We will complete a diagram together step-by-step: first we will briefly discuss the main goals of treatment (GOALS), and which tools you already use to collect relevant information. Then, we'll discuss which challenges you come across (CHALLENGES) and which things are already going well (FACILITATORS). Next, we will talk about the advantages that wearable sensors could offer (ADVANTAGES OF WS), and which disadvantages to see (BARRIERS OF WS). We will finalize the discussion by discussing what the ideal tool should look like.

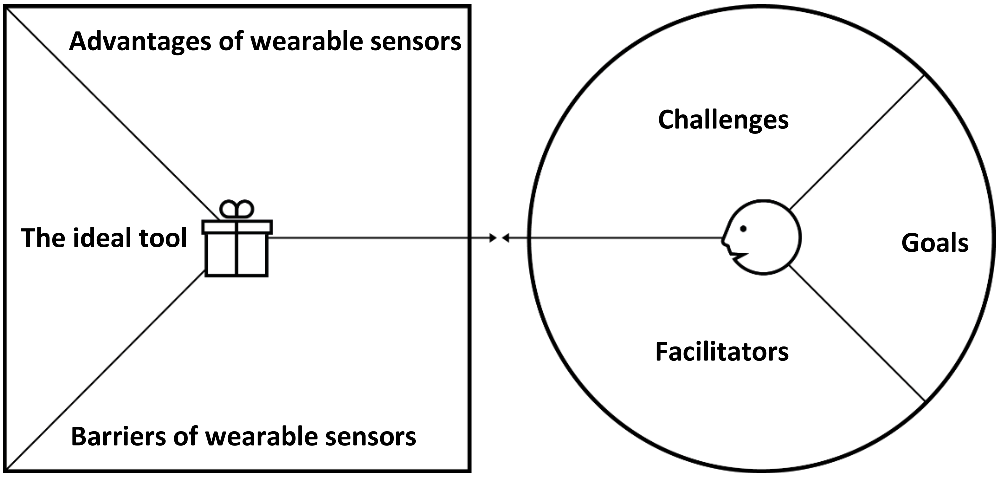

Discussion (45 min)

Instructions

1. Make participants feel comfortable: emphasize that there are no right or wrong answers, interested to hear everyone's thoughts and experiences. Feel free to share both positive and negative feedback. We don't have to come to an agreement about everything, but show respect for each other's opinions.
2. Explain own roles: chairman (because of limited time, I might sometimes steer the conversation a little bit to make sure all topics are covered) and note taker (for later analysis).

Part 1: reach consensus about area of interest (5 min)

1. Using a survey among 86 physical therapists we made a pre-selection of areas that deserve priority for the development of home-based monitoring tools (show selection).
2. Let participants comment: what do they consider the most important area?
3. Reach consensus about domain of interest: this will be the topic of further discussion (place on GOALS)

Part 2: explore current situation within this domain (10 min)

1. Further explore task: what do you as therapists want to achieve with treatment within this domain? (place on GOALS)
2. Which information do you currently collect in relation to this? Do you currently use tools to collect information about the patient's condition in daily life? (e.g. paper diary, smartphone application, website, sensor) How do you use this information? (place on GOALS)
3. Explore facilitators: what is for you the most valuable source of information about the patient's condition? Can you give an example how this information positively influenced the patients' treatment? (place on FACILITATORS)

4. Explore challenges related to this: do you experience any difficulties with using these tools? Is the information you currently collect sufficient to make decisions about the treatment? (can relate to quality of information, lack of information, practical problems, implementation in daily practice, problems due to patient motivation/compliance) (place on CHALLENGES)
5. Ask if the group thinks we covered the most important points of this part of the diagram.

Part 3: how can wearable sensors help in this domain? (25 min)

1. Give a short explanation about wearable sensors: wearable sensors are small devices that can be worn on different body locations, for example as a watch, around the waist or as a strap around the ankle. They can continuously measure movements, and send the information via internet to secured cloud environments. From here, the information could be shared with both patients and care providers. For this discussion, it is important that we want to know what you are INTERESTED in; you don't have to think about whether it is POSSIBLE with wearable sensors.
2. Does anyone already have any experience with using wearable sensors? (other than already mentioned in part 1)
3. Which advantages do you think that wearable sensors can offer in the treatment of [AREA OF INTEREST]. Could wearables provide useful decision support? (can be pain relievers (related to mentioned challenges) or gain creators (benefits unrelated to mentioned problems/new opportunities)). (place on ADVANTAGES)
4. Which problems/challenges do you see related to the use of wearable sensors? (place on BARRIERS)
5. Ask if the group thinks we covered the most important ADVANTAGES and BARRIERS.

BREAK

6. Rank ADVANTAGES and BARRIERS: which advantages and problems do you consider most important?
7. For 2 most important ADVANTAGES → what should the tool look like to have these advantages? (place on THE IDEAL TOOL) Minimal topics to cover:
    a. Which outcome measures should be measured?
    b. Active vs. passive monitoring?
    c. How should information be accessed? (real-time on demand, pro-active signaling based on detection of changes/trends/incidents /only during consultation)
    d. When would you trust measurements?
    e. How should patients be involved?
8. For 2 most important BARRIERS → what should the system look like to overcome these barriers? (place on THE IDEAL TOOL)

Part 4: other questions (5 min)

1. How do you think would this tool influence your relationship with your patients? Do you think it would have an effect on how frequently you would schedule (physical) appointments?
2. How large do you think is the target group for the proposed solution? (both in terms of usefulness and usability)

Closure (5 min)

1. Give a brief summary of important results as is shown by the diagram. Ask if this is a good reflection of the discussion, and give brief room for participants to comment.
2. Thank everyone for contribution. Let participants complete form for reimbursement travel expenses.
3. Make picture with therapists in front of diagram?
4. For those who are interested: short talk about research on wearable sensors.
