# Supplementary material for: Need for personalized monitoring of Parkinson’s disease: the perspectives of patients and specialized healthcare providers
Source: Front Neurol. 2023 May 4;14:1150634. doi: 10.3389/fneur.2023.1150634 (PMC10192863; doi:10.3389/fneur.2023.1150634)
Supplement: Supplementary file 1 [file data_sheet_1.zip › Data Sheet 1 - updated/Appendix C.pdf]

Appendix C - full tables

*Table 1: most frequently mentioned symptoms and factors that influence PD by the different stakeholder groups. *Equal percentages. **Only completed by patients who indicated to be interested in monitoring their PD.*

Symptom	Physiotherapists	Nurses	Neurologists	Early PD	Late PD
Slowness of movement	15%	25%	48%	25%	20%
Tremor	3%	13%	16%	30%	21%
Rigidity (muscle stiffness)	28%	24%	20%	22%	26%
Trouble to start walking or freezing when walking	41%	24%	28%	12%	16%
Problems with walking	45%	11%	20%	17%	22%
Problems with balance and/or falling	71%	35%	24%	15%	21%
Problems with fine motor movements	3%	0%	4%	22%	15%
Problems with speech	0%	4%	4%	13%	12%
Dyskinesia	7%	15%	32%	10%	13%
Dystonia	3%	5%	8%	7%	7%
Pain	9%	2%	0%	8%	9%
Dribbling of saliva	1%	0%	4%	1%	6%
Difficulty swallowing or problems with choking	5%	2%	4%	2%	4%
Loss in your ability to taste or smell	0%	0%	0%	1%	1%
Weight loss (not due to change in diet)	0%	0%	0%	1%	1%
Vomiting or nausea	0%	2%	0%	1%	0%
Bowel problems	0%	5%	0%	5%	9%
A sense of urgency to pass urine	2%	0%	0%	11%	12%
Finding it difficult to have sex when you try	0%	2%	0%	1%	4%
Orthostatic hypotension	10%	13%	12%	5%	3%
Excessive sweating	0%	0%	0%	0%	2%
Sleep problems	10%	18%	16%	20%	11%
Restless legs	2%	0%	0%	8%	8%
Finding it difficult to stay awake during activities	0%	0%	0%	2%	3%
Fatigue or lack of energy	13%	7%	0%	25%	20%
Difficulty concentrating or staying focussed	0%	4%	0%	10%	7%
Problems remembering things	5%	13%	16%	7%	6%
Depressed mood	5%	15%	4%	2%	3%
Hallucinations	2%	16%	12%	1%	3%
Delusions	0%	0%	0%	1%	1%
Impulsive or compulsive behaviour	0%	7%	4%	0%	2%
Double vision	1%	2%	0%	2%	1%
Other	5%	9%	0%	3%	3%

*Table 2: most frequently mentioned symptoms and factors that influence PD by the different stakeholder groups. *Equal percentages. **Only completed by patients who indicated to be interested in monitoring their PD.*

Factor	Physiotherapists	Nurses	Neurologist	Early PD	Late PD
Diet	9%	2%	4%	4%	6%
Parkinson medication	69%	75%	64%	56%	63%
Non-Parkinson medication	1%	0%	4%	2%	6%
Stress	21%	20%	12%	29%	34%
Time of the day	15%	11%	28%	20%	16%
Physical exercise	79%	49%	40%	50%	44%
Hydration	1%	5%	4%	7%	6%
Pain	20%	5%	4%	9%	8%
Allergies	0%	0%	0%	0%	2%
General sense of well-being	24%	27%	48%	13%	13%
Sleep	17%	25%	32%	36%	26%
Weather/season	0%	0%	0%	1%	4%
Mood	16%	27%	16%	13%	10%
Other illness	6%	2%	0%	2%	5%
Social relationships	3%	5%	12%	4%	9%
Other	2%	2%	4%	3%	5%
