# Supplementary material for: Need for personalized monitoring of Parkinson’s disease: the perspectives of patients and specialized healthcare providers
Source: Front Neurol. 2023 May 4;14:1150634. doi: 10.3389/fneur.2023.1150634 (PMC10192863; doi:10.3389/fneur.2023.1150634)
Supplement: Supplementary file 1 [file data_sheet_1.zip › Data Sheet 1 - updated/Appendix D.pdf]

Appendix D – results focus groups and interview

Identified themes and illustrative quotes

Summary of identified themes

	Physiotherapists	Neurologist	Parkinson nurses	Patients
**Focus area**	Management of balance problems and falls	Management of response fluctuations	Supporting self-management	Communication with healthcare providers
**Goals**	- Treatment of balance and falls<br>- Increase awareness of falls and triggering circumstances<br>- Patients can live at home independently	- Insights into response fluctuations<br>- Enable patients to do what they want to do in their daily lives	- Focus on what patients find important<br>- Stimulate patient self-management	- Communicate how I am doing with care providers<br>- Find the right medication schedule<br>- Understand influence of diet<br>- Measure progression over longer time periods<br>- Deal with Parkinson's day by day<br>- Stay active<br>- Collect information for myself
**Gains**	- Home visits provide useful information<br>- A clinical balance assessments sometimes provides insights in different causes of falling<br>- Some patients can adequately describe why they fall<br>- Sometimes the on-off diary provides insights about the role of medication<br>- Filming the patient can increase self-awareness of their walking pattern	- The patient's partner can provide useful information<br>- Based on the anamnesis we can capture complex patients for advanced therapies<br>- Some patients can adequately tell about their response fluctuations<br>- It is helpful if the patient prepares for consultations<br>- Home visits provide useful information	- Home visits provide useful information	- Making notes to prepare for my neurologist's appointment<br>- My partner/friends help to tell my care providers how I'm doing<br>- Thanks to the Parkinson well-being map, my neurologist already knows what is going on<br>- My care providers listen to my story<br>- My physical therapist walks together with me to know how I am doing<br>- I tell my care providers what I would like to discuss<br>- My neurologist is happy with the information I collect<br>- I have a pleasant relationship with my Parkinson nurse<br>- The guidance of my physiotherapist is very helpful<br>- My care providers communicate with each other
**Pains**	- Falls are complex in patients with PD<br>- It is difficult to find the cause of fall incidents<br>- There is a discrepancy between how patients perform in-clinic and at home<br>- Consultations are only a snapshot	- Indication for advanced therapies is difficult<br>- Finding the right medication schedule is difficult<br>- On off diaries are unreliable<br>- Patients often express general complaints / find it difficult to distinguish complaints	- It is difficult for patients to describe the daily patterns of symptoms<br>- Some patients underestimate the duration of sleep<br>- On off diaries are unreliable<br>- Filling out diaries is burdensome for patients	- The Parkinson well-being map is subjective<br>- Filling out diaries takes a lot of time<br>- Keeping track of falls is difficult<br>- I sometimes forget things when I talk to my neurologist<br>- My neurologist only sees how I am doing during the appointment

	- Some patients downplay the severity of fall incidents<br>- On off diaries are unreliable<br>- It is difficult to assess why cognitive strategies are not always working<br>- Applying learnt strategies at home is challenging for cognitive impaired patients	- It is difficult to derive the pattern of response fluctuations from the patient's story<br>- There is a discrepancy between how patients perform in-clinic and at home<br>- Sometimes there is a discrepancy between the story of the patient and the partner<br>- Consultations are only a snapshot<br>- The UPDRS is not suitable to track long-term changes<br>- Patients need to take an extra dose in time when they are off<br>- Not many patients take the lead in what they want to talk about	- Filling out diaries can become an obsession for some patients<br>- Some patients find it difficult to accept the diagnosis<br>- It is difficult to evaluate patients with cognitive problems and without partner	- My neurologist does not see the value of the Parkinson well-being map<br>- My neurologist has little time<br>- My general practitioner knows little about PD<br>- More information about PD is confronting<br>- I do not know which PD app is good<br>- Finding the right medication schedule takes a long time<br>- There is no pattern in my PD symptoms<br>- It is difficult to anticipate<br>- It is difficult to distinguish between PD and general complaints<br>- It took a long time before the diagnosis became clear
**Advantages of wearables**	- Insights in what precedes a fall at home<br>- Give more personalized treatment to prevent falls<br>- Increase self-awareness of patients<br>- Increase treatment compliance<br>- Device warns the patients in situations with high fall risk<br>- Device reminds the patients of learnt movement strategies<br>- Direct help with fall incidents<br>- Positive feedback with doing exercises<br>- More standardized treatment guidelines<br>- Collect evidence for value of physiotherapy<br>- Signaling problems that need attention<br>- Added value for specific target group	- Discuss patterns together with patient<br>- More time for the conversation with the patient<br>- Give realistic expectation what can be achieved with medication<br>- Adjust medication schedule more efficiently<br>- Digital on off diary<br>- Quickly see whether treatment is effective<br>- Continuous measurements<br>- Rule out that discomfort is caused by motor symptoms<br>- Support decision for advanced therapy<br>- Increase self-awareness of patients<br>- Insights in when the patient actually takes his medication<br>- Benefit for closed-loop therapies<br>- Makes conversation with patient more focused<br>- Supports the patient to tell his story<br>- Telemedicine<br>- Added value for specific target group	- Increase self-awareness of patients<br>- Disentangle what is related to PD and what is not<br>- Supports the patient to tell his story<br>- Provides something concrete to discuss with patients<br>- More time for conversation with the patient<br>- Give targeted advice on how patients can prevent falls themselves<br>- Increase treatment compliance<br>- Support acceptation of the diagnosis<br>- Stimulate inactive patients to become more active<br>- Coach to maintain healthy walking pattern<br>- Early signaling of problems that need medical attention<br>- Give the patient a feeling of safety<br>- It is easier for patients<br>- Obtain more insights into what happens at night<br>- Obtain insight into fall incidents	- Support care providers to find optimal therapy<br>- Adjust medication schedule more efficiently<br>- Support setting up duodopa pump treatment<br>- Targeted advice on which exercise I can do<br>- Less frequent hospital visits<br>- Care providers are more prepared for appointments<br>- Share measurements with my physical therapist<br>- Early signaling of problems that need medical attention<br>- Obtain an overview of the week<br>- Disentangle what is related to PD and what is not<br>- Obtain more insights into what happens at night<br>- I would trust the sensor measurements more than my own subjective impression<br>- Predict when I will fall<br>- Predict when I will have freezing of gait<br>- Coach to maintain healthy walking pattern<br>- Optimize when I eat and when I take my medication<br>- Predict how the disease will develop<br>- Obtain insights into progression over long time periods

		- Early signaling of problems that need medical attention	- Support setting up duodopa pump treatment	- Focus on different things than tracking<br>- Support acceptation of the diagnosis
**Limitations of wearables**	- It may be too difficult for patients<br>- Difficult to measure non-motor symptoms, which are important for patients<br>- Verbal coaching by a device may not be sufficient to instruct some patients<br>- Proactive signaling may have little added value: patients need to have a request for help<br>- Time investment and costs<br>- Patient privacy	- The severity of symptoms does not tell the whole story<br>- Distinguish between pure motor symptoms and loss of initiative<br>- More information may raise more questions<br>- Subjective experiences are more important<br>- Distinguish dyskinesias from intentional movements may be technically difficult<br>- In an earlier pilot with a sensor, the added value was limited<br>- Range of well controlled symptoms may be difficult to determine<br>- Time investment and costs<br>- Needs to match with the patient's coping style<br>- Patient privacy<br>- If the patient is aware that he is being measured, this may introduce bias	- Subjective experiences are more important<br>- Too much focus on numbers<br>- No replacement for face-to-face contact<br>- Difficult to measure non-motor symptoms, which are important for patients<br>- A coaching device may scare some patients<br>- It may be too difficult for patients<br>- More information may raise more questions<br>- Time investment and costs	- It can become an obsession for patients<br>- No replacement for face-to-face contact<br>- Proactive signaling may have little added value: patients need to have a request for help<br>- Time investment and costs<br>- The collected information does not change anything<br>- Limited reliability of measurements<br>- Patient privacy
**Ideal tool**	- Excellent usability<br>- Clear agreements on use of sensors<br>- Preference for passive over active registrations<br>- Specific suggestions for what to measure	- Preference for passive over active registrations<br>- Combination of objective and subjective information<br>- Coordination by Parkinson nurse<br>- Measure for at least one week<br>- Focus on changes within person<br>- Focus on a few important parameters<br>- Important to discuss expectations<br>- Excellent usability<br>- Easy to interpret quickly<br>- Endorsement by non-commercial parties important to build trust<br>- Validation is important<br>- Need to be able to rely on unexpected findings	- Targeted use for specific patients<br>- Access to data during consultations<br>- Clear agreements on use of sensors<br>- Combination of objective and subjective information<br>- Focus on changes within person<br>- Validation is important<br>- Trust in measurement when the patient recognizes it<br>- Personalized coaching<br>- Balance between too little and too much stimulation<br>- Include a rewarding system<br>- Signaling function in first place directed to caregiver	- Data should always be accessible to care providers<br>- I would like to personally hand over the data to my care providers<br>- Care providers may proactively reach out to me<br>- Access to data for patients<br>- I want access to a clear summary<br>- Targeted use for specific patients<br>- Coordination by Parkinson nurse<br>- Able to measure for 24 hours per day<br>- Does not interrupt sleep<br>- Accurate information about what the sensor can measure is important<br>- Preference for passive over active registrations<br>- Smart timing of active tasks<br>- Repeating the same active tasks becomes boring<br>- Trust in measurement when the patient recognizes it

		- It needs to be transparent how outcomes were derived<br>- Own experience is important to build confidence in the measurements<br>- Access to data during consultations<br>- Access to data for patients		

Physiotherapists: treatment of falls and balance

Theme	Illustrative quote
**Goals**	
Treatment of balance and falls	"Ik denk dat er veel gewonnen kan worden op het gebied van balans en vallen. De zorgkosten nemen natuurlijk ook enorm toe als patiënten veel beginnen te vallen."
Increase awareness of falls and triggering circumstances	"Zo lang mensen zich bewust zijn van wanneer ze vallen, en dat ze soms vallen, vallen ze veel minder vaak."
Patients can live at home independently	"Het is ook een indicatie dat mensen zelfstandig in hun eigen huis kunnen blijven wonen"
**Gains**	
Home visits provide useful information	"Als je dan over valproblematiek praat in de praktijk, dan is denk ik de stap die je dan moet maken naar de thuissituatie gaan om daar te kijken en dat je dan pas ziet. In een oefenzaal kun je dingen nabootsen, maar waar het om gaat is dat het thuis wordt nagebootst."
A clinical balance assessments sometimes provides insights in different causes of falling	"Wat ik zie waar mensen wel of niet vallen, ik voel me ontzettend geholpen door het nieuwe meetinstrument de mini best test uit de Nieuwe Richtlijn die in het Nederlands vertaald is. Ik zie daar namelijk in op welk domein mensen uitvallen. Of het sensorische oriëntatie is, of het hun opvangreacties zijn, of die adequaat of niet adequaat zijn. Ze kunnen vertraagd zijn, maar dan nog al of niet adequaat. Hoe hun loopvaardigheid is en daaruit haal ik tegenwoordig veel meer gegevens waar hun balans en valrisico's problemen opleveren."
Some patients can adequately describe why they fall	"Ik heb een mevrouw gehad die viel 2, 3 keer in de week en dat was altijd bij het planten water geven. Dan is het heel makkelijk. Dan zet je ze gewoon op een rollator en zeg je: ga zittend uw plantje water geven. Dan is het probleem opgelost."
Sometimes the on-off diary provides insights about the role of medication	"We houden wel eens bij, we hebben ook wel eens een soort Meerwaldtkaart gebruikt om te kijken hoe mensen in de medicatie zaten. Om te kijken wanneer ze vonden dat ze goed bewogen en welke uren van de dag ze minder goed bewogen. Als er echt iets uitkomt dat je zegt: je bent 4 keer gevallen in een maand tijd en het was altijd rond dat tijdstip, dan kun je daar iets mee."

Filming the patient can increase self-awareness of their walking pattern	"Ik geef mensen wel eens feedback op hun looppatroon, niet alleen Parkinson overigens, door de filmen, want met de telefoon kun je tegenwoordig gemakkelijk filmen. Dat doe ik wel op mijn eigen telefoon."
**Pains**	
Falls are complex in patients with PD	"Bij de Parkinson heb je en je balans en je hebt je omgeving en je hebt je hulpmiddelen die wel of niet gebruikt worden. Het is veel complexer."
It is difficult to find the cause of fall incidents	"Maar je hebt mensen die vallen 3 keer in de week of 3 keer per dag. Als je daar niet bij bent, er moet iets zijn waardoor ze vallen. Dat kan tensiedaling zijn, medicatie, maar die losliggende snoertjes zijn al lang weg, dus dat is het niet hopen wij. Dus er moet wel een constante zijn waarom ze vallen en dat weten wij niet altijd."
There is a discrepancy between how patients perform in-clinic and at home	"Op de balansbord doen ze het helemaal niet slecht, kracht is best goed, opvangreactie is goed en toch vallen ze 3 keer in de week. Dat is wel frustrerend."
Consultations are only a snapshot	"En dat doen we eigenlijk nu vooral als de patiënt in je kamer, de spreekkamer is, maar dat is maar een heel kort moment hè. Dat is soms maar een kwartier of 20 minuten voor een controle. En dan is dat altijd een momentopname."
Some patients downplay the severity of fall incidents	"Als ik zie: die valt bijna, dat kan voor een patiënt zelf de indruk hebben: het valt allemaal best wel mee."
On off diaries are unreliable	Als je bijvoorbeeld een Meerwaldtkaart wil invullen, ik zie vaak bij de mensen waar ik dat heel graag wil, die zijn er niet toe in staat. Dan ben je afhankelijk van bijvoorbeeld een verzorgende die dat doet, maar die hebben wel meer te doen, dan krijg je weer een halve kaart en dan denk ik: daar heb je niks aan.
It is difficult to assess why cognitive strategies are not always working	"Je hoort dus een verhaal dat er een verschil is in responsiviteit op een cognitieve strategie. Dat kan door daling van medicatiespiegel 's nachts zijn, door vermoeidheid 's nachts, slaperig, maar ook door cognitieve verandering, desnoods dubbeltaken. Het is heel moeilijk om dat allemaal te ondervangen."
Applying learnt strategies at home is challenging for cognitive impaired patients	Maar als je een simpele oefening doet met een opstapje en dat wil herhalen, na 2 keer opstappen weten ze niet meer wat ze aan het doen is en dan gaat dat wel door mee te doen, door het steeds te spiegelen, maar dat zijn hele lastige situaties waar je echt met de cognitie te maken hebt.
**Expected advantages of wearables sensors**	
Insights in what precedes a fall at home	Dan zou het fijn zijn als we op een gegeven moment vanuit de techniek gaan herkennen wat de prefactoren zijn wanneer iemand gaat vallen, of er gewankeld wordt of juist gebukt of snel bewogen of snel gedraaid. Ik weet niet of er variabelen zijn die net dat stukje voordat die val daadwerkelijk plaatsvindt.
Give more personalized treatment to prevent falls	Je zou een soort risicogroep analyse ervan kunnen maken, dat je meer zicht krijgt in de factoren die dat vallen veroorzaken, gekoppeld aan onze testen die we al doen. Als je dat een geheel kunt maken met die sensorische informatie, dan denk ik dat je een profiel kunt maken waarop je weer de mogelijkheden van de verbale instructies bij bepaalde situaties.

Increase self-awareness of patients	Ik zou tientallen mensen kunnen noemen waarvan ik denk: ja dat is eigenlijk wel mooi om inzichtelijk te maken: wat is er nou gebeurd en daar nog eens op terugkomen.
Increase treatment compliance	"Dan probeer ik bij alles heel goed aan te geven wat de relevantie is voor die persoon en waarom die er iets mee moet gaan doen op dit moment of waarom dat nu nog niet hoeft, maar waar we op blijven letten, zodat je wel weet: je moet iemand zien te motiveren om open te staan voor zorg, voor hulp"
Device warns the patients in situations with high fall risk	De vraag is of dat vallen, als er een cognitieve factor meespeelt, met selectieve aandacht te maken heeft en als het met selectieve aandacht te maken heeft en de sensor kan de voorbewegingen van een val op een gegeven moment gaan herkennen, dan kan die met een waarschuwingssignaal iemand zo alert maken dat die misschien toch het goede besluit neemt of zegt: pas op de plaats.
Device reminds the patients of learnt movement strategies	Ik kan me ook voorstellen in sommige situaties als je een sensor hebt die even verbaal laat horen: let op, grote stappen, blijf stappen, net even die reminder voor een stoel waar hij altijd gaat zitten, blijf die stappen maken, net als dat je in je trainingssituatie doet.
Direct help with fall incidents	Mensen hebben al wel zo'n belletje als ze vallen en dan heb je een soort alarmlijn. Hoe mooi zou het zijn als dat ding signaleerde: het is echt mis en de cliënt respondeert niet door iets uit te zetten of zo na een minuut of twee. Waarschuw iemand. Dat zou toch ook mooi zijn.
Positive feedback with doing exercises	Een stukje balanstraining wat ze thuis moeten uitvoeren denk ik dat wel motiverend kan zijn, als ze bijvoorbeeld met de voeten voor mekaar moeten blijven staan en ze krijgen een signaal als het goed gaat. Het wordt gemeten en: 'je hebt 15 seconden gehaald' of zo, dat zal zeker motiverend werken.
More standardized treatment guidelines	Ik ben het zeer met xxx eens, als je hele goede meetinstrumenten hebt en een goede dataset al op maat maakt voor die patiënt, dan weet je, iedereen werkt volgens die manier, wie je op welke manier moet aanpakken.
Collect evidence for value of physiotherapy	Toen werd er gezegd: 'er is gewoon niet meer geld beschikbaar voor de fysiotherapie', maar toen zei hij op een gegeven moment: 'als jullie nou eens kunnen aantonen, als voorbeeld de Parkinsonpatiënt, met harde data wat jullie functie is binnen dat valgebeuren, dat jullie als fysiotherapeuten ervoor zorgen dat mensen minder vallen, minder vaak opnames in het ziekenhuis voor breuken, dan gaat er vanuit de tweede lijn, vanuit het ziekenhuis geld naar jullie.
Signaling problems that need attention	We hebben het er wel eens over gehad dat de mensen vaak wat apathischer worden en initiatiefloos en dan daar zelf niet meer mee komen. Het is toch wel onze taak om dan ook dingen te kunnen signaleren en daar de voorlichting in te geven van: dit zouden we kunnen doen, dit zouden we voor jullie kunnen betekenen.
Added value for specific target group	Cognitieve stoornissen, geen partner, een positieve valgeschiedenis, het laatste half jaar meerdere keren gevallen.
**Expected barriers / disadvantages of wearables sensors**	
It may be too difficult for patients	kunnen mensen daar goed mee omgaan? Ze moeten opgeladen worden, ze moeten 's morgens aangebracht worden, 's avonds weer weggelegd. Dat praktische aspect er omheen is vaak een beperking in het gebruik van die sensoren.

Difficult to measure non-motor symptoms, which are important for patients	We zijn eigenlijk al aangeland op een discussie, we hebben het continu over cognitieve problemen. Die sensoren gaan in op beweging, dus daar heb je al de wrijving.
Verbal coaching by a device may not be sufficient to instruct some patients	Soms wel, maar als ik me dan bedenk, op het moment dat je zo'n oefening bij iemand aan het doen bent en je vertelt wat er moet gebeuren, het komt gewoon niet binnen. Je moet dan al vertellen en visueel voordoen en dan komt er iets.
Proactive signaling may have little added value: patients need to have a request for help	In principe als iemand geen zorgvraag heeft, we kunnen moeilijk een zorgvraag opdringen.
Time investment and costs	Dat je toch weer een test moet aflezen of informatie moet opslaan, informatie moet uitlezen met de patiënt. Wij moeten al heel veel handelingen verrichten.
Patient privacy	Dus niet dat ik kan zeggen: ik ga eens even kijken hoe het met meneer Jansen is vanavond, of hij op de bank zit of dat hij aan het oefenen is. Dat vind ik te ver gaan.
**Ideal tool**	
Excellent usability	Zo simpel mogelijk, dat er bijna geen omkijken naar is.
Clear agreements on use of sensors	Ik denk ook dat er informed afspraken over hoe lang of wanneer.
Preference for passive over active registrations	Anders is de focus op de oefening en niet op de omgeving waarom die opstaat. Hij staat om een reden op, niet om die test te doen, maar hij wil naar het toilet. Dat meet die dan heel natuurlijk, maar anders zeg je: 'je moet opstaan'. Dan ga je, zeker als je het cognitief een beetje door hebt, extra je best doen.<br><br>Het kan wel iets opleveren op het moment dat je zegt: ik vind dat een belangrijke oefening voor die persoon om dat regelmatig te herhalen. Dan haal je daar zeker wat uit.
Specific suggestions for what to measure	De balans in stand. Als ze in de keuken staan of bij de wc en broek moeten ophijsen en vastmaken.

Neurologists: treatment of response fluctuations

Code	Illustrative quote
**Goals**	
Insights into response fluctuations	ik kan mij in die zin wel vinden in dat doel vanuit neurologisch oogpunt gezien is inderdaad die responsfluctuaties is want dat is natuurlijk waar ook onze behandeling op gericht is, meer dan wat de fysiotherapeuten hebben. Kijk, vallen is in die zin voor ons ook wel belangrijk, maar daarvoor sturen we mensen dan weer juist naar de fysiotherapeut, dus daar interveniëren wij weinig in, maar die responsfluctuaties dat is natuurlijk ook iets wat echt bij de neuroloog ligt hè, van wat doe je met die medicatie en hoe vang je dat op?
Enable patients to do what they want to do in their daily lives	En uiteindelijk is dat toch waar het om gaat. Wat ik aan patiënten ook uitleg is hè je kijkt ze natuurlijk ook in de spreekkamer af en toe na. Maar ik geef die medicatie niet om te zorgen dat ze in de spreekkamer zo snel mogelijk dit kunnen doen. Ik geef het om te

	zorgen dat ze in hun dagelijks leven gewoon die dingen kunnen doen die voor hen belangrijk zijn. En of ze dan heel snel met hun vingers kunnen tappen is dan nogal secundair.
**Gains**	
The patient's partner can provide useful information	Eigenlijk moet je een gesprek alleen hebben hè. Een gesprek alleen met de partner.
Based on the anamnesis we can capture complex patients for advanced therapies	En aan de andere kant op het moment dat het ons blijkbaar lukt om op basis van anamnese al ja zeg maar de mensen die complex zijn te vangen, dan is de vraag een beetje wat dan meerwaarde van je tool is.
Some patients can adequately tell about their response fluctuations	Een deel van de patiënten kan ook gewoon prima vertellen dat ze gewoon wat wearing off hebben. Gewoon heel goed kunnen vertellen van nou een half uurtje voor mijn nieuwe pil merk ik dat mijn tremor terug komt en als ik een nieuwe pil neem dat is hij naar een kwartiertje/ half uurtje weer verdwenen.
It is helpful if the patient prepares for consultations	dat de mensen wel steeds bewuster voor worden van het feit van hè, bereid je bezoek aan de dokter voor. Hè, dat als je, dat je goed beslagen ten ijs komt. Ook als patiënt dat je zelf ook weet van goh, wat wil ik in dat gesprek aan de orde brengen. Hè, en dat je als het ware ook de patiënt een beetje in de lead laat over van wat hij wil bespreken in plaats van dat je dat als dokter alle informatie die jij wil weten boven tafel haalt hè.
Home visits provide useful information	Soms gaan verpleegkundigen op huisbezoek. Dat is informatief om te achterhalen hoe het thuis met iemand gaat.
**Pains**	
Indication for advanced therapies is difficult	Ja is al lastig. Dus dat klopt. En dan stuur ik ze vaak toch een keer door naar Nijmegen. En dan vinden zij het soms ook wel moeilijk. Dan gaan ze het toch nog weer opnieuw proberen met medicijnen. En wat heel lastig is, je probeert een goed beeld te krijgen. En wat ze dan nu vaak doen is een opname in de kliniek.
Finding the right medication schedule is difficult	Bij veel mensen is dat een uitdaging.
On off diaries are unreliable	Kijk, de patiënten die het goed kunnen vertellen die kunnen ook goed zo'n kaart invullen hè, het is vooral de mensen die het zelf moeilijk vinden om het te vertellen, of omdat er totaal geen patroon in zit, ja, die hebben vaak ook moeite om die kaart wel in te vullen. Omdat heel veel mensen hè, als die cognitief goed zijn kunnen zelf ook heel goed zeggen waar de problemen natuurlijk liggen.
Patients often express general complaints / find it difficult to distinguish complaints	Want ze zeggen vaak het gaat niet goed dokter en dan moet je gaan puzzelen van waarom gaat het niet goed? is het puur motorisch gedeelte? En als dat motorisch is, is het dan stijfheid of over bewegelijkheid of tremor?
It is difficult to derive the pattern of response fluctuations from the patient's story	Het moment dat je een patiënt vraagt van beschrijf eens hoe een dag eruit ziet, ja dan is het heel moeilijk om nou precies te snappen van is dit nou wearing off? Is er een delayed on? Zit er voorspelbaarheid in? Zit er een patroon in?
There is a discrepancy between how patients perform in-clinic and at home	Want de situatie hier in de spreekkamer is altijd anders dan dat hij inderdaad thuis is, dus je vaart op wat mensen thuis ervaren.

Sometimes there is a discrepancy between the story of the patient and the partner	En dan zeggen die patiënten vaak goed of niet goed of dan hoor je iets anders van de partner.
Consultations are only a snapshot	En dat doen we eigenlijk nu vooral als de patiënt in je kamer, de spreekkamer is, maar dat is maar een heel kort moment hè. Dat is soms maar een kwartier of 20 minuten voor een controle. En dan is dat altijd een momentopname.
The UPDRS is not suitable to track long-term changes	En op dit moment met de UPDS dan heb je licht, matig, ernstig bij wijze van spreken. En op het moment dat ik een arm voel met rigiditeit en ik schrijf op licht en de keer daarop schrijf ik op matig. Dan weet ik niet helemaal precies of ik nou hetzelfde bedoel. Want daar zit 3 maanden tussen of een half jaar tussen.
Patients need to take an extra dose in time when they are off	En moeten ze het liefste-- Instrueren we ze ook altijd hè, dat op het moment dat je off raakt moet je op de extra dosis drukken, maar je moet te lang wachten. Als je te diep off bent dan is die extra dosis misschien niet goed genoeg.
Not many patients take the lead in what they want to talk about	ik moet nog zeggen dat er maar weinig patiënten zijn die daadwerkelijk met, ze hebben natuurlijk altijd allemaal vragen naar aanleiding van uitslagen of iets of wat dan ook, maar er zijn weinig patiënten die ook zelf al het gesprek beginnen met dokter, vandaag wil ik het hier en hier en hier eens even over hebben.
**Expected advantages of wearables sensors**	
Discuss patterns together with patient	En dan heb je een inzicht. En dan overleg je met de patiënt denkt u dat dit zo is? De app geeft dit of dat aan. En dan kun je vragen bent u het daar mee eens? En dan vraag ik altijd bent u tevreden met hoe het nu gaat?
More time for the conversation with the patient	Ja het scheelt misschien in die zin tijd dat je daardoor juist meer tijd kan besteden aan het gesprek.
Give realistic expectation what can be achieved with medication	Dus dat mensen zeggen ik voel me toch rot. Ik voel me niet gelukkig. En dat ze toch ervan uit gaan dat dat met medicatie allemaal opgelost kan worden.
Adjust medication schedule more efficiently	Dus dat je inderdaad sneller de juiste dosering medicatie kan geven, waardoor je dat proces van instellen van medicatie sneller kunt doorlopen.
Digital on off diary	Van is die patiënt nou de hele dag off? Dus is die hele tijd stijf? Trilt hij heel veel? Trilt hij aan beide lichaamshelften of juist met 1 lichaamshelft? Of in een wat gevorderder stadium, is die patiënt juist onbeweeglijk? En als je sensoren hebt die zo goed zijn die daar onderscheid in kunnen maken, dan heb je eigenlijk een perfecte meerwaldtkaart.
Quickly see whether treatment is effective	Ja dan kan je in 1 oogopslag zien of je behandeling aanslaat of niet.
Continuous measurements	Ja. Maar het voordeel van goede sensoren is dat je een 24-uurs beeld krijgt. En ook ’s nachts, dat is ook gaaf als dat kan.
Rule out that discomfort is caused by motor symptoms	Maar het geeft dus wel heel mooi een weergave van hoe het motorisch met die patiënten gaat, want als je ziet dat het motorisch redelijk goed gaat dan weet je dat er iets anders speelt. Ik denk dat dat een grote winst is.

Support decision for advanced therapy	En dat je dan het stadium van het medicijn werkt echt niet meer, dat je dat sneller, dat je dat naar voren haalt. En dat je daardoor met mensen misschien eerder voor advanced therapie in aanmerking laat komen.
Increase self-awareness of patients	je kan een patiënt meer inzicht geven in zijn eigen functioneren misschien als je het wat langer kan monitoren dan dat je het er even in de spreekkamer over hebt.
Insights in when the patient actually takes his medication	Nou, wel registreert ja, ja, dat is denk ik wel handig, dat je vooral goed kan zien op welk moment neemt de patiënt welke pillen? Want als je iets aan die pillen wil doen hè, aan het aanpassen van de medicatie, dan heb je juist heel goed inzicht nodig op wanneer neemt de patiënt nou daadwerkelijk zijn pillen? Hè, want misschien maakt die daar wel een potje van.
Benefit for closed-loop therapies	Van hoe kan je nou zogen voor adaptieve DBS? Op het moment dat je off bent dat je stimulatie parameters aanpast en op dat moment hè-- Die closed loop kan je doen door in de hersenen zelf te meten en te kijken of er veranderingen zijn. Maar je kan het ook doen door met activity monitoring te kijken wanneer de patiënt off is.
Makes conversation with patient more focused	Nou, het maakt het gesprek veel concreter. Hè, omdat je heel actueel op de huidige problemen van zo'n patiënt in kan gaan. Kijk, want een Parkinsonpatiënt heeft natuurlijk verschillende problemen hè, en de ene keer is het ene probleem van de off-periodes misschien groter en de andere keer hebben ze misschien meer problemen over van de obstipatie en de incontinentie of dat soort dingen hè, of komen de cognitieve problemen meer op de voorgrond of het vallen. Hè, dus ik denk dat je de inhoud van je gesprek veel doelgerichter maakt.
Supports the patient to tell his story	Dat dat een voordeel kan zijn. Kijk, je kunt aan een patiënt vragen goh, van wat, hoe gaat het? Hè, of van waar wil je het vandaag over hebben hè, van wat is op dit moment je grootste probleem. En dat kun je doen op basis van wat de patiënt zegt maar dat kun je natuurlijk ook ophangen aan wat je gezien hebt, zo van dit en dit hebben we gezien.
Telemedicine	Gewoon zorg op afstand is het eigenlijk een beetje hè, dus meer in de thuissituatie om zoveel mogelijk de zorg buiten de muren ook van het ziekenhuis te houden.
Added value for specific target group	maar op de lange termijn denk ik dat je het vooral toe gaat passen voor wat ik al zei hè, de kwetsbare patiënt waar weinig mantelzorg is of die cognitief niet helemaal meer goed zijn dat je denkt goh, ik krijg geen goed beeld van het functioneren van zo'n patiënt thuis, en als je inderdaad twijfelt over medicatie of over de activiteiten van zo'n patiënt.
Early signaling of problems that need medical attention	Nou ja, k denk het wel dat je het ook kan gebruiken voor inderdaad het signaleren van vroegtijdige problemen hè, als je bepaalde randvoorwaarden afspreekt hè, en wij doen dat, voor de Parkinsonpatiënten is dat nog een beetje lastig van waar moet dan die grens liggen hè, maar dat jij vroegtijdig een signaaltje krijgt van hé die patiënt valt buiten de range hè, die moet eerder op controle komen bijvoorbeeld of daar moet de huisarts echt even gaan kijken of de Parkinsonverpleegkundige uit de regio hè, dat die thuis gaat kijken bij zo'n patiënt, hè, om problemen misschien voor te zijn, hè,

	valproblemen of verwardheid, delier, noem maar iets wat daarbij voorkomt. Hè, we doen dat hier in het ziekenhuis wordt dat heel actief gedaan met de COPD-patiënten hè, die vullen vragenlijsten in en hè, die wordt continu eigenlijk gemonitord en dat op het moment dat zo'n patiënt buiten eigenlijk in het rode of oranje rode gebied komt, ja, dan wordt er al geïntervenieerd en dat leidt tot veel minder exacerbaties van die COPD en veel minder opnames. Ja, en zo moet je dat denk ik bij een Parkinsonpatiënt ook zien hè, dat je bepaalde problemen ook voor kan zijn.
**Expected barriers / disadvantages of wearables sensors**	
The severity of symptoms does not tell the whole story	Want dat is heel lastig. Want er zijn mensen die tremoren hebben en die toch nog wel kunnen werken. En andere mensen met dezelfde tremor die niet meer kunnen werken. Dus dat is ook persoonsgebonden. Hoe onhandig is iemand? Wat voor doorzettingsvermogen heeft iemand? En wat voor eisen stelt iemand aan zijn eigen functioneren?
Distinguish between pure motor symptoms and loss of initiative	Want dat niet bewegen is ook vooral een psychologisch dingen hè. Dat mensen namelijk veel meer initiatieflozer zijn. En dat de partners steeds queues moet geven van nu moet je dat doen en nu moet je dat doen.
More information may raise more questions	Ja dat roept dan weer vragen op, dan zeggen ze in de app zie ik dit en dat en dat. Wilt u niet de medicijnen nog verder ophogen?
Subjective experiences are more important	Een onderbehandeling van wearing off te attaqueren. Ik vraag me af hoe groot dat probleem is weet je. Op het moment dat de patiënt dat niet als hinderlijk ervaart. Je een monitor nodig hebt om dat te vangen, dan is ook de vraag of je daar een interventie op moet toepassen hè.
Distinguish dyskinesias from intentional movements may be technically difficult	Dyskinesieën meten was vroeger lastig. Dyskinesieën lijken op gewone motoriek. In essentie is het dezelfde generator, alleen zit er geen bedoeling achter. Alleen met je versnellingsmetertjes kan je die doeling niet zien.
In an earlier pilot with a sensor, the added value was limited	Laten we zeggen het aantal keren dat hij me echt hielp was relatief beperkt.
Range of well controlled symptoms may be difficult to determine	kun je vroegtijdig signaleren van problemen als je bepaalde marges aan wilt houden, ja, dat is bij een Parkinsonpopulatie natuurlijk heel lastig omdat het zo'n divers ziektebeeld is hè, zoals bij de COPD-patiënt is dat denk ik veel makkelijker hè, zoals we dat hier dan doen veel makkelijker om die randvoorwaarden goed te stellen.
Time investment and costs	Gebruiksgemak en tijd inderdaad hè. Ik bedoel dit is-- Een spreekuur dat, op dit moment is dat een kwartier voor een controle. En als je dan 10 patiënten met Parkinson achter elkaar hebt dan is dat echt gewoon kilhard doorwerken.
Needs to match with the patient's coping style	Sommige patiënten zijn ook veel meer geïnteresseerd in techniek hè. Sommige mensen zijn ook veel meer geobsedeerd door hun eigen ziekte hè, dus die zijn daar ook al veel meer mee bezig en sommige mensen die zitten dan nog in die ontkennende fase, ja, die willen er natuurlijk helemaal niks mee te maken hebben.
Patient privacy	Nou, het is een beetje big brother is watching you hè, dat kan een patiënt denk ik wel als vervelend ervaren. Het hangt er een beetje van af hoe, hè, hoe je het meet. Je bent al patiënt en als je dan ook

	nog op wat voor manier continu gemonitord wordt, ik kan mij voorstellen dat een patiënt dat ook niet leuk vindt
If the patient is aware that he is being measured, this may introduce bias	Nadeel is dat de patiënt zich er bewust van is dat hij/zij een sensor raakt en dat hij/zij zich anders gaat gedragen. Bijvoorbeeld hij/zij is zich meer bewust bij het lopen en dan gaat het beter. Dat geeft een vertekend beeld.
**Ideal tool**	
Preference for passive over active registrations	Weet je uiteindelijk ga je dan, ja, toch een soort kunstmatige meting doen. Dat is niet waar het echt om gaat. Het blijven momentopnames.
Combination of objective and subjective information	Het belangrijkste is dat patiënten via een eenvoudige knop van tijd tot tijd kunnen aangeven hoe ze zich voelen, bijvoorbeeld of ze zich prettig voelen, ellendig, gestrest (emotie) etc. Dat is belangrijk voor de interpretatie van de data. De combinatie van subjectieve en objectieve gegevens is belangrijk.
Coordination by Parkinson nurse	Nee, ik denk dat de monitoring an sich hè en het signaleren van problemen, dat dat prima via een verpleegkundige kan, hè, en dat die verpleegkundige dan op het moment dat iemand buiten de, hè, die marges valt overlegt met de neuroloog van goh, ik signaleer dit en dit hè, wat zullen we doen?
Measure for at least one week	Ik denk dat je wel minstens een week dat moet doen, ja. Omdat het zo wisselt. Kijk, als je het maar twee, drie dagen doet dan kan het heel goed zijn, dan heb je eigenlijk nog helemaal geen goed beeld van het hele functioneren van zo'n Parkinsonpatiënt.
Focus on changes within person	Ik denk dat je vooral ook moet kijken van wat verandert er in het patroon van een patiënt? Dat je dan een bepaalde knik ziet of zo.
Focus on a few important parameters	Maar ik denk het definiëren van wat je wilt meten, en ik denk ook dat je het niet te moeilijk moet maken. Ik denk dat je maar een paar parameters moet meten, dus bijvoorbeeld hè, bij Parkinson dus tremor en ja, traagheid denk ik, stijfheid is bijna niet te meten denk ik.
Important to discuss expectations	Maar je moet dan goed uitleggen dat als ze zien hè dat ze bijvoorbeeld veel trillen als de app dat weergeeft, dat het niet meteen betekent dat je direct kunt behandelen.
Excellent usability	Het zou gebruiksvriendelijk zijn. Patiënten moeten er geen last hebben tijdens het dragen en moet gemakkelijk te bedienen zijn. Hebben een oudere patiëntenpopulatie en patiënten moeten er niet gestrest door raken.
Easy to interpret quickly	Eigenlijk moet je in één ding kunnen zien, want dat doet een Meerwaldt-kaart ook, je kan in één A4'tje kan je zien wat er aan de hand is en dat zou je dus willen.
Endorsement by non-commercial parties important to build trust	Ik heb daar natuurlijk alleen maar vertrouwen in als ik weet dat er ook mensen achter zitten die-- Ja de centra die het hebben ontwikkeld.
Validation is important	maar weet je, dan wil ik gewoon ook echt iets wat goed onderbouwd is, wat wetenschappelijk onderzocht is.
Need to be able to rely on unexpected findings	En aan de andere kant op momenten dat het helemaal 1 op 1 matcht met wat ik zelf al dacht, dan is het de meerwaarde natuurlijk 0. Want dan wist ik het al.

It needs to be transparent how outcomes were derived	wat ik daar lastig aan vind ook is dat je daar niet onder de motorkap kan kijken hè. Je krijgt zo'n soort rapport vanuit de zwarte doos met soms een expert die er ook nog wat van vindt. Wat niet helemaal past bij-- Ja niet transparant maar ook op het moment dat dat niet lijkt de passen bij wat je eigenlijk van die patiënt denkt, dan kan je ook niet goed zien waarom het niet past.
Own experience is important to build confidence in the measurements	En aan de andere kant is het uiteindelijk ook zeg maar het gevoel dat je in de praktijk krijgt van dit klopt bij hoe ik de patiënt ervaar of wat de patiënt vertelt. En wat ik net vertelde over die PKG dat, dan is de wetenschappelijke onderbouwing is best stevig, maar toch op het moment dat ik er dan zelf gebruik van ging maken denk ik van mwoah.
Access to data during consultations	Maar het is voor mij vooral relevant als de patiënt voor mij zit.
Access to data for patients	De patiënt zou alleen toegang tot de samenvatting kunnen krijgen

Parkinson nurses: patient education & self-management

Code	Illustrative quote
**Goals**	
Focus on what patients find important	Als je vraagt: vindt u dat acceptabel of niet en als het antwoord nee is, dan moet ik er iets mee en als het antwoord ja is, dan hoef ik er niks mee. Dan kun je er van alles van denken, maar de cliënt blijft het uitgangspunt, want daar komen we eigenlijk steeds op terug.
Stimulate patient self-management	Interviewer: Zijn jullie het ermee eens dat het self-management een belangrijk onderwerp is?<br>Nurse: Absoluut.
**Gains**	
Home visits provide useful information	Mensen thuis treffen is toch wezenlijk anders vind ik dan dat ze gewassen en gestreken op het spreekuur komen. Daar heb ik ook heel veel van geleerd om ook thuis samen te werken met de patiënt.
**Pains**	
It is difficult for patients to describe the daily patterns of symptoms	mensen vinden het heel moeilijk om aan ons uit te leggen: ik ervaar dit als ik om 3 uur mijn medicatie moet nemen en ik ervaar tussen 2 en 3 dit en als wij dan heel concreet gaan vragen: 'wat ervaart u dan en hoe uit zich dat dan', vinden mensen dat vaak heel lastig om te benoemen.
Some patients underestimate the duration of sleep	Soms of soms is het de beleving van een patiënt dat hij 3 uurtjes slaapt en na 4 uur 's ochtends niet meer en dan lijkt dat toch anders te zijn. en ik vind dat best wel een lastig punt altijd om dat te bespreken.
On off diaries are unreliable	Ik denk ook bij het invullen vraag ik me af: hoe reëel is het invullen, want door de stress die het geeft om het in te vullen, veranderen klachten natuurlijk ook zoals ze eigenlijk normaal zijn.
Filling out diaries is burdensome for patients	maar wij hebben een beetje de strategie aangewend om te vragen of ze bijvoorbeeld een paar dagen voordat ze weer op de polikliniek komen of voordat we een telefonisch consult hebben het te doen zodat ze het niet elke dag doen. Want elke dag gemeten worden is voor veel mensen toch best zwaar. Er loopt continu of een

	controleur met je mee of een echtgenote die zegt: 'je moet het nog invullen', en dan worden mensen wel eens een beetje tureluurs van
Filling out diaries can become an obsession for some patients	en de mensen die door hun Parkinson zeer rigide worden in hun gedrag en elke keer weer alles willen rationaliseren in de cijfers, die komen wel eens met hele pakketten van informatie en dat de partners ook zeggen: 'we kunnen de deur nog niet uit gaan of de pen en papier moet mee of dat ze hele schema's bij zich hebben en grafieken. Dat is echt obsessief gedrag. Dat is een deel van de mensen, maar je hebt natuurlijk heel veel verschillende.
Some patients find it difficult to accept the diagnosis	Parkinson is een ziekte, die komen binnen bij de neuroloog en die zegt: 'u hebt Parkinson'. Dan vinden mensen al: dat kan niet, niks gedaan, maar de neurologen zien dat, maar daarom blijft het heel vaak voor mensen wel een ziekte van: klopt de diagnose wel, want ik kan geen foto maken.
It is difficult to evaluate patients with cognitive problems and without partner	Mensen met cognitieve problemen of mensen met dementie, die waren voor mij in het verpleeghuis en in de thuiszorg heel moeilijk soms om te beoordelen, want het is niet betrouwbaar en als je dan geen partner hebt of je hebt een zorgafdeling die niet goed geschoold is in Parkinson, dan zit ik dus met een hele moeilijke casus en ik ben heel benieuwd of dat daar dan ook een meerwaarde in zou kunnen zijn.
**Expected advantages of wearables sensors**	
Increase self-awareness of patients	Ik zou tientallen mensen kunnen noemen waarvan ik denk: ja dat is eigenlijk wel mooi om inzichtelijk te maken: wat is er nou gebeurd en daar nog eens op terugkomen.<br><br>Wel dat ze weten, tremor of overbeweeglijk is voor sommige mensen nog wel eens lastig. Als jij overbeweeglijk bent, dan ga ik nog eens extra dopamine nemen, dan wordt het alleen maar erger. Dus dan kom je wel eens dat mensen daardoor beter ziekeninzicht krijgen en weten: dit is helemaal niet overbeweeglijk, ik heb tremor of ik ben juist overbeweeglijk, ik moet helemaal niks extra pakken, ik moet juist minderen.<br><br>Daar had ik het met jou vanmiddag nog over, ik had een consult en als die in een off raakt kan dat ze heel erg in paniek raken, maar ze herkent het off fenomeen nog niet helemaal, waardoor waarschijnlijk ze gaat hyperventileren met alle gevolgen van dien. Dan zou het haar juist misschien kunnen helpen als zij tegen mij kan zeggen: 'vorige week woensdag om 12 uur had ik zo'n aanval', en dat we dan kunnen bespreken: het lijkt echt op off en dat het dan ook bevestigt in de grafiek.<br><br>Ze zeggen wel eens tegen ons: 'ik beweeg zoveel', en dan komt er uit dat ding dat ze heel veel, dan zie je echt dat ze heel veel stil zitten.
Disentangle what is related to PD and what is not	Mensen hebben ook vaak op een gegeven moment andere klachten die niet altijd Parkinson gerelateerd zijn, maar dan wel vaak gedacht wordt: zou dat bij Parkinson horen. Je maakt meten is weten inzichtelijk: dit gebeurt er en dit merkt u. Dan kan het ook zijn dat dat iets anders is, de klacht die u nu heeft.

Supports the patient to tell his story	Ze moeten het al doen met minder dopamine, dus het voeren van een gesprek kost veel energie. Ik kan me voorstellen dat het een voordeel heeft als je al wat getallen hebt zodat de patiënt dat niet helemaal aan jou hoeft over te brengen, dat het al ergens staat
Provides something concrete to discuss with patients	Ja, we hebben heel lang niks gehad om te meten. Je hebt geen inzichtelijk iets. Je hebt misschien je vinger gebroken, we maken een foto en we zien die breuk en hier zit het, maar Parkinson is een ziekte die je niet kon meten en nu heb je iets, je laat die grafiek aan de mensen zien, je bespreekt het. Kijk, je ziet dat je overbeweeglijk bent. Dus je bespreekt eigenlijk samen wat je ziet.
More time for conversation with the patient	Ja het scheelt misschien in die zin tijd dat je daardoor juist meer tijd kan besteden aan het gesprek.
Give targeted advice on how patients can prevent falls themselves	Ja wat de reden is, dan kun je tenminste de oorzaak herleiden en dan is de oplossing ook makkelijker. Dan zou je zeggen: ik had rustiger op moeten staan en dan vervolgens kan vertellen dat het daar en daar en daardoor kwam, dan weet je dat, maar als iemand een zwart gat heeft en het niet meer weet, dan kunnen ze zichzelf ook niet helpen.
Increase treatment compliance	Absoluut en misschien daardoor ook motivatie om je pillen echt op tijd in te nemen en dat soort gevolgen. Die verbetering van inzicht heeft vaak positieve gevolgen.
Support acceptation of the diagnosis	daarom blijft het heel vaak voor mensen wel een ziekte van: klopt de diagnose wel, want ik kan geen foto maken. Ik kan een MRI maken, sluit ik uit dat het niks anders is. Dit is voor mensen vaak wel echt iets wat ze zien, iets wat gemeten wordt. Meten is weten.
Stimulate inactive patients to become more active	Dat doet me denken aan een ander idee, als een sensor bepaalde prikkels geeft voor initiatiefverlies en houding, dat zou een mantelzorger wel wat kunnen ontlasten omdat hij niet continu de partner hoeft te prikkelen en in die verzorgende rol zit en veel meer die partner kan blijven.
Coach to maintain healthy walking pattern	Ik heb wel eens gedacht aan een soort muziekcue die rustig is op het moment dat iemand gaat staan. Ik ken twee patiënten die hebben echt daar last van, dat ze lopen te rollen achter de rollator en zo. Dan denk ik wel eens: als diegenen bij mij gezeten hebben, dan zeg ik: ‘probeer eens heel rustig erbij te staan, een beetje rustig praten en eerste stap, tweede stap. Ik denk: eigenlijk zou er ergens iets moeten zijn bij hem, het zijn twee mannen die ik ken, die even manend tot rust bij het weer omschakelen.
Early signaling of problems that need medical attention	Als je een signaaltje krijgt, net als de personenalarmering, dan bel je op of je gaat langs en dan kan je ter plekke bekijken wat er aan de hand is. Als je dan constateert: wellicht is het een blaasontsteking, dan kan je daarop voordat de patiënt dat zelf in de gaten heeft, ben je misschien al 2 dagen verder als er geen mantelzorg is.
Give the patient a feeling of safety	Ik denk dat dat wel denkbaar is, dat mensen dat soms heel ondersteunend vinden van ik word ik de gaten gehouden. Je hebt ook personenalarmering, dan merk je ook, ze gebruiken het niet. Als ze vallen drukken ze niet eens op de knop, maar gewoon het idee dat die knop er is om te kunnen drukken geeft een veilig idee.
It is easier for patients	Ik denk dat het voor mensen een stuk gemakkelijker is als de hele dag pen en papier.

Obtain more insights into what happens at night	Slapen, in hoeverre diep slapen en wanneer ik wakker ben, mensen zeggen: 'ik slaap slecht', maar ze kunnen niet benoemen wat dat slecht slapen dan is en dat zou dan ook mooi zijn als je dat, hoe kan dat.
Obtain insight into fall incidents	Ik vind vallen bijvoorbeeld wel zo'n ding. Als je kunt registreren, als je al denkt aan valgevaar en iemand woont alleen of je hoort terug van: nee ik ben niet meer gevallen, maar je hebt echt je twijfel, dan zou het natuurlijk wel ergens misschien, als er een schok komt, iets kunnen registreren.
Support setting up duodopa pump treatment	ook wel als mensen bijvoorbeeld een Duodopa pomp krijgen, dat je van tevoren en daarna meet.
**Expected barriers / disadvantages of wearables sensors**	
Subjective experiences are more important	Ik zou vooral willen weten hoe diegene zelf de nacht ervaart. Ik vind dat plassen, dat is goed om te weten, maar als diegene 4 keer per nacht moet plassen, maar daarna altijd vlot weer in slaap valt, dat is wat ik wil weten, want stel dat ik op die 4 keren af zou gaan en dan zou interpreteren: dat is een onrustige nacht, dat vind ik wel tricky.
Too much focus on numbers	haar verhaal doet ertoe voor mij en ik denk zodra je meet en je hebt de harde getallen, ik ben een beetje op mijn hoede dat we niet te veel op die harde getallen afgaan.
No replacement for face-to-face contact	Ik denk ook niet dat dat de bedoeling moet zijn van een sensor, dat het je gesprek of in ieder geval face to face, dat dat het niet vervangt.
Difficult to measure non-motor symptoms, which are important for patients	Waar ik heel benieuwd naar ben, überhaupt met alles wat wij registreren is de non-motorische offs. Wij maken steeds meer mee, we hebben dat eigenlijk ook geleerd van onze jonge mensenpoli, dat er wordt gezegd: die motoriek staat voorop. Dat meten we en dat weten we en dat is fijn, maar juist die voor veel mensen belangrijkere dingen van: hoe gaat het met mij als ik me off voel, dat kun je natuurlijk niet registreren.
A coaching device may scare some patients	Dan valt iemand denk ik omver omdat die schrikt.
It may be too difficult for patients	Ja, bij de meesten gaat het goed, maar af en toe is er iemand die wordt er helemaal tureluurs van en die doet het iedere keer af.
More information may raise more questions	Wat we nu al zien dat als mensen dingen in kunnen lezen, dan gaan ze natuurlijk bellen, want ze willen uitleg. Het is moeilijk te lezen voor een patiënt of je moet net een buurman hebben die huisarts is, dan helpt het wel weer, maar de mensen voelen zich er soms ook onzeker door, omdat ze al dingen in kunnen zien die ze nog niet hebben besproken. Dan gaan ze toch vaak mailen of bellen en dat kost erg veel tijd. Ik vind het wel goed dat het gebeurt.
Time investment and costs	Ik zit me wel af te vragen. Je hebt het verslag van de sensor, of het dan niet net zoveel tijd kost dan als ik alles moet uitvragen in je consult.
**Ideal tool**	
Targeted use for specific patients	Je doet het ook niet bij iedereen, tenminste zo zetten wij het nu nog niet in. We doen het echt op indicatie, zoals je voorheen die kaart meegaf. Hier kom ik niet uit, wat gebeurt er allemaal. Mensen kunnen dat moeilijk vertellen.
Access to data during consultations	Ik heb niet de meerwaarde van, want ik kom op huisbezoek en tenzij er iets is, dan ben ik niet meer met de cliënt bezig, maar dan ben ik

	met een andere cliënt bezig. Dan hoef ik ook geen gegevens te krijgen, tenzij er een bijzonderheid is, maar van bijzonderheden weet ik genoeg wanneer ik het weer met de cliënt ga bespreken. Voor mij zou het geen meerwaarde hebben om het real time live binnen te krijgen.
Clear agreements on use of sensors	Ik denk dat wij niks te vinden hebben, als die patiënt geen toestemming geeft, geeft hij geen toestemming.
Combination of objective and subjective information	Als er dan onduidelijke registratie is, zeg dat zo'n smiley toegevoegd wordt en cliënten geven dan elke keer stemming mineur aan, terwijl dat het niet klopt met de rest van de gegevens, dan lijkt het me juist heel erg cool om dat wel te kunnen zien, want je hebt het niet goed geïnterpreteerd of er is iets waardoor dat verkeerd opgenomen wordt, dat de cliënt het verkeerd aanvinkt.
Focus on changes within person	Ik vraag me eerst af: wat is je uitgangswaarde. Neem je mijn uitgangswaarde, hoe ik normaal functioneer of hoe ga je dat doen? Jij beweegt weer heel anders dan dat ik beweeg.
Validation is important	Als het een gedegen onderzoek is, lijkt mij fijn. Als je bijvoorbeeld een distonie hebt van je rechtervoet en je hebt de sok aan die dat echt kan meten hoe distoon je bent, ik noem maar wat, dat het bewezen is dat het echt te registreren is hoe dat zit met de distonie. Dat lijkt me wel fijn als er een meting over is.
Trust in measurement when the patient recognizes it	Ik zou het bij de meeste vertrouwen als je het bekijkt samen met de patiënt en dat die meerdere malen zegt: 'ja dat klopt, dat ervaar ik ook zo'. En inderdaad je zou er eigenlijk zo'n boekje naast moeten leggen.
Personalized coaching	En op maat. De een heeft een piep nodig en de ander heeft een muziekje nodig, dat je in kunt stellen.
Balance between too little and too much stimulation	Die partner is ook de hele tijd aan het prikkelen. Het moet niet te veel zijn, want dan gaat het zo erg irriteren dat ze de sensor het raam uit doen vliegen. Dat kunnen ze met partner iets moeilijker, maar daar moet wel een balans in zitten. Daar zit dan wel een uitdaging in.
Include a rewarding system	Een beloningssysteem denk ik ook. Het is ook wel leuk dat je iets goed doet.
Signaling function in first place directed to caregiver	E-mail alert als er een bijzonderheid is, dat het systeem een bijzonderheid registreert, dat er dan een alert sms-je, een whatsappje. Ik zou zeggen naar de mantelzorg.

PD patients: share information with healthcare providers

**Code**	**Illustrative quote**
**Goals**	
Communicate how I am doing with care providers	Jij bent degene die vertellen kan hoe het gaat, niemand kan het verder zien. Je moet een manier vinden om het over te brengen.
Deal with Parkinson's day by day	Het enige wat ik kan doen is vandaag zorgen dat ik lekker kan draaien en ik zie wel wat er op me afkomt.
Stay active	en zorgen vind ik dat je altijd actief kan blijven. Als ik het ene niet meer kan, ik kan niet meer 16 km. wandelen door het bos, dan ga ik iets anders zoeken wat ik nog wel kan en dan kun je plezier blijven houden. Dan zie ik wel hoe snel het gaat.

Find the right medication schedule	Een operatie is dat, het is een sonde door je maagwand heen, die gaat naar je dunne darm en dat is verbonden met dat pompje en dat geeft de hele dag door pulsen in een bepaalde hoeveelheid. Die hoeveelheid, dat is bij iedereen een beetje anders. Ze zoeken in het begin: wat is de goede hoeveelheid voor deze patiënt.
Understand influence of diet	Misschien dat je daar in de loop der tijd vanzelf achter komt als je iets eet en je schrijft het op. Wat heb ik gegeten de andere dag of 's avonds en dat je dan de dag daarop een mindere dag hebt, dat zou kunnen. Als je dat allemaal opschrijft, dan weet je misschien bepaalde dingen dat je eigenlijk niet moet nemen.
Measure progression over longer time periods	Je moet achteruitgang kunnen meten of vooruitgang, maar in ieder geval aan onze kant achteruit en nou ben ik heilig gelovig in getallen en in trends en zo. Ik ben wiskundige van huis uit, dus toen ik van deze applicatie hoorde, toen dacht ik: daar wil ik aan meedoen graag.
**Gains**	
Making notes to prepare for my neurologist's appointment	Nu ook voordat ik naar de neuroloog ga, ik maak altijd een A4tje met wat ik te zeggen heb, zodat ik niks vergeet. Dan kijk ik ook naar wat ik 3 maanden geleden gedaan heb en weet je het weer gelijk omdat ik het opgeschreven heb.
My partner/friends help to tell my care providers how I'm doing	Ik heb het idee dat mijn vrouw een betere graadmeter is dan mijn neuroloog. Zeker in het begin moest ik heel erg: hoeveel medicijnen moet ik pakken en dan zei mijn vrouw al een keer: 'volgens mij gaat het minder met jou'. Die zag natuurlijk mijn gezicht en weet ik het allemaal
Thanks to the Parkinson well-being map, my neurologist already knows what is going on	Als ik dan kom, dan weten ze hier al wat er aan de hand is, of ik me beter voel, dat lukt bijna nooit.
My care providers listen to my story	Die neuroloog doet hetzelfde als wat [naam deelnemer] net zei, die kijkt naar hoe je opstaat en hoe je loopt, alleen die trekt niet de conclusie, maar hij vraagt aan mij: 'hoe gaat het ermee?' Meestal zeg ik: 'het gaat prima, het gaat goed'. Zo voel ik dat ook.
My physical therapist walks together with me to know how I am doing	Mijn fysiotherapeut gaat ook iedere keer een blokje met me rondlopen en dan volgt hij hoe het gaat. Dan heb je toch wat om te meten.
I tell my care providers what I would like to discuss	Toen heb ik hem dus een artikel toegestuurd en de volgende keer zei hij: 'heel goed dat jij dat gegeven hebt, ik kan dat heel goed aanvullen'. Ik zet hem gewoon aan het werk, ze zijn er voor jou. Ik stuur hem gewoon dingen toe: dit zou ik graag willen bespreken de volgende keer.
My neurologist is happy with the information I collect	Mijn neuroloog zegt dan: 'ik ben blij dat u wel een formulier heeft, want ik ben van jou', ze is van mij afhankelijk. Ze kan alleen helpen maar als ik wat zeg, anders kan ze me niet helpen, dus als ik niks weet, als jij binnenkomt en je zegt: ik weet niet wat. Nou kan ik je veel beter helpen nou. Ik geef het door met dat papier. Dat is wel het voordeel.
I have a pleasant relationship with my Parkinson nurse	Het prettigste gevoel krijgen we eigenlijk bij de Parkinson verpleegkundige en dat is raar, want op zich, die weet natuurlijk niet alles. Die heeft misschien niet 100% inzage in het dossier, maar het is wel iemand die oog heeft voor de sociale kanten en die denkt met

	je mee als je zegt: 'ik had een vakantie en die was zo leuk'. Het is meer dichterbij, die is bijna net zo dichtbij als een fysiotherapeut lijkt het wel.
The guidance of my physiotherapist is very helpful	Die fysiotherapeut, daar kom ik elke week en daar bespreek ik ook als ik wat bespreek altijd dan met haar en zij weet ook veel, want zij heeft allemaal patiënten zoals ik, Parkinson mensen, dus daar heb ik eigenlijk het meest aan.
My care providers communicate with each other	ParkinsonNet zorgverleners weten dat gewoon van elkaar. Als ik bij de neuroloog kom zegt ze: 'nou het ging slecht hè'. Het ging zeker slecht. Als ik bij de fysiotherapeut komt zegt hij: 'je bent van de week daar geweest en ze vond dat je de volgende medicijnen moest hebben'. Dan weet hij dat ook. Als het dan beter gaat, dan weet hij ook waardoor het beter gaat, niet dat hij het gedaan heeft, maar dat de medicijnen het doen bij wijze van spreken. Dat werkt feilloos.
**Pains**	
The Parkinson well-being map is subjective	Ik heb hem geprobeerd en ik vond het veel te subjectief. Je moet een cijfer geven en ik weet niet, als ik u een 6 geef en de vorige keer ook een 6 gegeven, weet ik niet waarom ik toen een 6 gegeven heb.
Filling out diaries takes a lot of time	Dat moest ik ook bijhouden en dat bijhouden viel dan wel mee, alleen het was zoveel, je kwam gewoon tijd tekort om het allemaal op te schrijven.
Keeping track of falls is difficult	Bij mij, iedere keer als je ging lopen, dan moest je de tijd opgeven. Dan was ik de hond aan het uitlaten, kwam ik een uur naderhand terug. Ik ben dan 10 keer gevallen, maar gegokt, gewoon iedere keer geteld en de tijd heb ik er niet bij kunnen zetten.
I sometimes forget things when I talk to my neurologist	Ja ik ga om de 3 maanden naar de neuroloog. Inderdaad ik zou het beter kunnen noteren, want dan denk ik: ik moet nog wat vragen, wat is het dan in godsnaam.
My neurologist only sees how I am doing during the appointment	De neuroloog kijkt als ik binnenkom hoe ik loop en mijn gelaatsuitdrukking en die zegt dan: 'het gaat goed met jou [naam deelnemer]'. Zo bepaalt hij dat.
My neurologist does not see the value of the Parkinson well-being map	Ik ken hem wel van een voorlichting, maar de neuroloog vindt het onzin en die werkt er niet aan mee, dus dan houdt het op.
My neurologist has little time	Bij de neuroloog is het vaak: 'ik heb over 10 minuten weer de volgende', dus vlug, vlug, vlug.
My general practitioner knows little about PD	Ik heb gemerkt dat de huisartsen heel weinig weten van Parkinson.
More information about PD is confronting	misschien ben ik een rare, misschien herkennen mensen dat. Ik ben meteen lid geworden van de Parkinsonvereniging en van het Parkinson café, want ik ben heel leergierig, dus ik dacht: ik wil alles over die ziekte weten en ik werd er van dag tot dag heel treurig over. Ik sliep er niet meer van, ik werd er depressief van, ik denk: jonge jonge, wat staat me allemaal te wachten en toen heb ik dat met de neuroloog besproken en die zei: 'wegdoen, daar ben jij gewoon niet geschikt voor om al die informatie tot je te nemen'. Dus kop in het zand gestoken, struisvogelpolitiek.
I do not know which PD app is good	Maar ik begrijp dat een Parkinson app, dat kan je dat soort dingen ook makkelijk bijhouden. Als ik bij de app store kijk en je kijkt naar Parkinson, er zijn zoveel applicaties. Ik weet niet wat goed is.

Finding the right medication schedule takes a long time	Ja. Instellen duurt het bij mij heel lang. Op gegeven moment ga je de overweging maken: pak ik andere medicijnen of blijf ik toch nog even volhouden. Uiteindelijk heb ik dan gekozen om dezelfde medicijnen vol te houden. Toen op een gegeven moment sloeg het wel aan. Dat heeft bij mij echt maanden geduurd.
There is no pattern in my PD symptoms	Ik ben ermee begonnen, alleen bij mij is elke dag anders. Er zit helemaal geen logica in, dus op een gegeven moment heb ik zoiets: wat heeft het voor zin om het bij te houden. Als ik een nacht goed slaap kan ik bijvoorbeeld de dag heel slecht doormaken. Andersom kan ook, er zit helemaal geen logica in. Dat is lastig en daarom heeft bijhouden voor mij geen zin.
It is difficult to anticipate	Nu gebeurt er iets en je denkt: voor je het kan plaatsen is het al een paar maanden verder en dan kun je terugkijken. Het maakt het moeilijk om vooruit, want je wil vooruit kijken, om het volgende jaar goed in te richten.
It is difficult to distinguish between PD and general complaints	Op een gegeven moment krijg je bepaalde klachten en dan denk ik: het zal wel aan Parkinson liggen. Die dingen schrijf ik dan ook op en dan denk ik: ik heb dit meegemaakt, ligt dat aan Parkinson of is dat gewoon wat iedereen kan overkomen.
It took a long time before the diagnosis became clear	Ja, ontdekken of iemand Parkinson heeft, dat is kennelijk moeilijk, want mijn ervaring is wel van vele jaren geleden, ik had problemen met schrijven, een soort schrijfkramp en dar arbo-arts zei: 'nee Parkinson is het niet', want ik had geen tremor. Ik ben naar de neuroloog geweest, die heeft nogal wat testjes gedaan. Distonie zou het kunnen zijn, maar een collega van hem was daar beter in en toen kwam ik eindelijk bij de neuroloog waar ik dus al jaren loop en die zei: 'hebt u schrijfkramp, schrijf eens even wat', en dan krijg je die karakteristiek.
**Expected advantages of wearables sensors**	
Support care providers to find optimal therapy	Wellicht geeft het aanwijzingen voor het instellen van therapie op een gegeven moment. Ik zou meer dit doen of meer dat doen en of dat nou medicijnen is of bewegingsactiviteiten of wat dan ook, als het maar iets oplevert.
Adjust medication schedule more efficiently	Stel je voor dat je bij een bezoek afspreekt dat de medicatie verhoogd moet worden omdat je te passief bent. Dan kun je de komende twee weken kijken: is het veranderd of niet. Nou niet, nou wacht je 3 maanden tot je bij hem terugkomt en je zegt: 'ja het heeft wel geholpen maar..'
Support setting up duodopa pump treatment	want als je zoiets krijgt, moet je een week naar het ziekenhuis om te kijken: hoe is die instelling. Dat helpt dan wel, want als het goed ingesteld is, dan krijg je het definitieve medicijn door je neus neem ik aan, een slangetje en als het goed werkt, dan gaat het door de maag. Ze hebben het degelijk goed ingesteld en dan zijn er geen problemen meer. Dan moet dat met zo'n ding een stuk beter gaan.
Targeted advice on which exercise I can do	Met wandelen, soms gaat het soepel en soms denk ik: flap, flap, flap. Dan denk ik: wat voor oefeningen moet ik daar nou voor doen, dus dan denk ik: we moeten eigenlijk, nou heb ik een analyse van de bewegingen met sensoren en dan een advies krijgen welke oefeningen je nou moet doen.
Less frequent hospital visits	Ik denk dat het voordeel kan zijn als je het wel deelt dat je minder naar het ziekenhuis toe hoeft zelfs, dat je het rechtstreek vanuit

	thuis kunt regelen. Dat zou het mooiste zijn, dat je minder naar het ziekenhuis moet, dat je het rechtstreeks kan, dat zou mooi zijn. Sommige ziekenhuizen werken al zo met andere dingen, ook als ze erbij zijn.
Care providers are more prepared for appointments	Ik denk dat ze dan nog meer voorbereid zijn op hetgeen wat er al gebeurd is, dat ze echt van tevoren in kunnen lezen. Dan hoef je niet echt alles naar boven te halen, omdat zij allemaal op de hoogte zijn.
Share measurements with my physical therapist	Precies, maar ik zou het graag willen dat mijn fysiotherapeut, dat heb ik net verteld dat we een rondje lopen. Dat zou ook in zo'n app kunnen, dat je dan gewoon de tijd bijhoudt hoe lang het gaat, hoe je stappen zijn en dat je ook gewoon weet hoe lang je erover doet en of je de stappen groot maakt en zo. Dat scheelt dus weer een fysiotherapeut, dat kan gewoon naar hem toegestuurd worden.
Early signaling of problems that need medical attention	In eerste instantie zou ik denken bij mezelf: ik neem het mee naar de neuroloog, maar aan de andere kant kan je het misschien ook eerder delen en als de neuroloog er dan wat mee doet, tenminste niet wacht tot jij op bezoek komt. Als hij er niets mee doet, dan zou ik het zeker niet sturen, maar dat kunnen ze misschien al eerder zien aan de gegevens van: daar gaat iets niet goed, misschien moeten we al eerder weer terug op controle komen.
Obtain an overview of the week	Ik heb wel eens schema's gezien van mensen die altijd pijn hebben, geen Parkinson, maar pijn en dan zie je iets met kleurtjes. Die moeten dan in kleurtjes aangeven, want je hebt zo ontzettend veel informatie, dat moet je een beetje kunnen overzien en als je nou een week hebt met kleurtjes, dan kan je wat van tonen in herkennen en misschien voor jezelf analyseren van: het ging goed van de week, het ging wat minder goed, dus met kleurtjes ergens, bijvoorbeeld in je app aangeven, dat je om de 2 uur of zo een piepertje krijgt en dat je even een 1, een 2 of een 3 moet invullen. Dan krijg je meer inzicht en dat kan je ook aan de neuroloog laten zien.
Disentangle what is related to PD and what is not	Dan ga je met een schonere agenda naar je specialist dan wanneer alles maar op dat bordje is gekomen van alles wat je hebt meegemaakt. Dat wordt een enorme brei en vieze spaghetti waar je eigenlijk niet meer goed de weg in weet. Op het moment dat je daar wat in kun elimineren, dan hou je in feite de echte dingen over die over Parkinson gaan.
Obtain more insights into what happens at night	Ik zou bijvoorbeeld best willen weten wat er gebeurt als ik 's nachts ga slapen. Draai ik me om of is het inderdaad wat ik denk, ik heb soms dat ik urenlang op een kant lig, dat je bij wijze van spreken een glas water op mijn lijf kan zetten en dat het er de volgende morgen nog staat. Ik kan me niet heugen dat ik me omdraai of wat dan ook en als het gebeurt gaat het heel moeizaam. Als dat soort informatie bijvoorbeeld bij de neuroloog zou komen, dat hij zou denken: daar moeten we misschien wat aan doen en dat zou op zich wel prettig zijn.
I would trust the sensor measurements more than my own subjective impression	Ik zit voor mezelf nog te bedenken, ik zou dan voor nu een sensor op mijn arm eigenlijk wel nuttig vinden, want voor mijn gevoel werkt het niet, maar misschien als je sec naar de cijfertjes gaat kijken, doet het misschien wel iets. Dat je gevoel werkelijkheid of niet, dat weten we dan.

Predict when I will fall	dat zou fijn zijn, dat ik kan voorspellen wanneer ik ga verliezen. Dan val ik niet meer en nou val ik iedere keer omdat het onverwacht is. Ik mag gegarandeerd niet draaien, ik moet echt helemaal rondlopen, als ik ga draaien, dan val ik meteen om. Heel raar is dat.
Predict when I will have freezing of gait	Ik zal het misschien een seconde eerder merken als dat mijn hersens werken. Die sensor moet sneller zijn dan mijn hersens. Dan kan die mij een signaal geven.
Coach to maintain healthy walking pattern	Mijn fysiotherapeut zegt: 'rechtop lopen, grote passen maken, breed lopen en zo'. Volgens mij een sensor die dat in de gaten houdt en mij herinnert: 'rechtop'. Als ik aan het dansen ben, dan loop ik rechtop, want anders kan ik niet draaien. Iedere keer als ik draai ga ik rechtop. Als nou die sensor mij zegt: '[naam deelnemer], je zakt weer in mekaar, hup', dan helpt dat mij om fysiek beter te blijven.
Optimize when I eat and when I take my medication	Ik neem mijn medicijnen in en ik eet niet gelijk daarna, dat doe ik daarvoor. Dan wil ik dat opschrijven en die sensor meet wat het met mijn lichaam doet. Dat zou ik wel graag willen weten of ik daar iets aan kan verbeteren of iets wijzer kan worden.
Predict how the disease will develop	Als er een soort patroon in zou zijn, als zo'n sensor dat zou kunnen meten en kan indiceren, dan weet je wat er gebeurt. Nu gebeurt er iets en je denkt: voor je het kan plaatsen is het al een paar maanden verder en dan kun je terugkijken. Het maakt het moeilijk om vooruit, want je wil vooruit kijken, om het volgende jaar goed in te richten.
Obtain insights into progression over long time periods	Ik heb zelf wel runkeeper gebruikt om te kijken: hoeveel kilometer loop ik, wat is de gemiddelde snelheid, zit er verval in. Van de zomer kon ik nog net zoveel lopen als twee jaar geleden, dat ging net zo goed. Dat gaat nou niet meer. Dan moet je er toch iets mee kunnen, met dat soort gegevens.
Focus on different things than tracking	Ik denk juist dat zo'n sensor de focus op andere dingen kan, want die bewegingenregistratie, dat loopt dan wel. Daar zorgt de hardware voor. Dan kun je andere dingen doen.
Support acceptation of the diagnosis	Op een gegeven moment had dat mij denk ik ook een stuk rust gegeven en misschien ook het accepteren ervan, dat dat ook versneld was. Dat er eerder een belletje was gaan rinkelen van: je gaat gewoon te ver. Ik bleef die 40 uur of 50 uur werken, terwijl het eigenlijk helemaal niet meer kon. Ook die medicijnen dat ging niet goed.
**Expected barriers / disadvantages of wearables sensors**	
It can become an obsession for patients	Een nadeel zou kunnen zijn dat je je leven helemaal laat leiden door de sensoren en zo. Daar ben ik nou ook niet helemaal kapot van.
No replacement for face-to-face contact	Ik meen dat er niks boven persoonlijk contact gaat.
Proactive signaling may have little added value: patients need to have a request for help	Er zijn mensen die hebben een tremor en die zeggen: 'dat hoort erbij'. En als een neuroloog dan ziet: die heeft een tremor en die tremor neemt toe, moet hij dan gaan bellen, moet hij niet gaan bellen. Misschien vindt hij het helemaal niet erg dat die tremor toeneemt. Je kunt ook zelf een keuze maken en denken: het gaat nu wat minder, ik ga er eerder naartoe. Dan ligt het meer bij jezelf en dat vind ik eerlijk gezegd prettiger dan dat de neuroloog contact opneemt.
Time investment and costs	Ik heb het idee dat het tenminste mijn ervaring is, als ik bij de neuroloog kom, dat hij op dat moment eigenlijk pas goed gaat

	luisteren en kijken wat er aan de hand is, maar in ieder geval niet van hem uit zo'n actief beleid om mij te monitoren.
The collected information does not change anything	ik kan het wel op gaan schrijven, maar ik word er echt niet beter van.
Limited reliability of measurements	Een sensor geeft alleen objectieve gegevens als die ook echt goed is. Dat systeem van jaren geleden kon bepaalde bewegingen, fietsen dacht ik, bijvoorbeeld niet detecteren. Dan houdt het onmiddellijk op, dan is het niet een compleet objectief beeld.
Patient privacy	Als ik mag zeggen wie en wanneer, dan vind ik het goed. Mijn fysiotherapeut mag het zien. Een andere misschien niet, maar mijn fysiotherapeut mag het zeker zien, want die zie ik iedere 14 dagen.
**Ideal tool**	
Data should always be accessible to care providers	Stel je voor dat er iets zou gebeuren en ik heb een vraag, dan kan hij kijken: hoe zit het daarmee. Het is beschikbaar. Het kan geen kwaad dat het beschikbaar is voor degene die je vertrouwt.
I would like to personally hand over the data to my care providers	Patient: Dat ik met mijn gegevens naar de neuroloog of naar de Parkinsons verpleegkundige of..<br>Interviewer: Dat u de informatie zelf heeft.<br>Patient: Ja het gevoel wat ik erbij heb.
Care providers may proactively reach out to me	Interviewer: Wat zou u ervan vinden als metingen worden gedeeld met de neuroloog automatisch, dus de hele tijd en hij ziet dingen waarvan hij denkt: hé hier zou ik misschien bij kunnen helpen. Misschien moeten we iets aanpassen met de medicijnen en hij neemt contact op met u, zonder dat u daar zelf om gevraagd heeft. Hoe zou u dat vinden?<br>Patient: Ik wil beter leven. Ik zou het een plus vinden.
Access to data for patients	Ik vind als je er behoefte aan hebt dat je erbij moet kunnen.
I want access to a clear summary	De informatie van die sensoren zou gevat moeten worden in een soort cijfer of zo, maar niet in een tijdregistratie. Daar is allemaal geen tijd voor, je moet dat proberen te comprimeren en zeggen: vorige keer was het 8,6 en nu is het 8,4. Dan kun je het daarover hebben. Als het nog 8,6 is hoef je niet al die informatie door te spitten. Dat is datareductie.
Targeted use for specific patients	Ja, gegevens zijn heel mooi, maar binnen de kortste keren verdrink je in alle gegevens, dus je moet er een soort lijn of je moet een doel hebben. Ik heb een doel: bezig blijven.
Coordination by Parkinson nurse	Het gaat juist om de coördinatie en dat vind ik heel goed voor Parkinson verpleegkundigen.
Able to measure for 24 hours per day	Ik hoorde net een nadeel dat men zo'n sensor afdoet. Ik denk dat je juist een sensor 24 uur zou moeten dragen om een compleet beeld te krijgen.
Does not interrupt sleep	Ik slaap heel slecht, maar ik slaap ook heel onrustig, dus ik weet niet, ik heb geen idee hoor als je dat ding om je pols draagt, of dat je iets kunt verstoren.
Accurate information about what the sensor can measure is important	Tenzij je weet welke bewegingen wel en welke bewegingen niet. Ik wist nu niet van deze test: welke bewegingen worden wel opgeschreven, wanneer ik ging fietsen, wanneer ik ging lopen. Kennelijk alleen maar gewandeld.
Preference for passive over active registrations	Ik denk dat je er niet extra voor moet gaan bewegen. Hij moet vanzelf al gaan meten vind ik, dat ik niet moet zeggen: 'nu meet je me wel en nu meet je me niet'. Of zeg ik nou iets geks?

Smart timing of active tasks	Patient 1: Een slimme sensor weet dat je in de auto zit aan het bewegen, dus als ze slim zijn zou hij geen piep geven.<br>Interviewer: Dus we moeten sowieso goede momenten hebben.<br>Patient 2: De momenten uitkiezen.<br>Interviewer: En als het wel op rustige momenten zou kunnen, als u thuis zou zijn en u zou af en toe wat moeten doen?<br>Patient 2: Dat zou ik niet zo erg vinden. Maar ja, het komt niet altijd uit, hè Piet als je aan het bridgen bent en je moet dan iets uit gaan voeren, dan wordt het wel lastig.<br>Patient 1: Dan moet je een schema hebben waar je zelf in kan stellen wanneer dat is.<br>Patient 2: Als je zelf de tijd aan kunt geven dat het gelegen komt.
Repeating the same active tasks becomes boring	Bij elke test waar ik aan heb meegedaan moest ik altijd hetzelfde doen. Terugtellen van 100 met 7 naar beneden, altijd dezelfde test. Je moet eens een keer iets anders verzinnen.
Trust in measurement when the patient recognizes it	Interviewer: Stel dat de informatie die de sensoren verzamelt niet helemaal overeenkomt hoe je het zelf ervaart. Hoe zou u daarmee omgaan dan? Stel dat komt bij de neuroloog of fysiotherapeut en die zegt: ‘we zien dat een tremor of iets anders’ en u herkent dat niet.<br>Patient: Dan zeg je: wat een rotding is dat.
